# Iron(II)‐Catalyzed Aerobic Biomimetic Oxidation of N‐Heterocycles

**DOI:** 10.1002/chem.202102483

**Published:** 2021-09-06

**Authors:** Srimanta Manna, Wei‐Jun Kong, Jan‐E. Bäckvall

**Affiliations:** ^1^ Department of Organic Chemistry Arrhenius Laboratory Stockholm University 10691 Stockholm Sweden; ^2^ Department of Natural Sciences Mid Sweden University 85170 Sundsvall Sweden

**Keywords:** aerobic oxidation, biomimetic oxidation, electron transfer mediator, heterocycles, iron catalysis

## Abstract

Herein, an iron(II)‐catalyzed biomimetic oxidation of N‐heterocycles under aerobic conditions is described. The dehydrogenation process, involving several electron‐transfer steps, is inspired by oxidations occurring in the respiratory chain. An environmentally friendly and inexpensive iron catalyst together with a hydroquinone/cobalt Schiff base hybrid catalyst as electron‐transfer mediator were used for the substrate‐selective dehydrogenation reaction of various N‐heterocycles. The method shows a broad substrate scope and delivers important heterocycles in good‐to‐excellent yields.

## Graphical abstract

**Making a transfer**: An iron‐catalyzed, aerobic, biomimetic oxidation of various N‐heterocycles by using the bifunctional hybrid catalyst Co(Salophen)‐HQ as an electron‐transfer mediator (ETM) is reported. The reaction provides various important aromatic heterocycles in good‐to‐excellent yields. The dehydrogenation involves several electron‐transfer steps that are inspired by biological oxidation processes in which molecular oxygen (O_2_) is used as terminal oxidant.

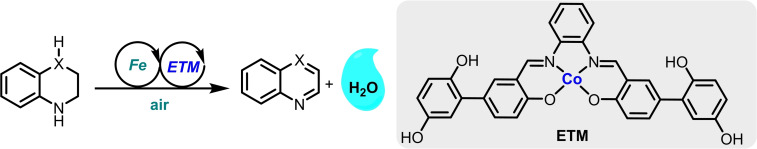

Dehydrogenation reactions constitute an important and fundamental class of reactions in organic chemistry.^[1]^ Over the past few decades, numerous transition metal‐catalyzed dehydrogenative reactions have been reported,^[2]^ and in these reactions there is room for improvement to obtain mild, efficient and scalable methods. Oxidation processes inspired by biological systems employing environmentally friendly and inexpensive oxidants such as molecular oxygen (O_2_) or hydrogen peroxide (H_2_O_2_) are increasing in demand.^[3]^ Direct oxidation of an organic substrate by O_2_ is an unfavored process and leads to low selectivity.^[4]^ A substrate‐selective redox catalyst (SSRC) can be used to solve this problem, however, direct re‐oxidation of the reduced form of the SSRC (i. e., SSRC_red_) by H_2_O_2_ or O_2_ only works well in a limited number of cases.[^3c^, ^3g^] By using electron transfer mediators (ETMs) the energy barrier for electron transfer from the SSRC_red_ to H_2_O_2_ or O_2_ may be dramatically lowered (Scheme 1).[^3c^, ^3g^]

**Scheme 1:**
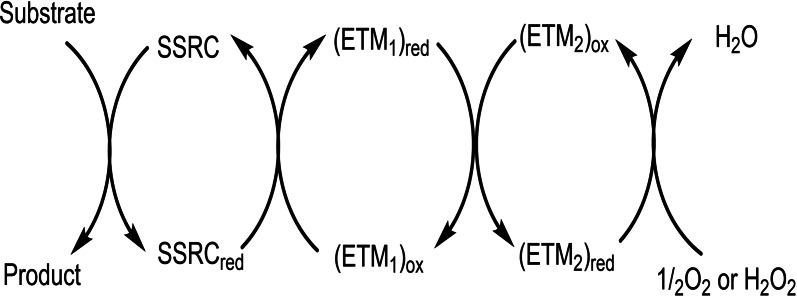
Principle of oxidation with O_2_ or H_2_O_2_ through the use of ETMs (ETM=electron transfer mediator; SSRC=substrate selective redox catalyst).

Nature's creation of various enzymatic and co‐enzymatic pathways has solved the problem of high‐energy electron transfer by using electron transfer mediators (ETMs), which lower the overall energy barrier for electron transfer from the SSRC_red_ to O_2_. In natural systems, these ETMs are part of the electron transport chain (respiratory chain), where oxidation by O_2_ occurs through stepwise electron transfer (cf. Scheme 1).^[5]^

Over the past few decades iron‐catalyzed reactions have found use in many synthetic transformations such as cross‐couplings^[6]^ and transfer hydrogenations,^[7]^ among many other reactions.^[8]^ In recent years iron‐catalyzed aerobic oxidations have emerged as valuable transformations in organic chemistry.^[9]^ Our group has been actively involved in developing biomimetic oxidations by using palladium,^[10]^ ruthenium,^[11]^ and osmium^[12]^ as substrate‐selective redox catalysts in similar electron transfer chains. Our group recently reported the iron‐catalyzed aerobic biomimetic oxidation of alcohols employing two electron transfer 2,6‐dimethoxy‐1,4‐benzoquinone (DMBQ) and the cobalt Schiff‐base catalyst (Co(salmdpt); cf. Scheme 2A, X=O in substrate).^[9b]^ Merging the two ETMs (the Co‐Schiff base and the quinone) into a bifunctional hybrid ETM, for example **IId**,[^10d^, ^13^] would increase the efficiency of the electron transfer (Scheme 2A). Very recently, our group reported an aerobic biomimetic oxidation of amines as a complementary route to prepare imines using iron catalyst **I** together with hybrid catalyst **IId** as ETM (Scheme 2B).^[13c]^ Historically (cyclopentadienone)iron tricarbonyl complexes **I** were first successfully synthesized by Reppe and Vetter in 1953.^[14]^ In 1999, Knölker reported on the synthesis of **III**,^[15]^ which belongs to a prominent class of iron hydride complexes. Later on, the group of Casey used Knölker's iron complex **III** in hydrogenation of ketones in 2007.^[16]^ Our group extensively utilized similar (cyclopentadienone)iron tricarbonyl complexes in both the dynamic kinetic resolution of *sec*‐alcohols and cycloisomerization of allenes.^[17]^ The activation of iron complex **Ia** to **Ia’** is done by trimethylamine N‐oxide by oxidative decarbonylation, and the latter intermediate **Ia’** is reduced to **IV** (Scheme 3).

**Scheme 2:**
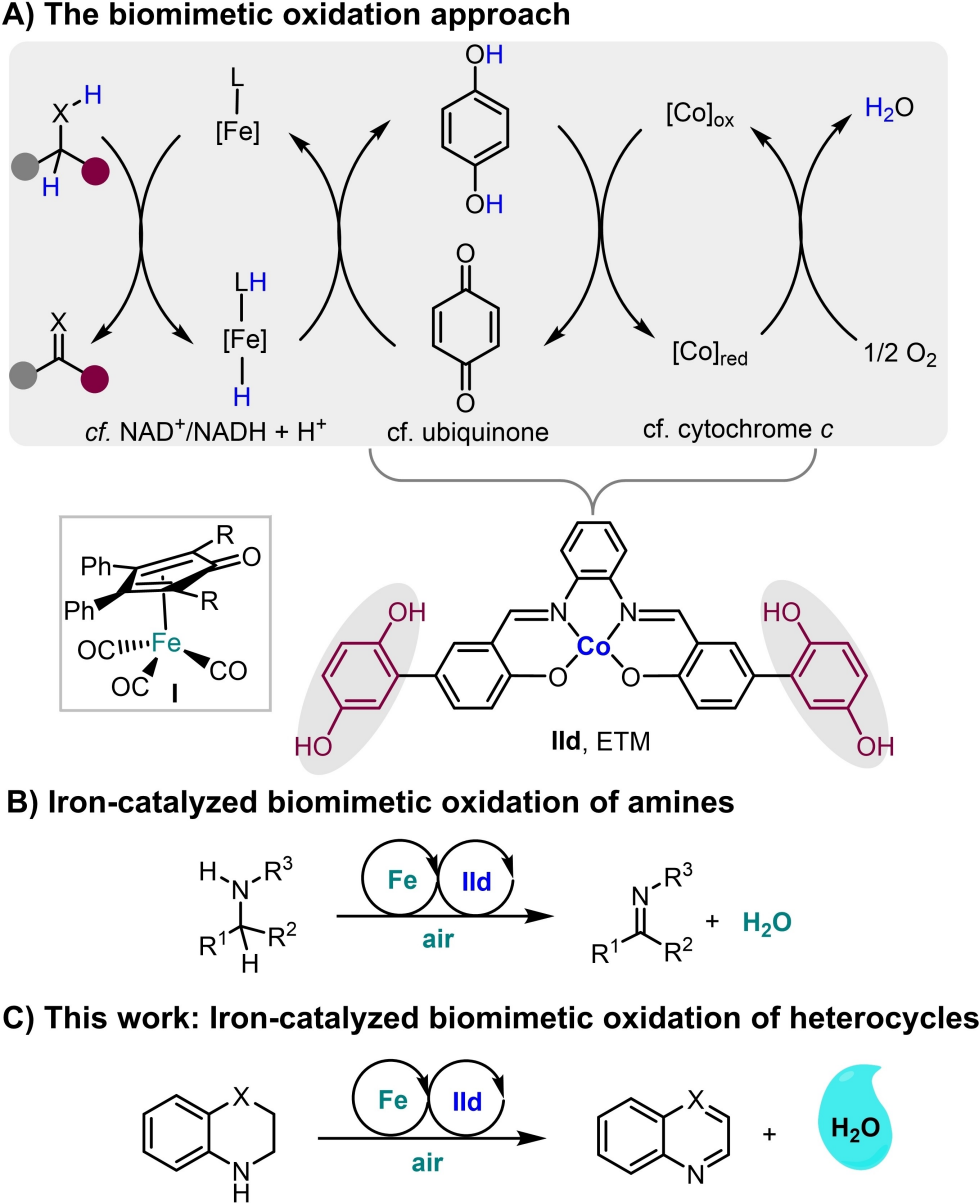
A) Biomimetic oxidation approach with iron catalyst **I** as the SSRC (X=O, N−R). B) Iron‐catalyzed biomimetic oxidation of amines. C) Iron‐ and hybrid hydroquinone/cobalt‐catalyzed biomimetic oxidation of N‐heterocycles.

**Scheme 3:**
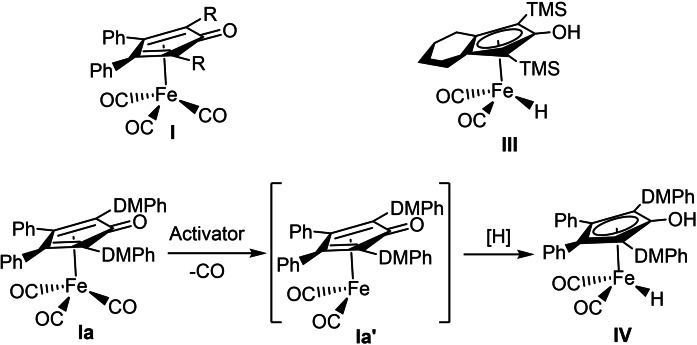
Activation of iron tricarbonyl complex **I** and Knölkers complex **III** (DMPh=3,5‐dimethylphenyl).

We envisaged that the reaction conditions for the iron‐catalyzed biomimetic aerobic oxidation of amines could also be used for the oxidation of N‐heterocycles (Scheme 2C). Metal‐catalyzed dehydrogenative reactions of 1,2,3,4‐tetrahydroquinoline have previously been reported.^[18]^ Herein, we report an iron‐catalyzed biomimetic aerobic oxidation of various N‐heterocycles using the bifunctional hybrid catalyst **IId** as an efficient ETM.

We began our studies by optimizing the iron‐catalyzed biomimetic oxidation of 1,2,3,4‐tetrahydroquinoline (**1 a**) using iron catalyst **Ia** and hybrid catalyst **IId** under aerobic conditions using an air balloon. To our delight, the desired product **2 a** was obtained in 24 % yield after 16 h at 100 °C in anisole (Table 1, entry 1). After screening reaction conditions with different solvents (entries 1–6), we found DMSO to be the best solvent (entry 3). Running the reaction for 36 h in DMSO at 100 °C, resulted in 75 % NMR yield (entry 7). After survey of different temperatures, we found that the use of 90 °C was optimal, which afforded **2 a** in 90 % yield (entry 8). A similar result was observed when the reaction was run under open air, affording **2 a** in 94 % yield (entry 9). When the reaction was carried out at 80 °C in DMSO as solvent, the yield of **2 a** was decreased to 83 % (entry 10). We next tried MeOH as solvent at 80 °C in a sealed tube and found that it is a good solvent affording product **2 a** in 81 % NMR yield (entry 11). We also investigated the effect of different bases, which may facilitate the putative isomerization of the initially formed imine to enamine (entries 12–14). However, these experiments did not lead to any improvement in yield of the desired product.

**Table 1:** Screening of reaction conditions.^[a]^

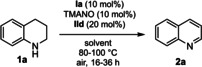					
	Solvent	*t* [h]	*T* [°C]	Additive	Yield [%]^[b]^
1^[c]^	anisole	16	100	–	24
2^[c]^	MeCN	16	100	–	15
3^[c]^	DMSO	16	100	–	52
4^[c]^	DMF	16	100	–	33
5^[c]^	DCE	16	100	–	11
6^[c]^	dioxane	16	100	–	30
7^[c]^	DMSO	36	100	–	75
8^[c]^	DMSO	36	90	–	90
**9** ^[d]^	**DMSO**	**36**	**90**	**–**	**94**
10^[c]^	DMSO	36	80	–	83
11^[e]^	MeOH	36	80	–	81
12^[c,f]^	DMSO	36	90	K_2_CO_3_	45
13^[c,f]^	DMSO	36	90	KO*t*Bu	83
14^[c,f]^	DMSO	36	90	NaOAc	41

[a] General reaction conditions: 0.15 mmol of **1 a**, 0.015 mmol of **Ia**, 0.03 mmol of **IId**, 0.015 mmol of TMANO, and solvent (2 mL) under air. [b] Yields were determined by ^1^H NMR analysis using 1,3,5‐trimethoxybenzene as internal standard. [c] Reaction carried out under air connection by using an air balloon. [d] Reaction was performed under open air. [e] Reaction was carried out under air in 25 mL sealed tube [f] 20 mol% additive was used. DCE=1,2‐dichloroethane. Dioxane=1,4‐dioxane.

Next, we examined different electron transfer mediators (ETMs) in the dehydrogenative reaction (Table 2). Of the tested ETMs, **IIa**–**IIh**, hybrid hydroquinone/cobalt Schiff base **IId** provided the best result, 94 % yield of **2 a**, whereas the use of separate ETMs **IIa** and **IIb** afforded **2 a** in 75 % yield (Table 2). We next investigated different iron complexes in the biomimetic reaction (Table S5 in the Supporting Information) as potential catalysts. By screening the iron complexes, we found that the application of iron complex **Ia** as a precatalyst in the oxidation of tetrahydroquinoline **1 a** was optimal, which afforded product **2 a** in 94 % NMR yield (entry 9, Table 1).

**Table 2:** Screening of ETMs.^[a]^

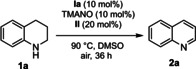
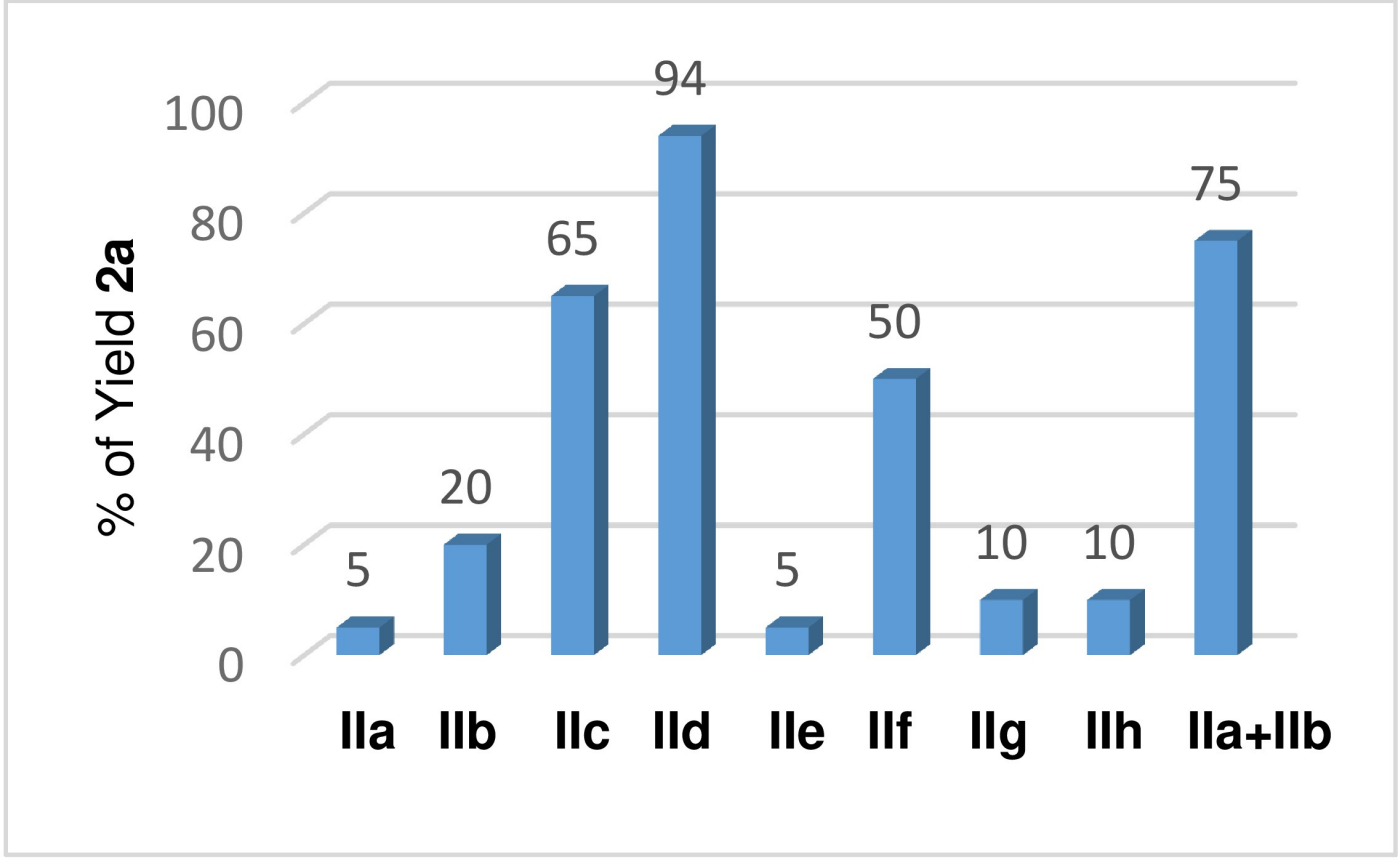
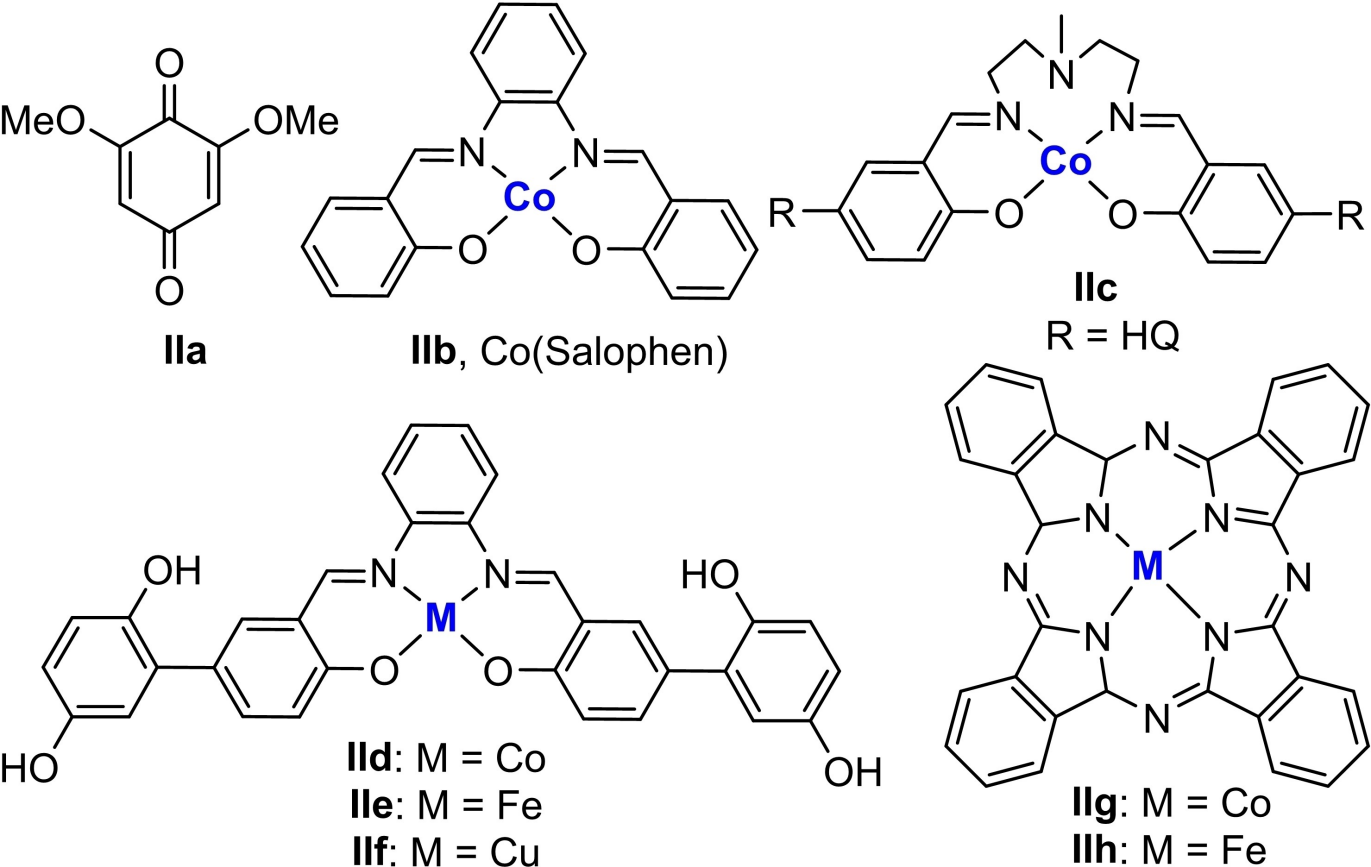

[a] General reaction conditions: 0.15 mmol of **1 a**, 0.015 mmol of **Ia**, 0.03 mmol of **II** and 0.015 mmol of TMANO in DMSO (2 mL) under air. Yields were determined by ^1^H NMR analysis using 1,3,5‐trimethoxybenzene. HQ=1,4‐hydroquinone.

With the optimized reaction conditions in hand, we next explored the substrate scope of the biomimetic oxidation of N‐heterocycles. First, we explored the scope of various tetrahydroquinoline derivatives (Scheme 4). We were pleased to find that the application of various electron‐donating and electron‐withdrawing groups on the tetrahydroquinolines led to formation of the desired products in good to high yields (**2 a**–**2 j**). Various groups such as chloro, methoxy and methyl groups on the tetrahydroquinolines were well tolerated under the optimized conditions. For example, a methoxy group at the 6‐position on tetrahydroquinoline was well tolerated under the reaction conditions, and the desired product **2 h** was obtained in an excellent yield of 96 %. Dinitrogen‐containing heterocyclic compounds also worked in an excellent manner and afforded heterocycles in high yields. Pleasingly, we found that various functional groups such as chloro, methyl and phenyl on these dinitrogen‐containing heterocycles were well tolerated under the optimized reaction conditions (**2 k**–**2 n**).

**Scheme 4:**
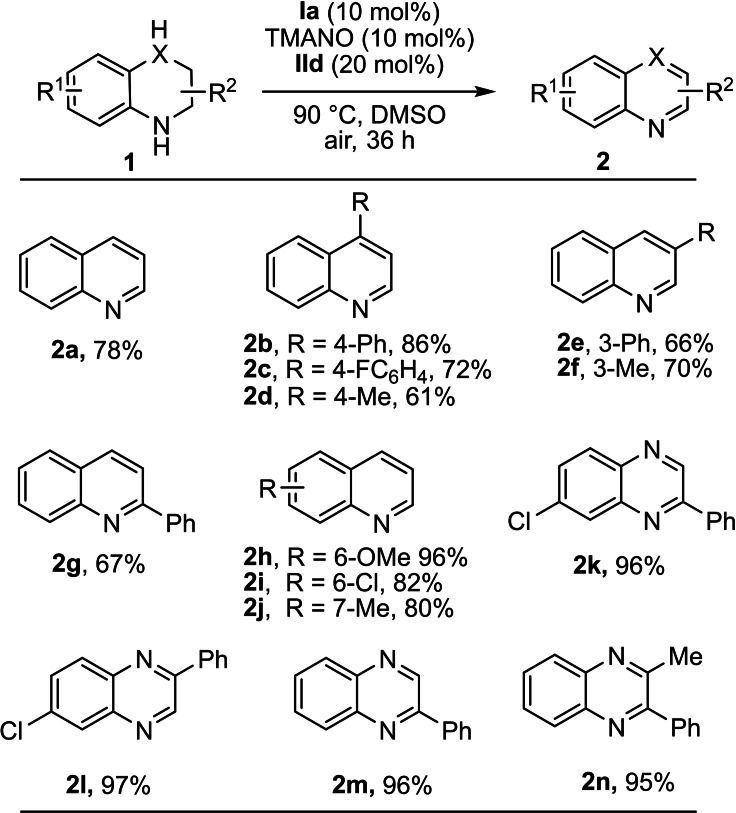
Scope with respect to the N‐heterocycles. General reaction conditions: 0.15 mmol of **1**, 0.015 mmol of **Ia**, 0.03 mmol of **IId** and 0.015 mmol of TMANO in DMSO (2 mL) under air. Isolated yields are given.

Furthermore, selective oxidations of additional nitrogen‐containing heterocycles were examined under the developed reaction conditions (Scheme 5). We were pleased to find that the application of various nitrogen‐containing heterocycles led to formation of the desired products in good to excellent yields (**4 a**–**4 e**). Notably, Hantzsch ester **3 c** was well tolerated under the developed reaction conditions and delivered the corresponding product **4 c** in excellent isolated yield (98 %). The dehydrogenation of 9,10‐dihydroacridine **3 d** occurred smoothly and the corresponding product **4 d** was obtained in excellent isolated yield (99 %).

**Scheme 5:**
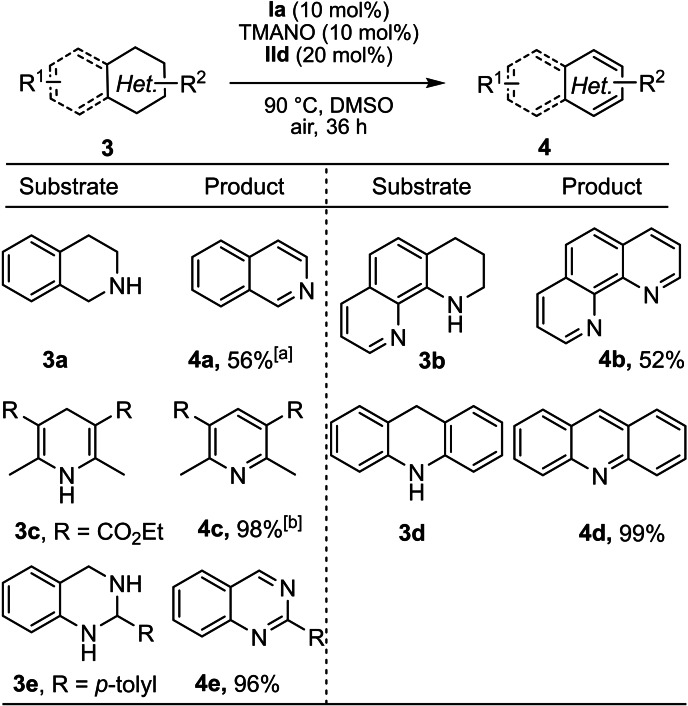
Scope with respect to the other N‐heterocycles. General reaction conditions: 0.15 mmol of **3**, 0.015 mmol of **Ia**, 0.03 mmol of **IId** and 0.015 mmol of TMANO in DMSO (2 mL) under air. Isolated yields are given. [a] Reaction run for 48 h. [b] Reaction run for 12 h.

Having successfully applied the biomimetic oxidation to tetrahydroquinolines and other six‐membered nitrogen‐containing heterocycles, a few indoline derivatives were investigated (Scheme 6). Substituted indoline derivatives worked well under the optimized reaction conditions and delivered the corresponding products (**6 a**–**6 f**) in good to excellent yields. Unfortunately, electron‐withdrawing groups such as nitro in the 5‐ or 6‐position of the indolines provided only trace amounts of the desired products (see the Supporting Information).

**Scheme 6:**
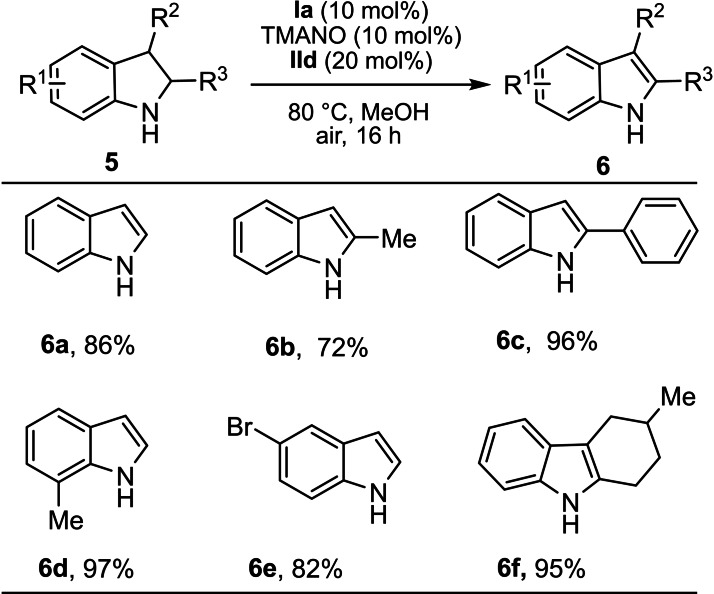
Scope with respect to indolines. General reaction conditions: 0.15 mmol of **5**, 0.015 mmol of **Ia**, 0.015 mmol of TMANO and 0.03 mmol of **IId** in MeOH (2 mL) at 80 °C under air for 16 h. Isolated yields are given.

We applied polycyclic N‐heterocycle **7 a** in the biomimetic oxidation to give **8 a** (Scheme 7). Interestingly, we observed both dehydrogenation and benzylic oxygenation to afford indeno[2,1‐*c*] quinoline product **8 a** in 69 % isolated yield. 1,2,3,4‐Tetrahydroquinoline **7 a** was obtained in 65 % yield from hetero Diels‐Alder reaction of indene and in situ generated CH_2_=NPh (from rearranged benzyl azide).[^18f^, ^19^] Indeno[2,1‐*c*] quinoline derivatives such as **8 a** are an important class of heterocycles that are structural elements in numerous bioactive compounds.^[20]^

**Scheme 7:**
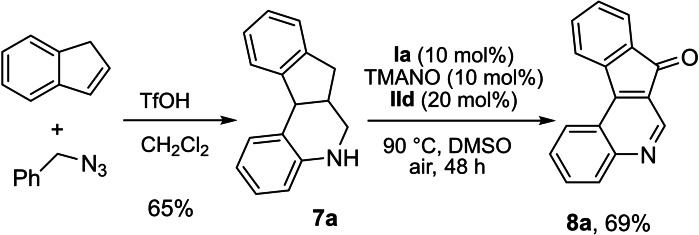
Reaction with polycyclic N‐heterocycle. Reaction conditions: **7 a** (0.15 mmol), **Ia** (0.015 mmol), **IId** (0.03 mmol), TMANO (0.015 mmol) in DMSO (2 mL) at 90 °C for 48 h under air. The isolated yield is given.

We next, tested the biomimetic oxidation in a one‐pot reaction of aldehyde and functionalized aryl amine components (Scheme 8).^[21]^ A range of quinazoline derivatives were generated from diamine **9** and aldehydes **10** in 60–78 % yields under the optimized reaction conditions (**11 a**–**11 c**). Heterocycles such as benzoxazole, benzothiazole and benzimidazole derivatives **14 a**–**c** were obtained in good to excellent yields from **12** and **13** with a lower catalyst loading.

**Scheme 8:**
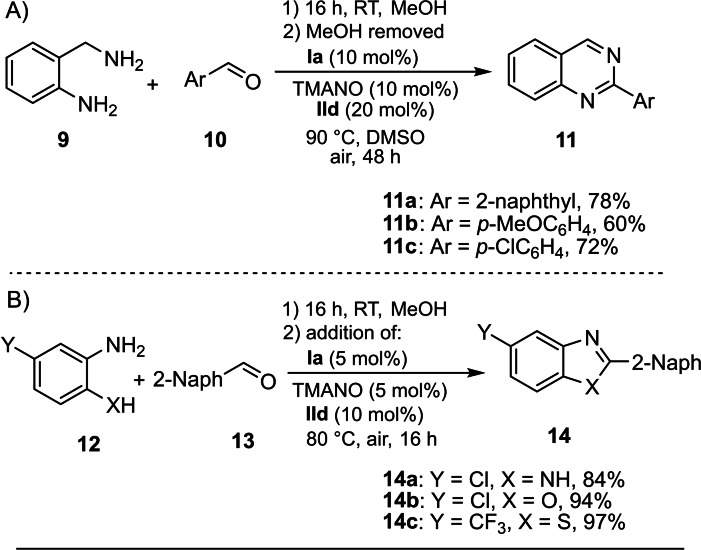
Scope of one‐pot reaction. Reaction conditions: A) Step 1, **9** (0.15 mmol) and **10** (0.15 mmol) in MeOH (2 mL) at RT for 16 h; Step 2, 0.015 mmol of **Ia**, 0.015 mmol of TMANO and 0.03 mmol of **IId** in DMSO (2 mL) (after removal of MeOH in vacuum), 90 °C, 48 h under air. B) Step 1, **12** (0.15 mmol) and **13** (0.15 mmol) in MeOH (2 mL) at RT for 16 h; Step 2, 0.0075 mmol of **Ia**, 0.0075 mmol of TMANO and 0.015 mmol of **IId** were added to the MeOH solution, and the reaction was run for 16 h at 80 °C under air. Isolated yields are given.

Based on our studies, a plausible mechanism is proposed in Scheme 9. The initially activated iron complex **Ia’** is generated by oxidative decarbonylation with TMANO. In the following step, the active catalyst species **Ia’** reacts with **1 a** to furnish the iron hydride complex **IV** and imine intermediate **15**. The imine **15** undergoes an isomerization to intermediate **17**, via intermediate **16**. Product **2 a** would be obtained from **17** by an iron‐catalyzed aerobic oxidation (cf. **1**→**15**). The iron hydride intermediate **IV** reacts with an oxidized form of the hybrid catalyst (**IId_ox_**) to regenerate **Ia’**. The reduced form of hybrid catalyst (**IId_red_**) reacts with molecular oxygen to give an oxidized hybrid catalyst (**IId_ox_**). For the details of the different intermediates involved in the reoxidation of **IId_red_** to **IId_ox_**, see our previous report.^[13c]^

**Scheme 9:**
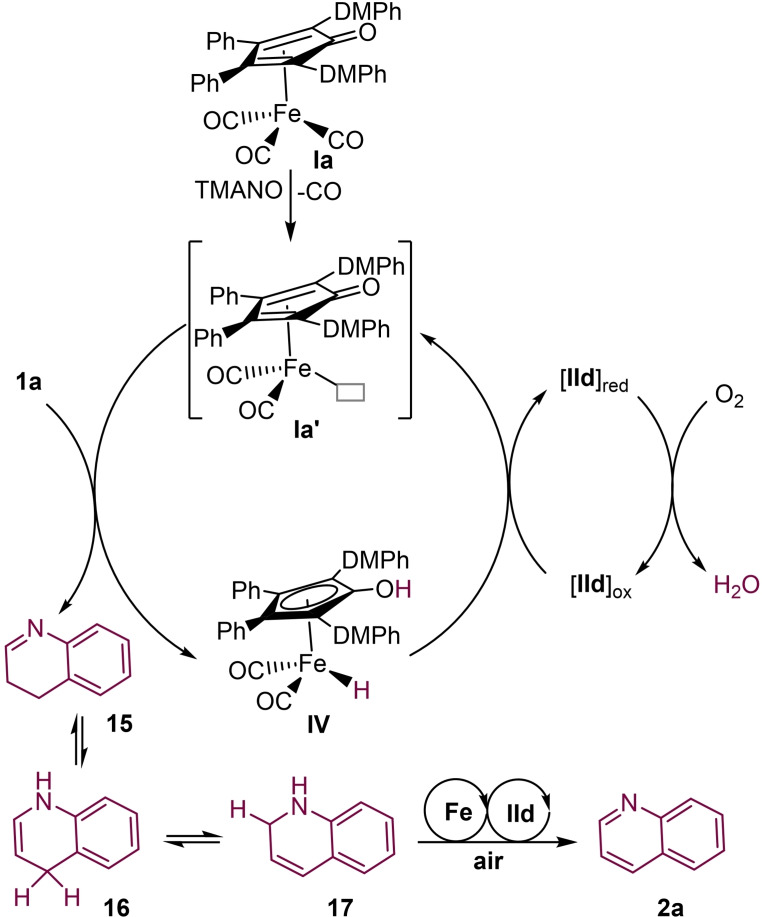
Proposed reaction mechanism.

In conclusion, we have developed an iron‐catalyzed biomimetic oxidation of N‐heterocycles under aerobic conditions by using a bifunctional hybrid catalyst (Co(Salophen)‐HQ) as an efficient electron transfer mediator (ETM). A range of N‐heterocycles were oxidized to their corresponding aromatic heterocycles in good‐to‐excellent yields by using this method.

## Conflict of interest

The authors declare no conflict of interest.

## Supporting information

As a service to our authors and readers, this journal provides supporting information supplied by the authors. Such materials are peer reviewed and may be re‐organized for online delivery, but are not copy‐edited or typeset. Technical support issues arising from supporting information (other than missing files) should be addressed to the authors.

Supporting Information
