# Supplementary material for: Iron(II)‐Catalyzed Aerobic Biomimetic Oxidation of N‐Heterocycles
Source: Chemistry. 2021 Sep 6;27(55):13725–9. doi: 10.1002/chem.202102483 (PMC8518507; doi:10.1002/chem.202102483)
Supplement: Supplementary file 1 — Supporting Information [file CHEM-27-13725-s001.pdf]

Chemistry–A European Journal

Supporting Information

Iron(II)-Catalyzed Aerobic Biomimetic Oxidation of N-Heterocycles

Srimanta Manna[+], Wei-Jun Kong[+], and Jan-E. Bäckvall*

Table of Contents

1. General Information S2

2. General procedure A S2

3. General procedure B S2

4. Optimization studies S3

5.Unsuccessful indolines S9

5. Characterization data of products S10

6. NMR spectra of synthesized compounds S25

7. References S57

General information

^{1}H NMR and ^{13}C NMR spectral data were recorded at 400 MHz and 100 MHz, respectively. Chemical shifts (δ) are reported in ppm, using the residual solvent peak in $CDCl_3$ (H = 7.26 and C = 77.0 ppm) as internal standard, and coupling constants (*J*) are given in Hz. Silica gel chromatography was performed manually (particle size 40-63 µm, pore size 60 Å and mesh size 230-400) using silica. Reactions were monitored using aluminum-backed plates (1.5 Å, 5 cm) pre-coated (0.25 mm) with silica gel, UV light or potassium permanganate stain for visualization. Iron complex **I** was synthesized using an established procedure.[1] Catalyst **IId** was synthesized using reported literature.[2]

General procedure A for the iron(II)-catalyzed biomimetic oxidation

To a flame dried Schlenk flask was added **IId** (17.7 mg, 0.03 mmol), TMANO (1.2 mg, 0.015 mmol), **Ia** (8.9 mg, 0.015 mmol), dry DMSO (2.0 mL) and substrate (0.15 mmol) under air. Then, the reaction mixture was run under open air for 36 hours at 90 °C. Upon completion, reaction mixture was worked-up with Et_2O (20 mL x 3), organic layer washed with brine (20 mL) and dried with Na_2SO_4. Reaction mixture was concentrated in vacuo and the crude product was purified by column chromatography.

General procedure B for the iron(II)-catalyzed biomimetic oxidation

To a 25 mL flame dried sealed tube was added **IId** (17.7 mg, 0.03 mmol), TMANO (1.2 mg, 0.015 mmol), **Ia** (8.9 mg, 0.015 mmol), dry MeOH (2.0 mL) and substrate (0.15 mmol) under air. Then, the sealed tube was fitted with Screw cap and was run for 16 h stirred at 80 °C under air. Upon completion, solvent was removed in vacuo and the crude product was purified by column chromatography.

Optimization studies

Table S1. Optimization of solvents[a]

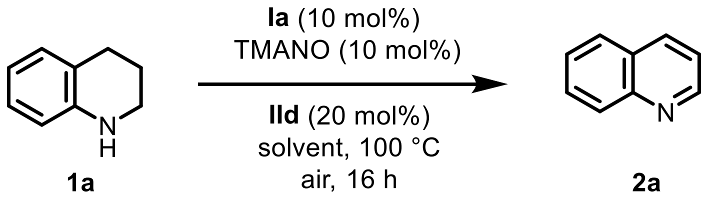

Entry	Time (h)	Solvent (2 mL)	NMR Yield (%)[b]
1	16	MeCN	15
2	16	DCE	11
3	16	DMSO	52
4	16	Dioxane	38
5	16	DMF	33
6	16	MeOH (RT)	26
**7**	**36**	**DMSO**	**75**

[a] General reaction conditions: 0.15 mmol of **1a**, 0.015 mmol of **Ia**, 0.03 mmol of **IId**, 0.015 mmol of TMANO, and solvent (2 mL) at 100 °C 16 h under air (fitted with an air-filled balloon). [b] Yields were determined by ^{1}H NMR analysis using 1,3,5-trimethoxybenzene.

Table S2. Optimization of temperature[a]

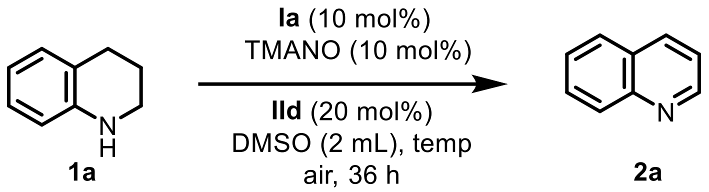

Entry	Temp (°C)	NMR Yield (%)[b]
1	60	48
2	80	83
3	90	90
4	100	75
5[c]	80	81
**6[d]**	**90**	**94**

[a] General reaction conditions: 0.15 mmol of **1a**, 0.015 mmol of **Ia**, 0.03 mmol of **IId**, 0.015 mmol of TMANO, and DMSO (2 mL) at temp °C for 36 h under air (fitted with an air-filled balloon). [b] Yields were determined by ^{1}H NMR analysis using 1,3,5-trimethoxybenzene. [c] Reaction carried out in MeOH (2 mL) for 36 h. [d] Reaction was performed under open air.

Table S3. Optimization of ETMs[a]

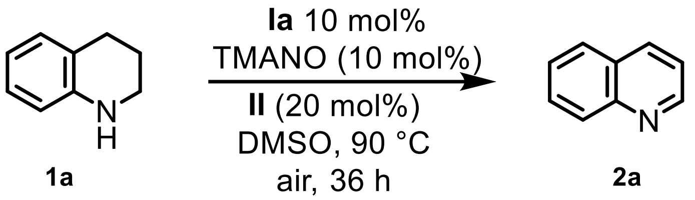

Entry	ETM (20 mol%)	NMR Yield (%)[b]
1	**IIa**	5
2	**IIb**	20
3	**IIc**	65
**4**	**IId**	**94**
5	**IIe**	5
6	**IIf**	50
7	**IIg**	10
8	**IIh**	10

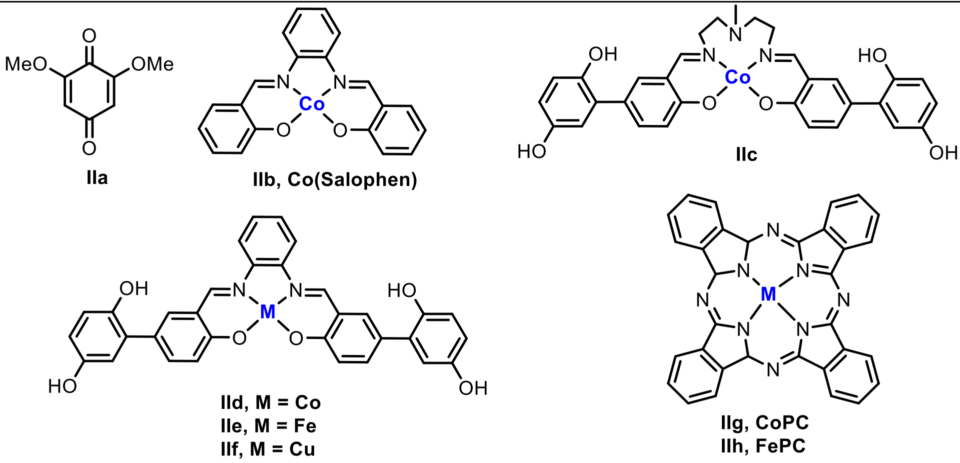

[a] General reaction conditions: 0.15 mmol of **1a**, 0.015 mmol of **Ia**, 0.03 mmol of **II**, 0.015 mmol of TMANO, and DMSO (2 mL) at 90 °C for 36 h under air. [b] Yields were determined by ^{1}H NMR analysis using 1,3,5-trimethoxybenzene.

Table S4: Optimization of additive[a]

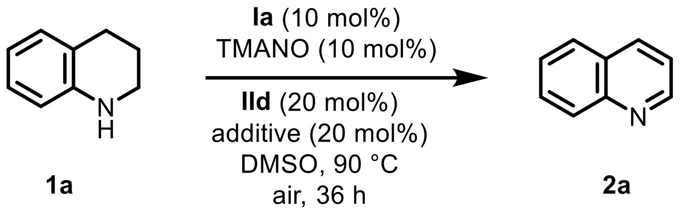

Entry	Additive (20 mol%)	Oxidant (10 mol%)	NMR Yield (%)[b]
**1**	**-**	**TMANO**	**94**
2	-	-	80
3	K_2CO_3	TMANO	45
4	KO*t*Bu	TMANO	83
5	NaI	TMANO	64
6	*n*Bu_4NI	TMANO	73
7	NaOAc	TMANO	41

[a] General reaction conditions: 0.15 mmol of **1a**, 0.015 mmol of **Ia**, 0.03 mmol of **IId**, 0.015 mmol of TMANO, additive (0.03 mmol) and DMSO (2 mL) at 90 °C for 36 h under air. [b] Yields were determined by ^{1}H NMR analysis using 1,3,5-trimethoxybenzene.

Table S5. Optimization of iron complexes[a]

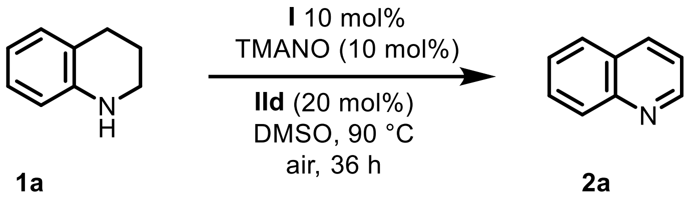

Entry	[Fe]	Yield (%)[b]
**1**	**Ia**	**94**
2	**Ib**	65
3	**Ic**	29
4	**Id**	20

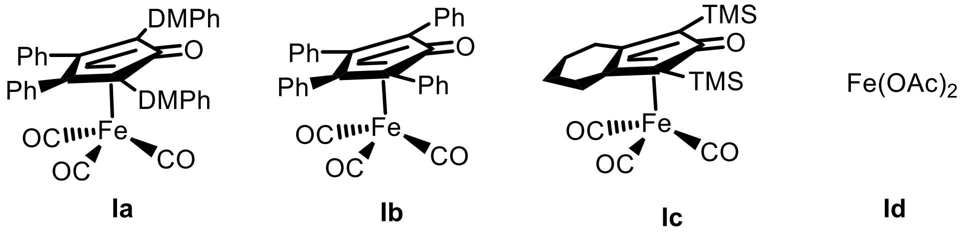

[a] General reaction conditions: 0.15 mmol of **1a**, 0.015 mmol of **I**, 0.03 mmol of **IId**, 0.015 mmol of TMANO, and DMSO (2 mL) at 90 °C for 36 h under air. [b] Yields were determined by ^{1}H NMR analysis using 1,3,5-trimethoxybenzene.

S6. Control experiments[a]

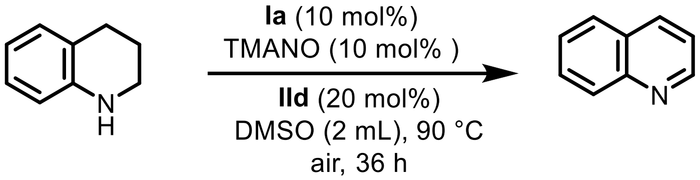

Entry	[Fe]	ETM (%)	Yield (%)[b]
1	10	-	trace
2	10	Co-Salophen **IIb** (20%)	20
3	10	HQ (40%)	23
**4**	**10**	**Hybrid (IId) (20%)**	**94**
5	10	Co-Salophen **IIb** (20%) + HQ (40%)	65
6	-	Hybrid (**IId**)	34
7	10	Hybrid (**IId**) (10%)	70
8	5	Hybrid (**IId**) (20%)	60
9[c]	10	Hybrid (**IId**) (20%)	25
10[c]	-	Hybrid (**IId**) (20%)	<5%

[a] General reaction conditions: 0.15 mmol of 1a, 0.015 mmol of I, xx mmol of ETM, 0.015 mmol of TMANO, and DMSO (2 mL) at 90 °C for 36 h under air. [b] Yields were determined by ^{1}H NMR analysis using 1,3,5-trimethoxybenzene. [c] Reaction run under nitrogen atmosphere. [d] Reaction was carried out under nitrogen atmosphere with degas DMSO (2 mL).

Unsuccessful indolines

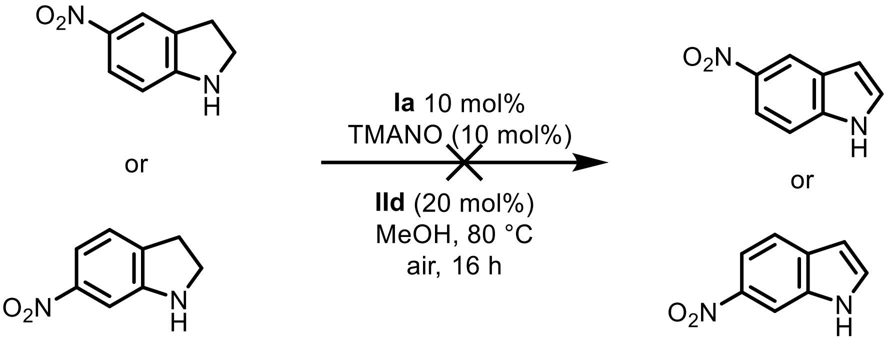

Characterization data of products

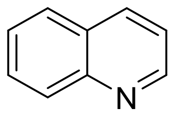

Quinoline (2a)

2a was prepared according to the general procedure A for iron(II)-catalyzed biomimetic oxidation. Eluent system for column chromatography: *n*-pentane/EtOAc 100/0 - 10/90 (v/v). Spectral data is consistent with data reported in the literature. Isolated yield: 78% (15.0 mg).

^{1}H NMR (400 MHz, CDCl$_3$) δ ppm 8.92 (dd, *J* = 4.2, 1.7 Hz, 1H), 8.13 (ddt, *J* = 15.1, 8.5, 1.0 Hz, 2H), 7.82 (dd, *J* = 8.2, 1.5 Hz, 1H), 7.72 (ddd, *J* = 8.4, 6.9, 1.5 Hz, 1H), 7.54 (ddd, *J* = 8.1, 6.9, 1.2 Hz, 1H), 7.39 (dd, *J* = 8.3, 4.2 Hz, 1H).

^{13}C NMR (101 MHz, CDCl$_3$) δ ppm 150.5, 148.4, 136.2, 129.6, 129.6, 128.4, 127.9, 126.7, 121.2.

The spectral data are consistent with those reported in the literature[3]

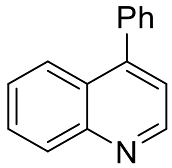

4-Phenylquinoline (2b)

2b was prepared according to the general procedure A for iron(II)-catalyzed biomimetic oxidation. Eluent system for column chromatography: *n*-pentane/EtOAc 95/5 - 15/85 (v/v). Isolated yield: 86% (26.5 mg).

^{1}H NMR (400 MHz, CDCl$_3$) δ ppm 8.95 (d, *J* = 4.4 Hz, 1H), 8.18 (dd, *J* = 8.5, 1.2 Hz, 1H), 7.93 (dd, *J* = 8.5, 1.4 Hz, 1H), 7.73 (ddd, *J* = 8.4, 6.8, 1.5 Hz, 1H), 7.59 – 7.45 (m, 6H), 7.35 (d, *J* = 4.4 Hz, 1H).

^{13}C NMR (101 MHz, CDCl$_3$) δ ppm 150.1, 148.8, 148.6, 138.2, 130.0, 129.7, 129.5, 128.7, 128.6, 126.9, 126.8, 126.0, 121.5.

The spectral data are consistent with those reported in the literature.[3]

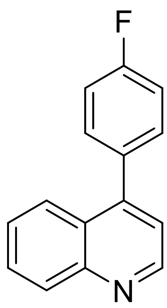

4-(4-Fluorophenyl)quinoline (2c)

2c was prepared according to the general procedure A for iron(II)-catalyzed biomimetic oxidation. Eluent system for column chromatography: *n*-pentane/EtOAc 95/5 - 15/85 (v/v). Isolated yield: 72% (24.1 mg).

^{1}H NMR (400 MHz, $CDCl_3$) δ ppm 8.97 (d, *J* = 4.4 Hz, 1H), 8.21 (dd, *J* = 8.5, 1.3 Hz, 1H), 7.90 (dd, *J* = 8.4, 1.3 Hz, 1H), 7.78 (d, *J* = 1.4 Hz, 1H), 7.61 – 7.47 (m, 3H), 7.34 (d, *J* = 4.4 Hz, 1H), 7.29 – 7.14 (m, 2H).

^{13}C NMR (101 MHz, $CDCl_3$) δ ppm 163.1 (d, *J* = 248.3 Hz), 150.1, 148.8, 147.5, 134.1 (d, *J* = 3.3 Hz), 131.4 (d, *J* = 8.1 Hz), 130.1, 129.2, 126.9, 126.9, 125.7, 115.9, 115.7. The spectral data are consistent with those reported in the literature.[3]

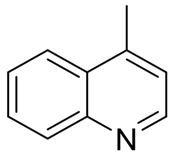

4-Methylquinoline (2d)

2d was prepared according to the general procedure A for iron(II)-catalyzed biomimetic oxidation. Eluent system for column chromatography: *n*-pentane/EtOAc 98/2 – 95/5 (v/v). Isolated yield: 61% (13.1 mg).

^{1}H NMR (400 MHz, $CDCl_3$) δ ppm 8.80 (d, *J* = 4.4 Hz, 1H), 8.13 (dt, *J* = 8.5, 1.0 Hz, 1H), 8.02 (dd, *J* = 8.5, 1.4 Hz, 1H), 7.73 (ddd, *J* = 8.4, 6.8, 1.4 Hz, 1H), 7.59 (ddd, *J* = 8.3, 6.9, 1.3 Hz, 1H), 7.25 (dd, *J* = 4.4, 1.0 Hz, 1H), 2.73 (d, *J* = 1.0 Hz, 3H).

^{13}C NMR (101 MHz, $CDCl_3$) δ ppm 150.3, 148.1, 144.4, 130.2, 129.2, 128.4, 126.4, 123.9, 122.0, 18.8. The spectral data are consistent with those reported in the literature.[4]

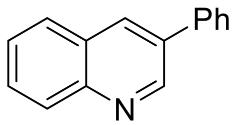

3-Phenylquinoline (2e)

2e was prepared according to the general procedure A for iron(II)-catalyzed biomimetic oxidation using a 25 mL Schenck tube with the side outlet open to air. Eluent system for column chromatography: *n*-pentane/EtOAc 10/1 (V/V). Isolated yield: 66% (20.3 mg).

^{1}**H NMR (400 MHz, CDCl$_3$)** δ ppm 9.19 (d, *J* = 2.3 Hz, 1H), 8.31 (d, *J* = 2.0 Hz, 1H), 8.18 – 8.12 (m, 1H), 7.89 (dd, J = 8.1, 1.3 Hz, 1H), 7.77 – 7.69 (m, 3H), 7.64 – 7.50 (m, 3H), 7.49 – 7.38 (m, 1H).

^{13}**C NMR (100 MHz, CDCl$_3$)** δ ppm 149.9, 147.3, 137.9, 133.9, 133.3, 129.4, 129.2, 129.2, 128.1, 128.0, 128.0, 127.4, 127.0. The spectral data are consistent with those reported in the literature.[4]

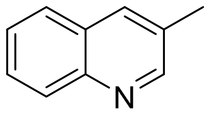

3-Methylquinoline (2f)

2f was prepared according to the general procedure A for iron(II)-catalyzed biomimetic oxidation using a 25 mL Schenck tube with the side outlet open to air. Eluent system for column chromatography: *n*-pentane/EtOAc 95/5 – 90/10 (V/V). Isolated yield: 70% (15.0 mg).

^{1}**H NMR (400 MHz, CDCl$_3$)** δ ppm 8.78 (d, *J* = 2.2 Hz, 1H), 8.07 (d, *J* = 8.5 Hz, 1H), 7.94 – 7.89 (m, 1H), 7.74 (dd, *J* = 8.1, 1.2 Hz, 1H), 7.64 (ddd, *J* = 8.4, 6.9, 1.4 Hz, 1H), 7.51 (ddd, *J* = 8.1, 6.9, 1.2 Hz, 1H), 2.55 – 2.50 (m, 3H).

^{13}**C NMR (100 MHz, CDCl$_3$)** δ ppm 152.4, 146.6, 134.7, 130.5, 129.2, 128.4, 128.1, 127.1, 126.5, 18.74. The spectral data are consistent with those reported in the literature.[4]

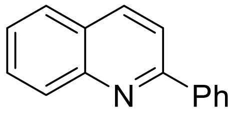

2-Phenylquinoline (2g)

2g was prepared according to the general procedure A for iron(II)-catalyzed biomimetic oxidation using a 25 mL Schenck tube with the side outlet open to air. Eluent system for column chromatography: *n*-pentane/EtOAc 95/5 – 90/10 (V/V). Isolated yield: 67% (20.6 mg).

^{1}**H NMR (400 MHz, CDCl$_3$)** δ ppm 8.25 – 8.14 (m, 4H), 7.88 (d, *J* = 8.6 Hz, 1H), 7.83 (dd, *J* = 8.1, 1.3 Hz, 1H), 7.74 (ddd, *J* = 8.4, 6.9, 1.4 Hz, 1H), 7.59 – 7.44 (m, 4H).

^{13}**C NMR (100 MHz, CDCl$_3$)** δ ppm 157.3, 148.3, 139.7, 136.7, 129.7, 129.6, 129.3, 128.8, 127.5, 127.4, 127.2, 126.2, 119.0. The spectral data are consistent with those reported in the literature.[4]

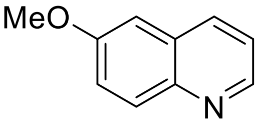

6-Methoxyquinoline (2h)

2h was prepared according to the general procedure A for iron(II)-catalyzed biomimetic oxidation using a 25 mL Schenck tube with the side outlet open to air. Eluent system for column chromatography: *n*-pentane/EtOAc 10/1 (V/V). Isolated yield: 96% (23.0 mg).

^{1}H NMR (400 MHz, $CDCl_3$) δ ppm 8.77 (dd, *J* = 4.2, 1.7 Hz, 1H), 8.08 – 8.03 (m, 1H), 8.00 (d, *J* = 9.2 Hz, 1H), 7.41 – 7.32 (m, 2H), 7.07 (d, *J* = 2.8 Hz, 1H), 3.94 (s, 3H).

^{13}C NMR (100 MHz, $CDCl_3$) δ = 157.7, 148.0, 144.5, 134.8, 130.9, 129.3, 122.3, 121.4, 105.1 55.5. The spectral data are consistent with those reported in the literature.[4]

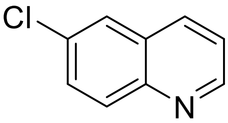

6-chloroquinoline (2i)

2i was prepared according to the general procedure A for iron(II)-catalyzed biomimetic oxidation using a 25 mL Schenck tube with the side outlet open to air. Eluent system for column chromatography: *n*-pentane/EtOAc 95/5 – 90/10 (V/V). Isolated yield: 82% (20.0 mg).

^{1}H NMR (400 MHz, $CDCl_3$) δ ppm 8.89 (d, *J* = 3.0 Hz, 1H), 8.04 (t, *J* = 7.9 Hz, 2H), 7.80 – 7.76 (m, 1H), 7.67 – 7.60 (m, 1H), 7.40 (dd, *J* = 8.2, 4.1 Hz, 1H).

^{13}C NMR (100 MHz, $CDCl_3$) δ = 150.6, 146.6, 135.1, 132.3, 131.1, 130.4, 128.8, 126.4, 121.9. The spectral data are consistent with those reported in the literature.[4]

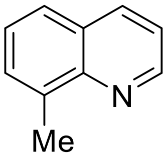

8-methylquinoline (2j)

2j was prepared according to the general procedure A for iron(II)-catalyzed biomimetic oxidation using a 25 mL Schenck tube with the side outlet open to air. Eluent system for column chromatography: *n*-pentane/EtOAc 95/5 – 90/10 (V/V). Isolated yield: 80% (17.2 mg).

^{1}H NMR (400 MHz, $CDCl_3$) δ ppm 8.95 (dd, *J* = 4.2, 1.8 Hz, 1H), 8.14 (dd, *J* = 8.2, 1.8 Hz, 1H), 7.67 (d, *J* = 8.1 Hz, 1H), 7.60 – 7.52 (m, 1H), 7.48 – 7.36 (m, 2H), 2.83 (s, 3H).

^{13}C NMR (100 MHz, CDCl$_3$) δ = 149.3, 147.4, 137.1, 136.3, 129.6, 128.3, 126.3, 125.9, 120.8, 18.1. The spectral data are consistent with those reported in the literature.[4]

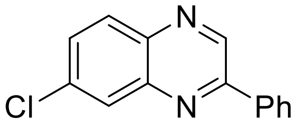

7-Chloro-2-phenylquinoxaline (2k)

2k was prepared according to the general procedure A for iron(II)-catalyzed biomimetic oxidation. Eluent system for column chromatography: *n*-pentane/EtOAc 95/5 – 80/20 (v/v). Isolated yield: 96% (34.7 mg).

^{1}H NMR (400 MHz, CDCl$_3$) δ ppm 9.32 (s, 1H), 8.26 – 8.12 (m, 3H), 8.06 (d, *J* = 8.9 Hz, 1H), 7.69 (dd, *J* = 8.9, 2.3 Hz, 1H), 7.61 – 7.49 (m, 3H).

^{13}C NMR (101 MHz, CDCl$_3$) δ ppm 152.7, 143.6, 142.8, 140.3, 136.5, 136.3, 130.7, 130.7, 130.5, 129.4, 128.7, 127.8. The spectral data are consistent with those reported in the literature.[5]

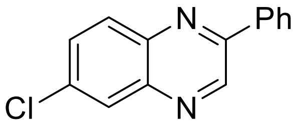

6-Chloro-2-phenylquinoxaline (2l)

2l was prepared according to the general procedure A for iron(II)-catalyzed biomimetic oxidation. Eluent system for column chromatography: *n*-pentane/EtOAc 95/5 – 80/20 (v/v). Isolated yield: 97% (35.0 mg).

^{1}H NMR (400 MHz, CDCl$_3$) δ ppm 9.33 (s, 1H), 8.25 – 8.16 (m, 2H), 8.14 – 8.06 (m, 2H), 7.73 (dd, *J* = 9.0, 2.4 Hz, 1H), 7.62 – 7.47 (m, 3H).

^{13}C NMR (101 MHz, CDCl$_3$) δ ppm 152.1, 144.3, 142.0, 141.0, 136.5, 135.4, 131.5, 131.0, 130.6, 129.4, 128.2, 127.7. The spectral data are consistent with those reported in the literature.[5]

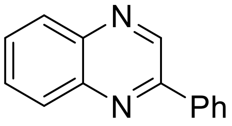

2-Phenylquinoxaline (2m)

2m was prepared according to the general procedure A for iron(II)-catalyzed biomimetic oxidation. Eluent system for column chromatography: *n*-pentane/EtOAc 95/5 – 80/20 (v/v). Isolated yield: 96% (30.0 mg).

^{1}H NMR (400 MHz, CDCl$_3$) δ ppm 9.35 (s, 1H), 8.25 – 8.20 (m, 2H), 8.19 – 8.10 (m, 2H), 7.84 – 7.71 (m, 2H), 7.63 – 7.49 (m, 3H).

^{13}C NMR (101 MHz, CDCl$_3$) δ ppm 151.9, 143.5, 142.4, 141.7, 136.9, 130.4, 130.3, 129.7, 129.6, 129.3, 129.3, 127.7. The spectral data are consistent with those reported in the literature[6]

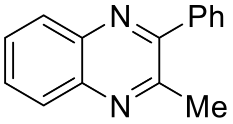

2-Methyl-3-phenylquinoxaline (**2n**)

2n was prepared according to the general procedure A for iron(II)-catalyzed biomimetic oxidation. Eluent system for column chromatography: *n*-pentane/EtOAc 98/3 – 90/10 (v/v). Isolated yield: 95% (31.4 mg).

^{1}H NMR (400 MHz, CDCl$_3$) δ ppm 8.16 – 8.09 (m, 1H), 8.08 – 8.03 (m, 1H), 7.78 – 7.68 (m, 2H), 7.68 – 7.61 (m, 2H), 7.57 – 7.47 (m, 3H), 2.78 (s, 3H).

^{13}C NMR (101 MHz, CDCl$_3$) δ ppm 155.1, 152.7, 141.4, 141.1, 139.2, 129.9, 129.4 (x2), 129.1, 129.1, 128.7, 128.4, 24.5. The spectral data are consistent with those reported in the literature.[7]

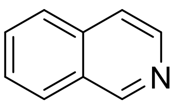

Isoquinoline (4a)

4a was prepared according to the general procedure A for iron(II)-catalyzed biomimetic oxidation. Eluent system for column chromatography: *n*-pentane/EtOAc 98/2 – 90/10 (v/v). Isolated yield: 56% (11.0 mg).

^{1}H NMR (400 MHz, CDCl$_3$) δ ppm 9.26 (s, 1H), 8.53 (d, *J* = 5.7 Hz, 1H), 7.95 (dt, *J* = 8.2, 1.0 Hz, 1H), 7.81 (dd, *J* = 8.3, 1.0 Hz, 1H), 7.68 (ddd, *J* = 8.2, 6.9, 1.3 Hz, 1H), 7.63 (dt, *J* = 5.8, 1.0 Hz, 1H), 7.63 – 7.55 (m, 1H).

^{13}C NMR (101 MHz, CDCl$_3$) δ ppm 152.6, 143.1, 135.8, 130.4, 128.7, 127.6, 127.3, 126.5, 120.5. Spectral data is consistent with data reported in the literature. [2]

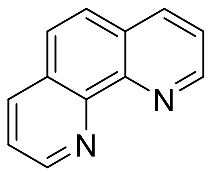

1,10-phenanthroline (4b)

4b was prepared according to the general procedure A for iron(II)-catalyzed biomimetic oxidation using a 25 mL Schenck tube with the side outlet open to air. Eluent system for column chromatography: MeOH/$CHCl_3$ 20/1 (V/V). Isolated yield: 52% (14.0 mg).

^{1}H NMR (400 MHz, $CDCl_3$) δ ppm 9.20 (dd, *J* = 4.3, 1.7 Hz, 2H), 8.27 (dd, *J* = 8.1, 1.8 Hz, 2H), 7.81 (s, 2H), 7.65 (dd, *J* = 8.1, 4.3 Hz, 2H).

^{13}C NMR (100 MHz, $CDCl_3$) δ ppm 150.3, 146.3, 136.0, 128.6, 126.5, 123.1. Spectral data is consistent with data reported in the literature. [2]

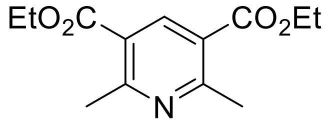

Diethyl 2,6-dimethylpyridine-3,5-dicarboxylate (4c)

4c was prepared according to the general procedure A for iron(II)-catalyzed biomimetic oxidation. Eluent system for column chromatography: *n*-pentane/EtOAc 97/3 – 93/7 (v/v). Isolated yield: 98% (37.0 mg).

^{1}H NMR (400 MHz, $CDCl_3$) δ ppm 8.65 (s, 1H), 4.38 (q, *J* = 7.1 Hz, 4H), 2.82 (s, 6H), 1.39 (t, *J* = 7.1 Hz, 6H).

^{13}C NMR (101 MHz, $CDCl_3$) δ ppm 166.1, 162.3, 141.0, 123.2, 61.5, 25.1, 14.4. Spectral data is consistent with data reported in the literature.[8]

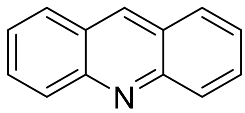

Acridine (4d)

4d was prepared according to the general procedure B for iron(II)-catalyzed biomimetic oxidation using a 25 mL Schenck tube with the side outlet open to air. Eluent system for column chromatography: *n*-pentane/EtOAc 95/5 – 90/10 (v/v). Isolated yield: 99% (26.6 mg).

^{1}**H NMR** (400 MHz, $CDCl_3$) δ ppm 8.77 (s, 1H), 8.25 (dq, *J* = 8.8, 0.9 Hz, 2H), 8.04 – 7.97 (m, 2H), 7.79 (ddd, *J* = 8.8, 6.6, 1.4 Hz, 2H), 7.54 (ddd, *J* = 7.8, 6.6, 1.1 Hz, 2H).

^{13}**C NMR** (100 MHz, $CDCl_3$) δ ppm 149.1, 136.0, 130.3, 129.4, 128.2, 126.6, 125.7.

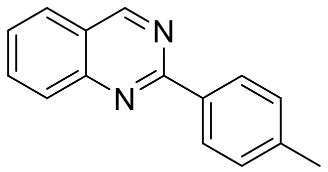

2-(*p*-Tolyl)quinazoline (4e)

4e was prepared according to the general procedure A for iron(II)-catalyzed biomimetic oxidation. Eluent system for column chromatography: *n*-pentane/EtOAc 95/5 – 90/10 (v/v). Isolated yield: 96% (31.8 mg).

^{1}**H NMR (400 MHz, $CDCl_3$)** δ ppm 9.45 (d, *J* = 0.9 Hz, 1H), 8.51 (d, *J* = 8.3 Hz, 2H), 8.07 (dd, *J* = 8.4, 1.0 Hz, 1H), 7.95 – 7.84 (m, 2H), 7.59 (ddd, *J* = 8.1, 7.0, 1.1 Hz, 1H), 7.35 (d, *J* = 8.1 Hz, 2H), 2.45 (s, 3H).

^{13}**C NMR (101 MHz, $CDCl_3$)** δ ppm 161.3, 160.6, 150.9, 141.0, 135.5, 134.2, 129.6, 128.7, 128.7, 127.3, 127.2, 123.7, 21.7. Spectral data is consistent with data reported in the literature.[9]

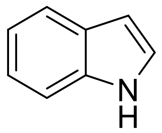

Indole (6a)

6a was prepared according to the general procedure B for iron(II)-catalyzed biomimetic oxidation. Eluent system for column chromatography: *n*-pentane/EtOAc 99/1 – 97/3 (v/v). Isolated yield: 86% (15.1 mg).

^{1}**H NMR (400 MHz, $CDCl_3$)** δ ppm 8.09 (br s, 1H), 7.72 (dq, *J* = 7.8, 1.0 Hz, 1H), 7.42 (dq, *J* = 8.1, 1.0 Hz, 1H), 7.31 – 7.11 (m, 3H), 6.61 (ddd, *J* = 3.2, 2.1, 1.0 Hz, 1H).

^{13}**C NMR (101 MHz, $CDCl_3$)** δ ppm 135.9, 128.0, 124.3, 122.10, 120.9, 119.9, 111.1, 102.7. Spectral data is consistent with data reported in the literature.[4]

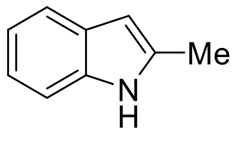

2-Methylindole (6b)

6b was prepared according to the general procedure B for iron(II)-catalyzed biomimetic oxidation. Eluent system for column chromatography: *n*-pentane/EtOAc 99/1 – 98/2 (v/v). Isolated yield: 72% (14.2 mg).

^{1}H NMR (400 MHz, $CDCl_3$) δ 7.80 (br s, 1H), 7.59 – 7.46 (m, 1H), 7.29 (dq, *J* = 8.1, 0.9 Hz, 1H), 7.17 – 6.98 (m, 2H), 6.24 (dt, *J* = 2.0, 1.0 Hz, 1H), 2.45 (d, *J* = 1.0 Hz, 2H).

^{13}C NMR (101 MHz, $CDCl_3$) δ ppm 136.2, 135.2, 129.2, 121.1, 119.7, 110.3, 100.5, 13.8. Spectral data is consistent with data reported in the literature.[10]

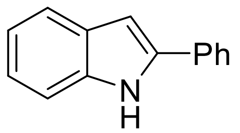

2-Phenylindole (6c)

6c was prepared according to the general procedure B for iron(II)-catalyzed biomimetic oxidation. Eluent system for column chromatography: *n*-pentane/EtOAc 98/2 – 95/5 (v/v). Isolated yield: 96% (28.0 mg).

^{1}H NMR (400 MHz, $CDCl_3$) δ ppm 8.33 (br s, 1H), 7.77 – 7.60 (m, 3H), 7.44 (dt, *J* = 13.0, 7.8 Hz, 3H), 7.38 – 7.29 (m, 1H), 7.23 – 7.15 (m, 1H), 7.12 (d, *J* = 7.4 Hz, 1H), 6.84 (d, *J* = 2.1 Hz, 1H).

^{13}C NMR (101 MHz, $CDCl_3$) δ ppm 138.0, 136.9, 132.5, 129.4, 129.2, 127.9, 125.3, 122.5, 120.8, 120.4, 111.0, 100.1. Spectral data is consistent with data reported in the literature.[11]

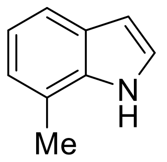

7-Phenylindole (6d)

6d was prepared according to the general procedure B for iron(II)-catalyzed biomimetic oxidation. Eluent system for column chromatography: *n*-pentane/EtOAc 99/1 – 97/3 (v/v). Isolated yield: 97% (19.1 mg).

^{1}H NMR (400 MHz, $CDCl_3$) δ ppm 8.06 (br s, 1H), 7.63 – 7.43 (m, 1H), 7.22 (dd, *J* = 3.2, 2.4 Hz, 1H), 7.15 – 6.96 (m, 2H), 6.59 (dd, *J* = 3.2, 2.1 Hz, 1H), 2.52 (s, 3H).

^{13}C NMR (101 MHz, $CDCl_3$) δ ppm 135.6, 127.5, 123.9, 122.6, 120.3, 120.1, 118.6, 103.3, 16.8.

Spectral data is consistent with data reported in the literature.[12]

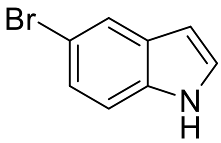

5-Bromoindole (6e)

6e was prepared according to the general procedure B for iron(II)-catalyzed biomimetic oxidation. Eluent system for column chromatography: *n*-pentane/EtOAc 99/1 – 97/3 (v/v). Isolated yield: 82% (24.1 mg).

^{1}H NMR (400 MHz, CDCl$_3$) δ ppm 8.18 (br s, 1H), 7.78 (q, *J* = 1.1 Hz, 1H), 7.28 (d, *J* = 1.4 Hz, 2H), 7.22 (dd, *J* = 3.2, 2.4 Hz, 2H), 6.50 (dd, *J* = 3.3, 2.1 Hz, 1H).

^{13}C NMR (101 MHz, CDCl$_3$) δ ppm 134.5, 129.8, 125.5, 125.0, 123.4, 113.2, 112.5, 102.4. Spectral data is consistent with data reported in the literature. [13]

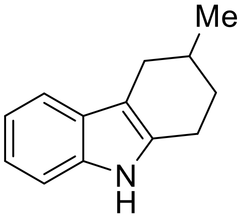

3-Methyl-2,3,4,9-tetrahydro-1H-carbazole (6f)

6f was prepared according to the general procedure B for iron(II)-catalyzed biomimetic oxidation. Eluent system for column chromatography: *n*-pentane/EtOAc 98/2 – 95/5 (v/v). Isolated yield: 95% (26.4 mg).

^{1}H NMR (400 MHz, CDCl$_3$) δ ppm 7.64 (s, 1H), 7.46 (d, *J* = 7.4 Hz, 1H), 7.32 – 7.18 (m, 1H), 7.10 (tt, *J* = 7.8, 6.1 Hz, 2H), 2.89 – 2.82 (m, 1H), 2.79 – 2.69 (m, 2H), 2.36 – 2.21 (m, 1H), 2.02 – 1.88 (m, 2H), 1.66 – 1.42 (m, 1H), 1.15 (d, *J* = 6.5 Hz, 3H).

^{13}C NMR (101 MHz, CDCl$_3$) δ ppm 136.1, 134.0, 127.9, 121.1, 119.2, 117.85, 110.5, 110.3, 31.5, 29.8, 29.5, 23.0, 21.9. Spectral data is consistent with data reported in the literature. [14]

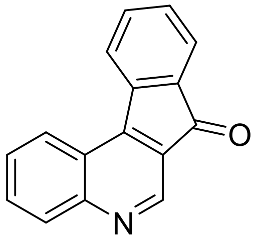

7*H*-Indeno[2,1-*c*]quinolin-7-one (8a)

8a was prepared according to the general procedure A for iron(II)-catalyzed biomimetic oxidation. Eluent system for column chromatography: *n*-pentane/EtOAc 90/10 – 80/20 (v/v). Isolated yield: 69% (24.0 mg).

^{1}H NMR (400 MHz, $CDCl_3$) δ ppm 9.17 (s, 1H), 8.49 (d, *J* = 8.4 Hz, 1H), 8.18 (d, *J* = 8.5 Hz, 1H), 8.13 (d, *J* = 7.5 Hz, 1H), 7.91 – 7.82 (m, 1H), 7.78 (d, *J* = 7.3 Hz, 1H), 7.70 (t, *J* = 7.7 Hz, 1H), 7.63 (t, *J* = 7.5 Hz, 1H), 7.50 (t, *J* = 7.5 Hz, 1H).

^{13}C NMR (101 MHz, $CDCl_3$) δ ppm 193.1, 152.7, 151.2, 144.9, 142.6, 134.7, 134.0, 132.2, 131.3, 131.2, 128.3, 125.1, 124.8, 124.9, 124.6, 123.7. Spectral data is consistent with data reported in the literature. [3]

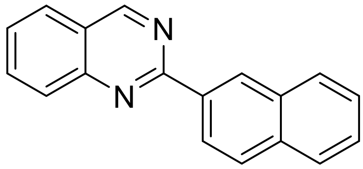

2-(Naphthalen-2-yl)quinazoline (11a)

To a 10 mL vial added 2-(aminomethyl)aniline (18.33 mg, 0.15 mmol) and 2-naphthaldehyde (23.427mg, 0.15 mmol) added in dry MeOH (2 mL) under nitrogen and run for 16 h at RT. After 16 h solvent was removed in vacuo, to the same vial were added **IId** (17.68 mg, 0.03 mmol), TMANO (1.2 mg, 0.015 mmol), **Ia** (8.7 mg, 0.015 mmol) and dry DMSO (2.0 mL). The via was fitted with rubber septum under air and stirred at 90 °C for 36 h. Upon completion, reaction mixture was work-up with Et_2O (20 mL x 3), organic layer wah with brine (20 mL) and dry with Na_2SO_4. Reaction mixture was concentrated in vacuo and the crude product was purified by column chromatography. Eluent system for column chromatography: *n*-pentane/EtOAc 90/10 – 80/20 (v/v). Spectral data is consistent with data reported in the literature.[9] Isolated yield: 78% (30.0 mg).

^{1}H NMR (400 MHz, CDCl$_3$) δ ppm 9.52 (d, *J* = 0.8 Hz, 1H), 9.22 – 9.15 (m, 1H), 8.74 (dd, *J* = 8.6, 1.7 Hz, 1H), 8.19 – 8.13 (m, 1H), 8.09 – 8.03 (m, 1H), 8.00 (d, *J* = 8.7 Hz, 1H), 7.97 – 7.89 (m, 3H), 7.63 (ddd, *J* = 8.0, 6.9, 1.1 Hz, 1H), 7.57 – 7.51 (m, 2H).
^{13}C NMR (101 MHz, CDCl$_3$) δ ppm 161.2, 160.7, 151.1, 135.5, 134.8, 134.3, 133.6, 129.4, 129.1, 128.8, 128.5, 127.9, 127.5, 127.3, 127.3, 126.4, 125.6, 123.8.
The spectral data are consistent with those reported in the literature.[9]

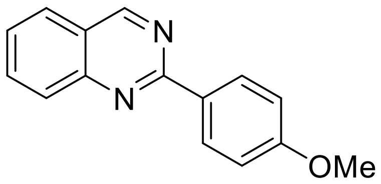

2-(4-Methoxyphenyl)quinazoline (11b)

To a 10 mL vial added 2-(aminomethyl)aniline (18.33 mg, 0.15 mmol) and 4-methoxybenzaldehyde (20.40 mg, 0.15 mmol) added in dry MeOH (2 mL) under nitrogen and run for 16 h at RT. After 16 h, solvent was removed in vacuo, to the same vial were added **IId** (17.68 mg, 0.03 mmol), TMANO (1.2 mg, 0.015 mmol), **Ia** (8.7 mg, 0.015 mmol) and dry DMSO (2.0 mL). The via was fitted with rubber septum under air and stirred at 90 °C for 36 h. Upon completion, reaction mixture was work-up with Et_2O (20 mL x 3), organic layer wah with brine (20 mL) and dry with Na_2SO_4. Reaction mixture was concentrated in vacuo and the crude product was purified by column chromatography. Eluent system for column chromatography: *n*-pentane/EtOAc 90/10 – 80/20 (v/v). Isolated yield: 60% (21.3 mg).
^{1}H NMR (400 MHz, CDCl$_3$) δ ppm 9.42 (d, *J* = 0.8 Hz, 1H), 8.58 (d, *J* = 8.9 Hz, 2H), 8.04 (dd, *J* = 8.4, 1.0 Hz, 1H), 7.93 – 7.83 (m, 2H), 7.57 (ddd, *J* = 8.1, 7.0, 1.1 Hz, 1H), 7.05 (d, *J* = 8.9 Hz, 2H), 3.90 (s, 3H).
^{13}C NMR (101 MHz, CDCl$_3$) δ ppm 162.0, 161.0, 160.6, 151.0, 134.2, 130.9, 130.3, 128.6, 127.3, 126.9, 123.5, 114.13, 55.5. Spectral data is consistent with data reported in the literature. [9]

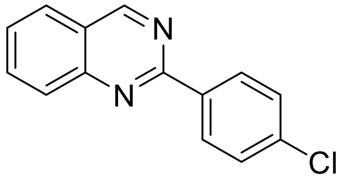

2-(4-Chlorophenyl)quinazoline (11c)

To a 10 mL vial was added 2-(aminomethyl)aniline (18.33 mg, 0.15 mmol) and 4-chlorobenzaldehyde (21.08 mg, 0.15 mmol) added in dry MeOH (2 mL) under nitrogen and run for 16 h at RT. After 16 h, solvent was removed in vacuo, to the same vial were added **IId** (17.68 mg, 0.03 mmol), TMANO (1.2 mg, 0.015 mmol), **Ia** (8.7 mg, 0.015 mmol) and dry DMSO (2.0 mL). The via was fitted with rubber septum under air and stirred at 90 °C for 36 h. Upon completion, reaction mixture was work-up with Et_2O (20 mL x 3), organic layer wah with brine (20 mL) and dry with Na_2SO_4. Reaction mixture was concentrated in vacuo and the crude product was purified by column chromatography. Eluent system for column chromatography: *n*-pentane/EtOAc 90/10 – 85/15 (v/v). Isolated yield: 72% (26.0 mg).

^{1}H NMR (400 MHz, $CDCl_3$) δ ppm 9.45 (d, *J* = 0.8 Hz, 1H), 8.66 – 8.51 (m, 2H), 8.12 – 8.03 (m, 1H), 7.98 – 7.86 (m, 2H), 7.63 (ddd, *J* = 8.1, 7.0, 1.1 Hz, 1H), 7.54 – 7.45 (m, 2H).

^{13}C NMR (101 MHz, $CDCl_3$) δ ppm160.7, 160.2, 150.9, 137.0, 136.7, 134.4, 130.1, 129.0, 128.8, 127.6, 127.3, 123.8. Spectral data is consistent with data reported in the literature. [9]

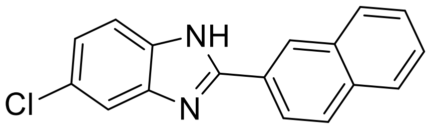

5-Chloro-2-(naphthalen-2-yl)-1H-benzo[*d*]imidazole (14a)

To a 10 mL vial was added 4-chlorobenzene-1,2-diamine (21.39 mg, 0.15 mmol) and 2-naphthaldehyde (21.08 mg, 0.15 mmol) added in dry MeOH (2 mL) under nitrogen and run for 16 h at RT. After 16 h, to the same vial were added **IId** (8.84 mg, 0.015 mmol), TMANO (0.6 mg, 0.0075mmol), **Ia** (4.4 mg, 0.0075 mmol). The via was fitted with rubber septum under air and stirred at 90 °C for 16 h. Upon completion, reaction mixture was concentrated in vacuo and the crude product was purified by column chromatography. Eluent system for column chromatography: *n*-pentane/EtOAc 90/10 – 80/20 (v/v). Isolated yield: 84% (35.0 mg).

^{1}H NMR (400 MHz, DMSO-D_6) δ ppm 7.72 (d, *J* = 1.8 Hz, 1H), 7.32 (dd, *J* = 8.6, 1.8 Hz, 1H), 7.16 (dd, *J* = 10.8, 7.3 Hz, 2H), 7.08 (dt, *J* = 7.6, 3.8 Hz, 1H), 6.89 – 6.60 (m, 4H), 6.43 (dd, *J* = 8.5, 2.0 Hz, 1H).

^{13}C NMR (101 MHz, DMSO-D_6) δ ppm 145.2, 126.2, 125.1, 120.5 (x 2), 120.2 (x 2), 119.4 (x 2), 119.1 (x 2), 118.6 (x 2), 118.3, 118.2, 115.2, 114.9. Spectral data is consistent with data reported in the literature.[15]

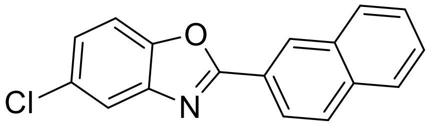

5-Chloro-2-(naphthalen-2-yl)benzo[d]oxazole (14b)

To a 10 mL vial was added 2-amino-4-chlorophenol (21.54 mg, 0.15 mmol) and 2-naphthaldehyde (21.08 mg, 0.15 mmol) added in dry MeOH (2 mL) under nitrogen and run for 16 h at RT. After 16 h, to the same vial were added **IId** (8.84 mg, 0.015 mmol), TMANO (0.6 mg, 0.0075mmol), **Ia** (4.4 mg, 0.0075 mmol). The via was fitted with rubber septum under air and stirred at 80 °C for 16 h. Upon completion, reaction mixture was concentrated in vacuo and the crude product was purified by column chromatography. Eluent system for column chromatography: *n*-pentane/EtOAc 99/1 – 97/3 (v/v). Isolated yield: 94% (39.4 mg).

^{1}H NMR (400 MHz, $CDCl_3$) δ ppm 8.74 (d, *J* = 1.8 Hz, 1H), 8.26 (dd, *J* = 8.6, 1.7 Hz, 1H), 7.97 (dd, *J* = 8.7, 5.0 Hz, 2H), 7.90 – 7.85 (m, 1H), 7.76 (d, *J* = 2.1 Hz, 1H), 7.63 – 7.53 (m, 2H), 7.51 (d, *J* = 8.6 Hz, 1H), 7.33 (dd, *J* = 8.6, 2.1 Hz, 1H).

^{13}C NMR (101 MHz, $CDCl_3$) δ ppm 164.6, 149.6, 143.5, 135.0, 133.0, 130.2, 129.1, 129.0, 128.6, 128.2, 128.1, 127.14, 125.5, 124.0, 124.0, 120.1, 111.4. Spectral data is consistent with data reported in the literature. [16]

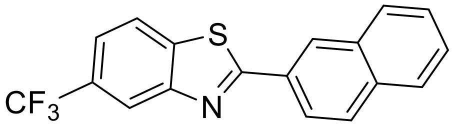

2-(Naphthalen-2-yl)-5-(trifluoromethyl)benzo[*d*]thiazole (14c)

To a 10 mL vial was added 2-amino-4-chlorobenzenethiol (23.94 mg, 0.15 mmol) and 2-naphthaldehyde (21.08 mg, 0.15 mmol) added in dry MeOH (2 mL) under nitrogen and run for 16 h at RT. After 16 h, to the same vial was added **IId** (8.84 mg, 0.015 mmol), TMANO (0.6 mg, 0.0075mmol), **Ia** (4.4 mg, 0.0075 mmol). The via was fitted with rubber septum under air and stirred at 80 °C for 16 h. Upon completion, reaction mixture was concentrated in vacuo and the crude product was purified by column chromatography. Eluent system for column chromatography: *n*-pentane/EtOAc 99/1 – 97/3 (v/v). Isolated yield: 97% ()48.0 mg.

^{1}H NMR (400 MHz, $CDCl_3$) δ ppm 8.87 – 8.73 (m, 1H), 8.63 (d, *J* = 5.4 Hz, 1H), 8.44 (dd, *J* = 8.6, 2.1 Hz, 1H), 8.29 (dd, *J* = 8.2, 3.5 Hz, 1H), 8.27 – 8.19 (m, 2H), 8.18 – 8.09 (m, 1H), 8.00 – 7.74 (m, 3H).

^{13}C NMR (101 MHz, $CDCl_3$) δ ppm 170.5, 153.7, 138.5, 134.9, 133.1, 130.3, 129.1 (q, *J* = 32.8 Hz), 128.9, 128.1, 127.9, 127.1, 125.6, 124.2 (q, *J* = 272.2 Hz), 124.1, 122.9, 122.4, 121.6, 120.23 (*q*, *J* = 4.5 Hz).

Spectral data is consistent with data reported in the literature. [14)

NMR Spectra of synthesize compounds

^{1}H NMR of **2a** (400 MHz, $CDCl_3$)

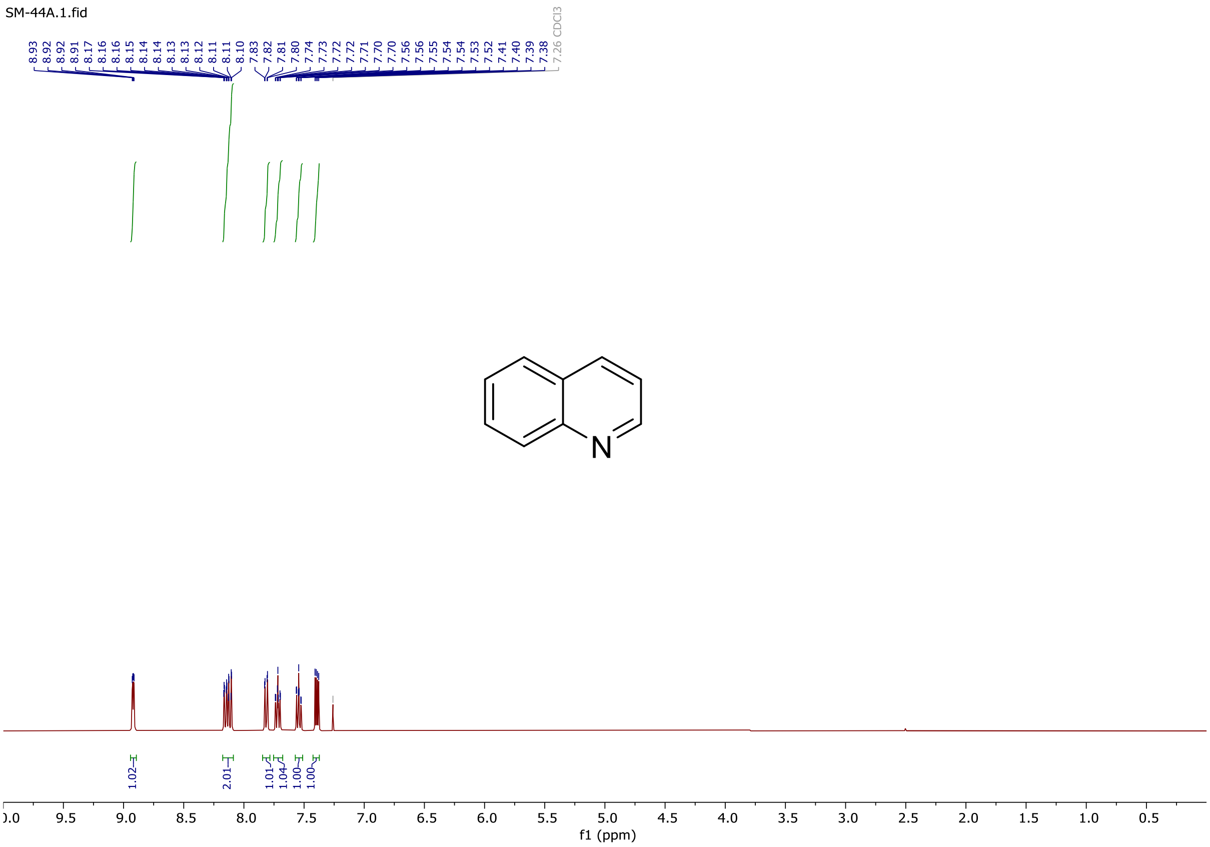

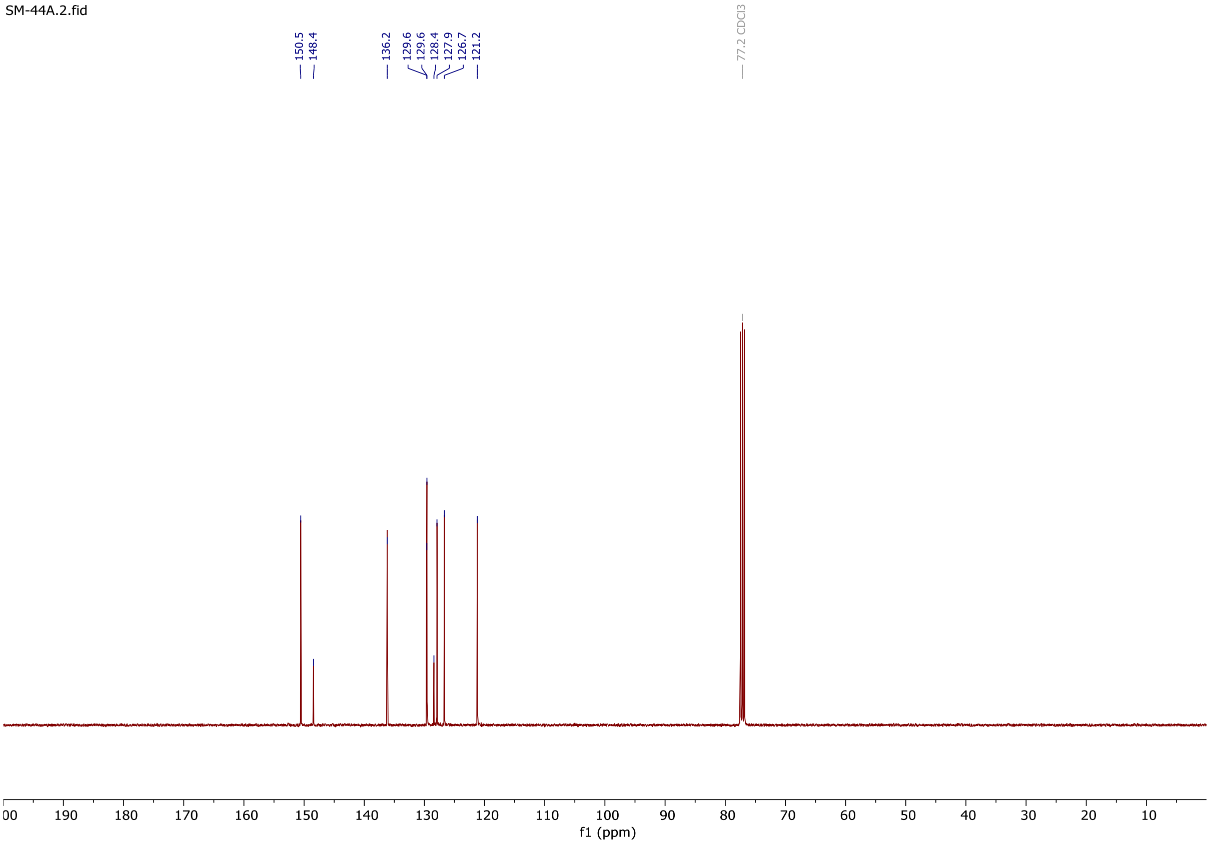

^{1}H NMR of **2b** (400 MHz, $CDCl_3$)

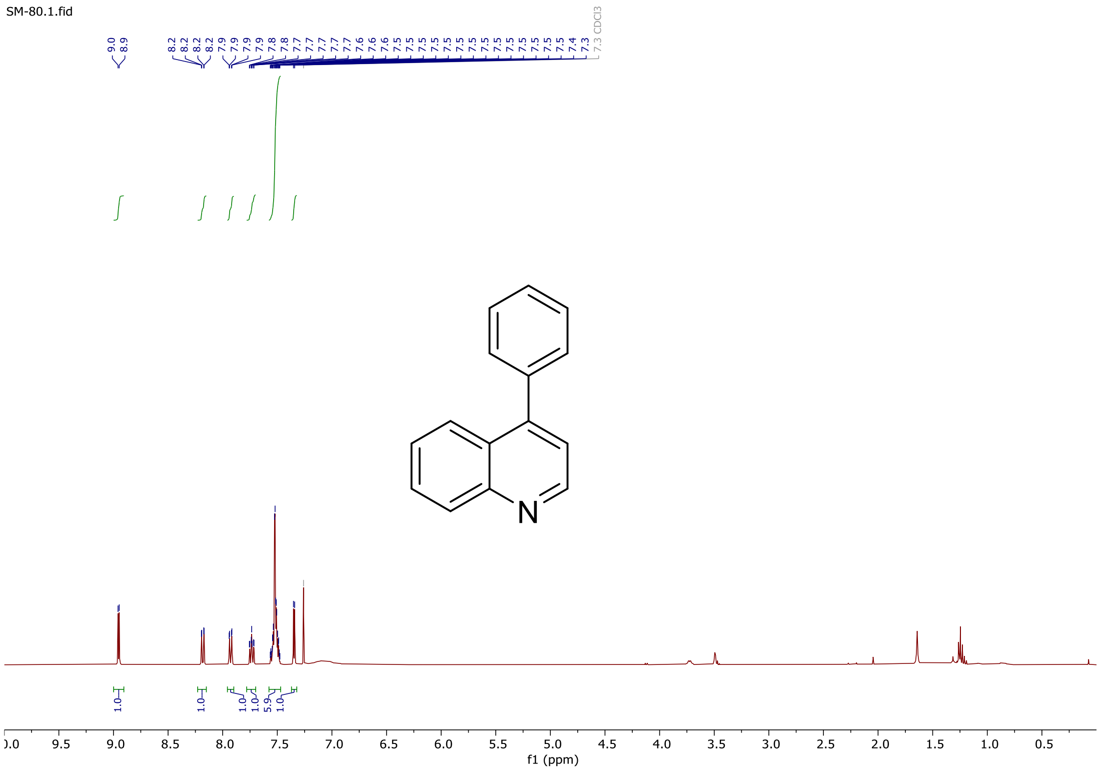

^{13}C NMR of **2b** (101 MHz, $CDCl_3$)

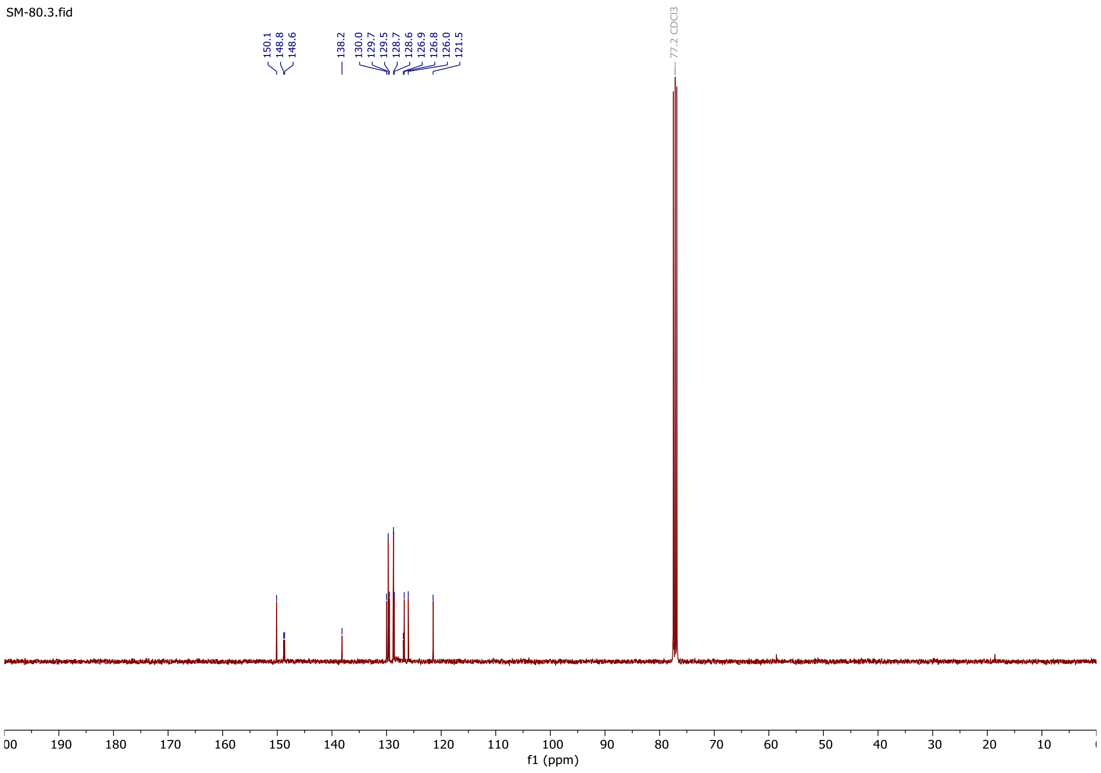

^{1}H NMR of **2c** (400 MHz, $CDCl_3$)

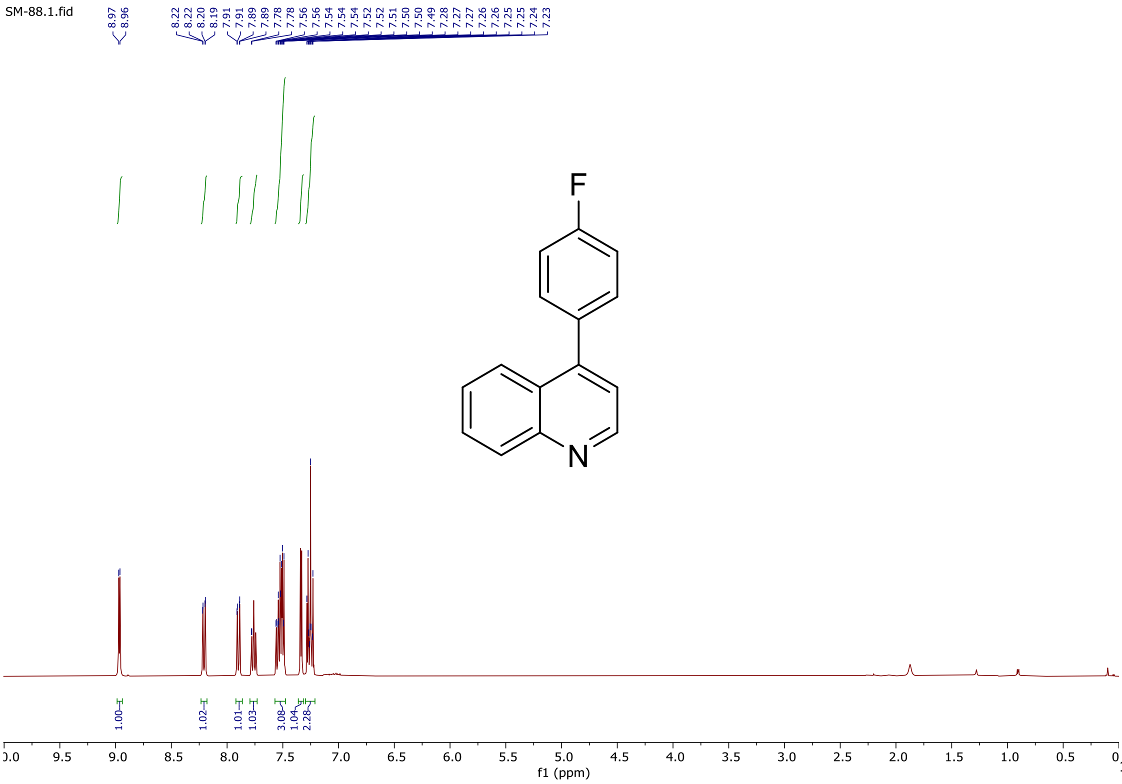

^{13}C NMR of **2c** (101 MHz, $CDCl_3$)

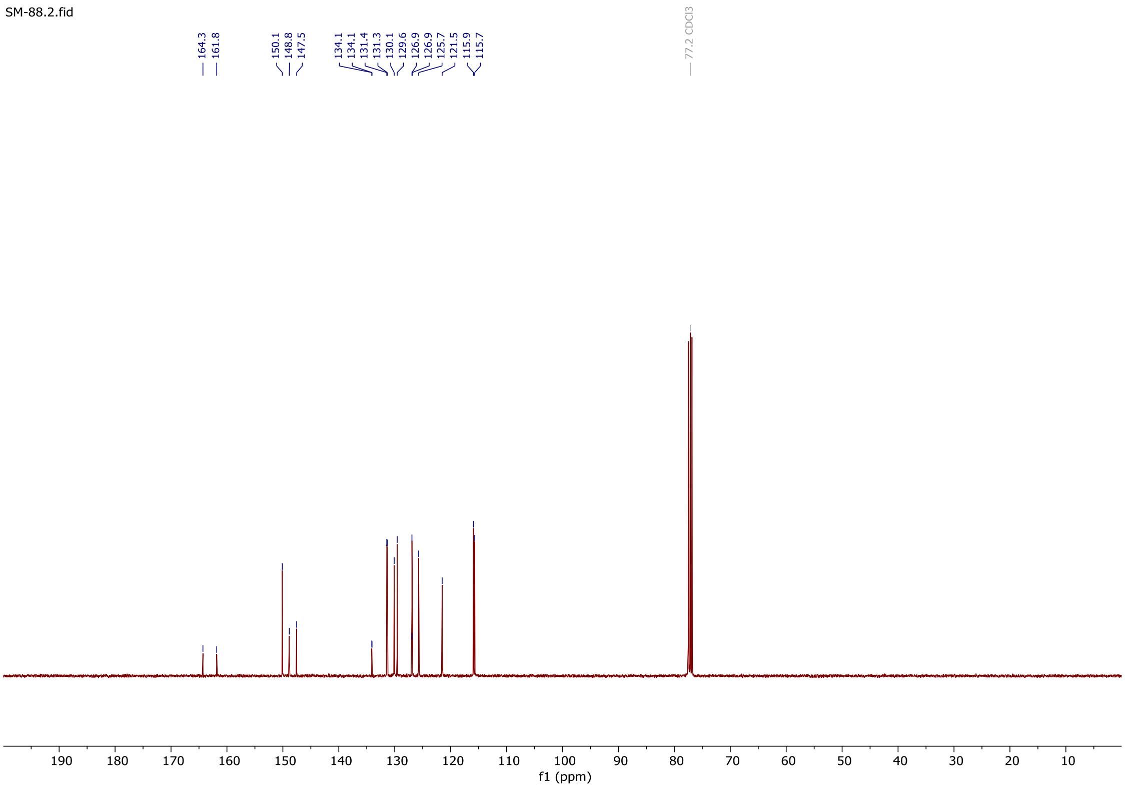

^{1}H NMR of **2d** (400 MHz, $CDCl_3$)

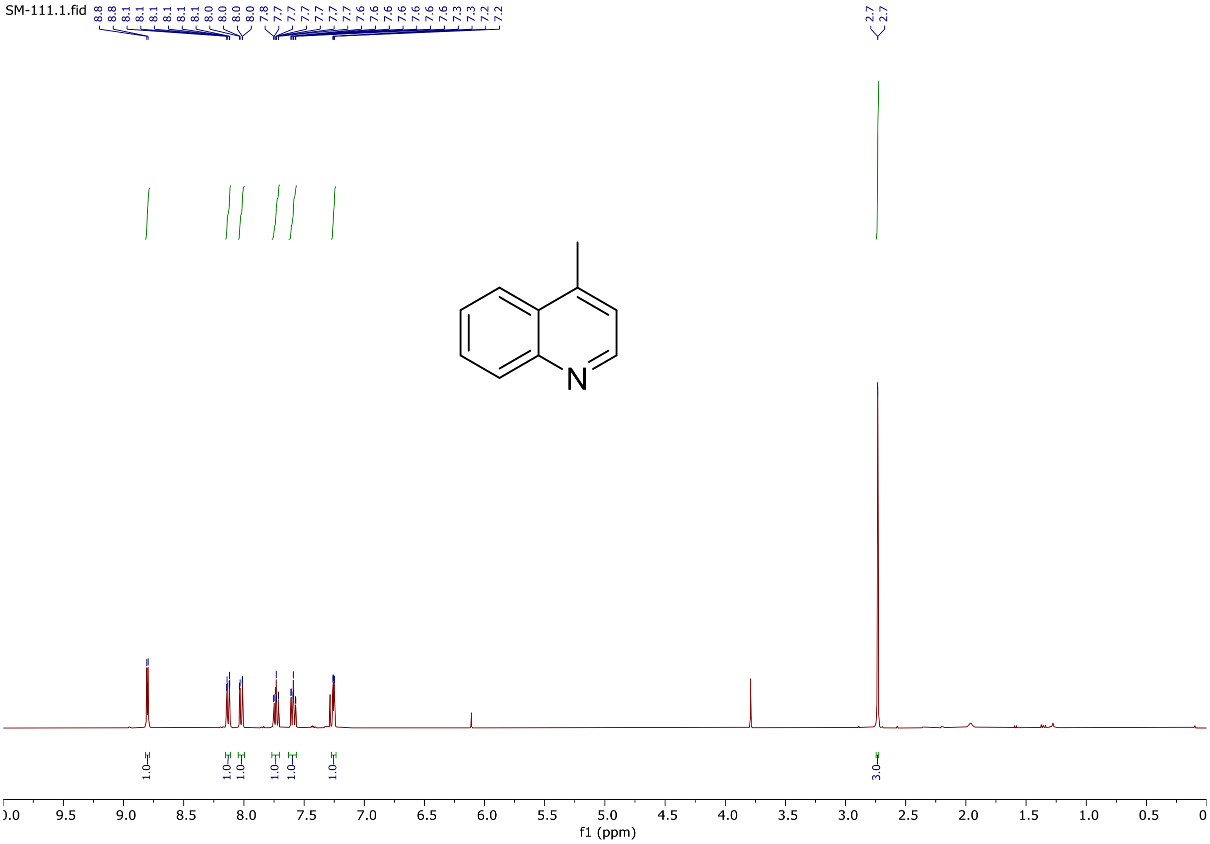

^{13}C NMR of **2d** (101 MHz, $CDCl_3$)

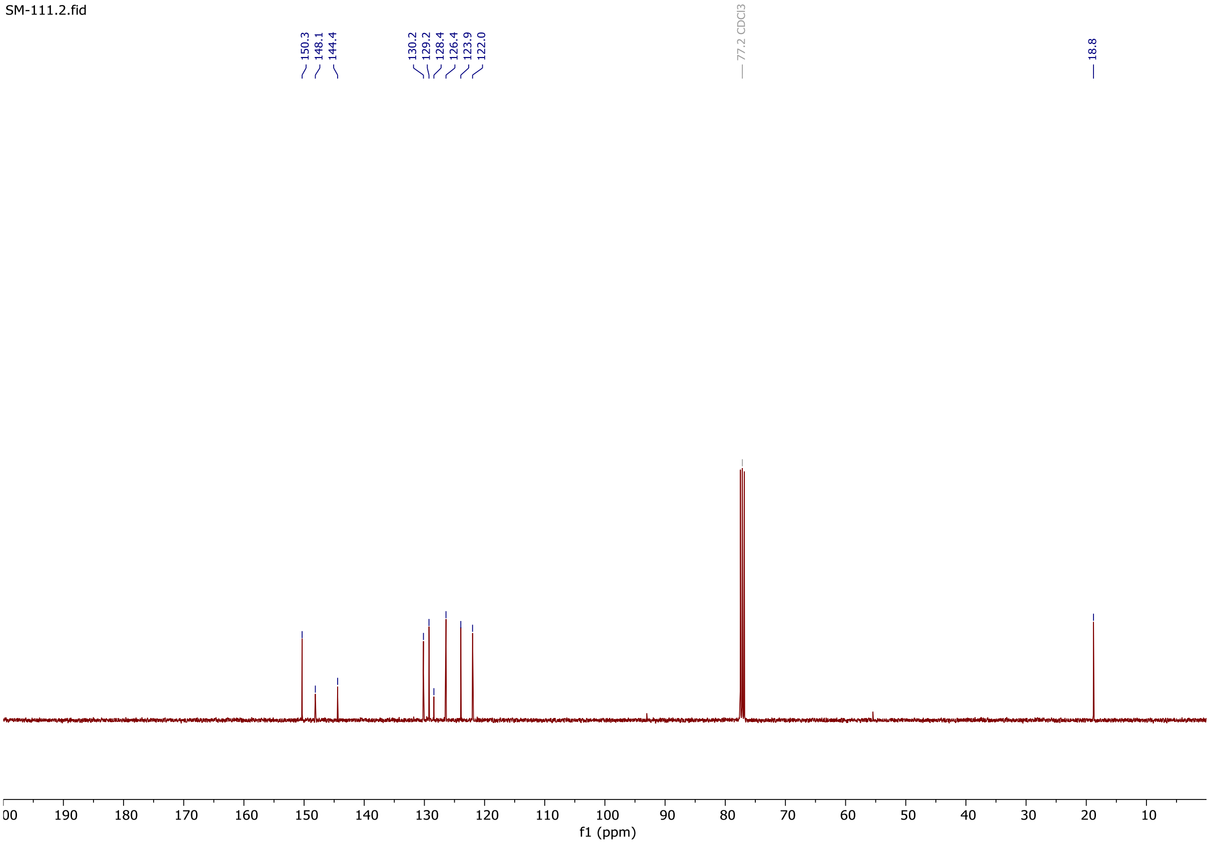

^{1}H NMR of **2e** (400 MHz, $CDCl_3$)

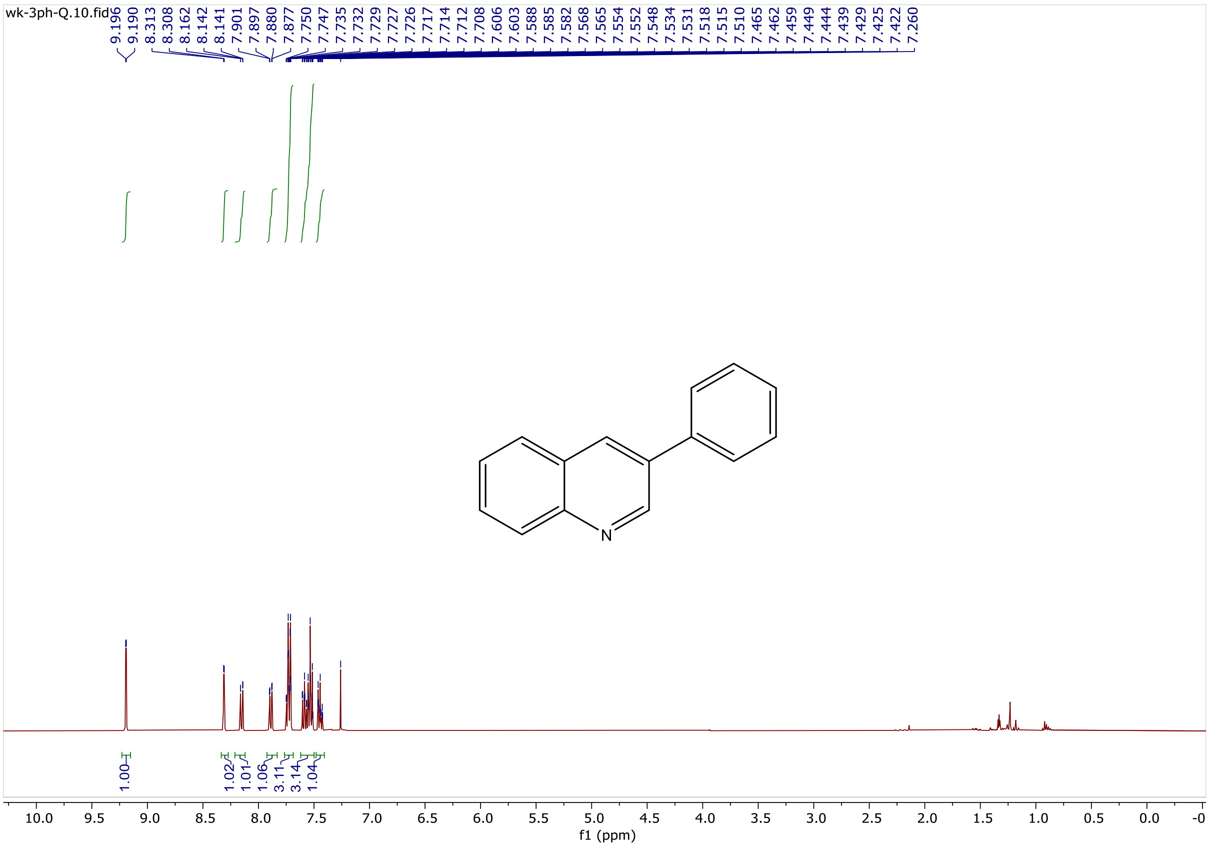

^{13}C NMR of **2e** (101 MHz, $CDCl_3$)

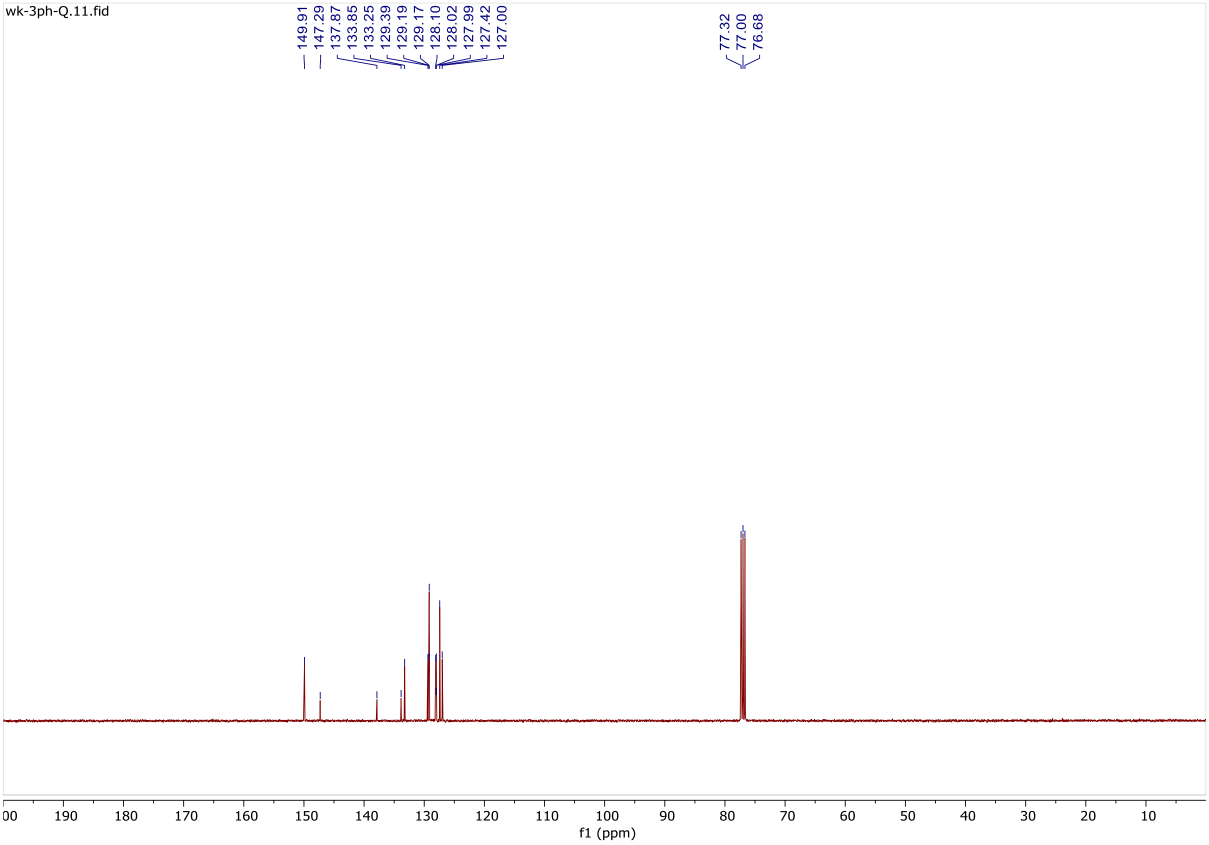

^{1}H NMR of **2f** (400 MHz, $CDCl_3$)

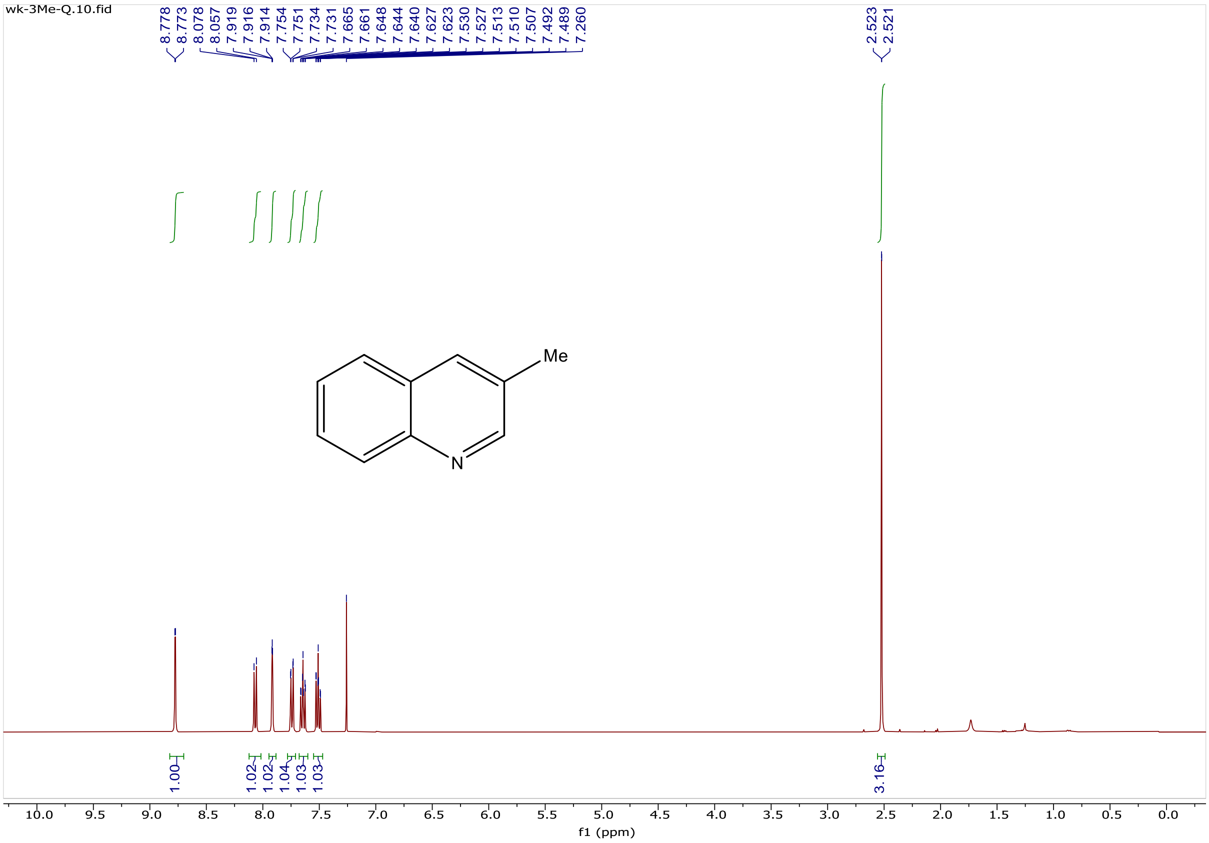

^{13}C NMR of **2f** (101 MHz, $CDCl_3$)

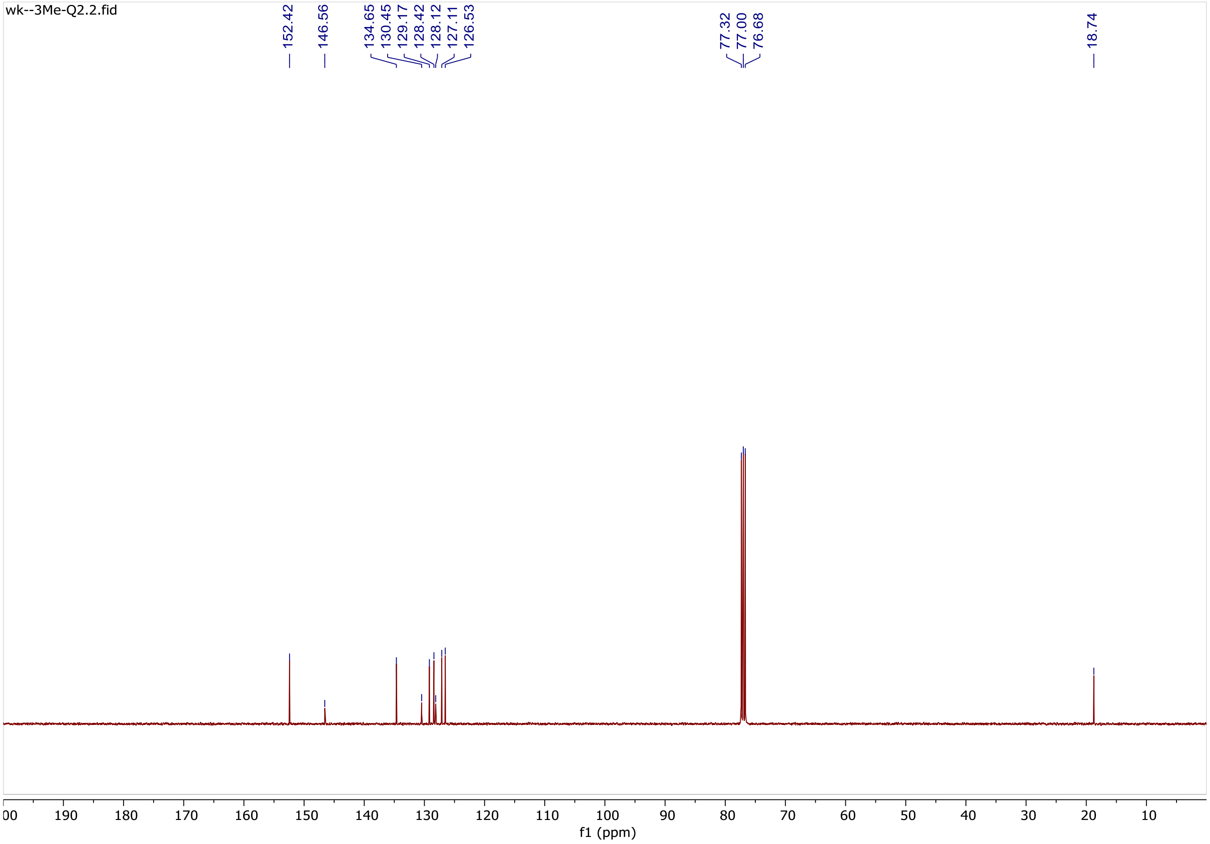

^{1}H NMR of **2g** (400 MHz, $CDCl_3$)

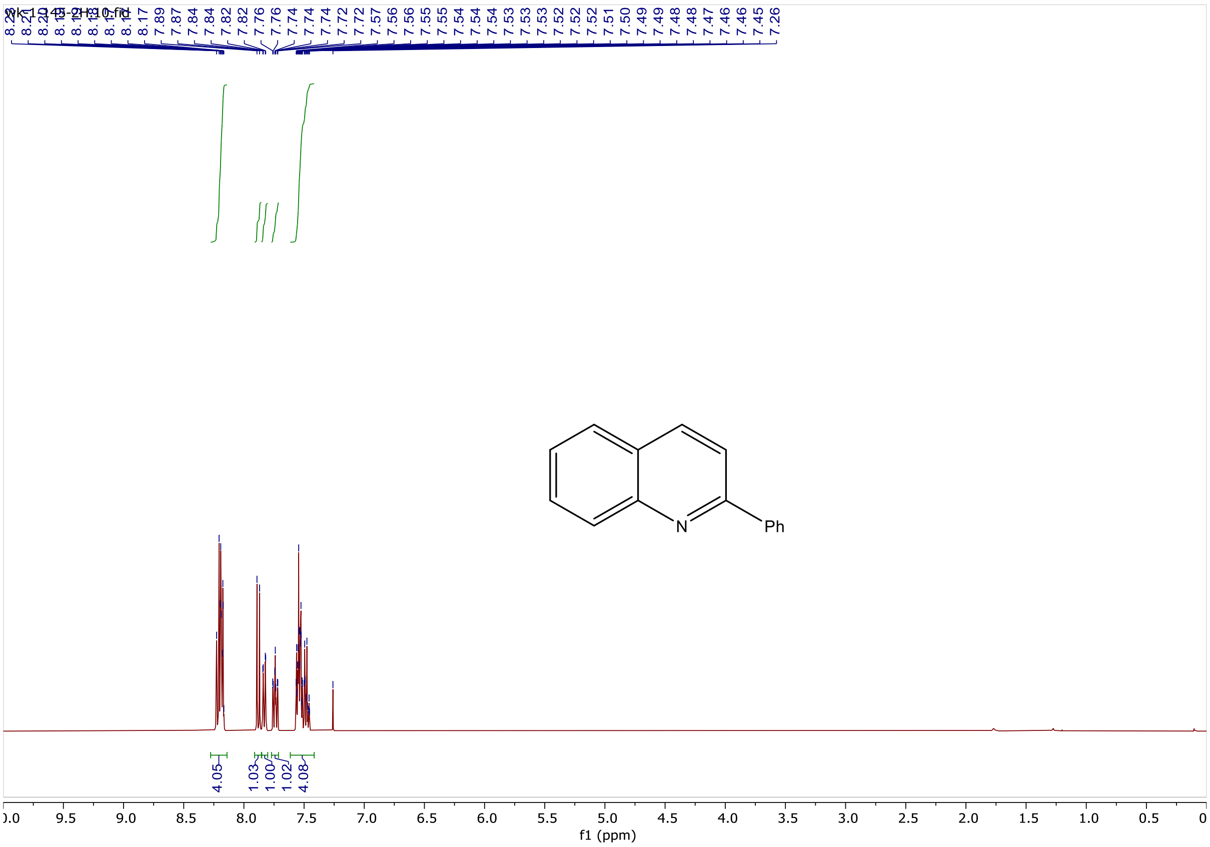

^{13}C NMR of **2g** (101 MHz, $CDCl_3$)

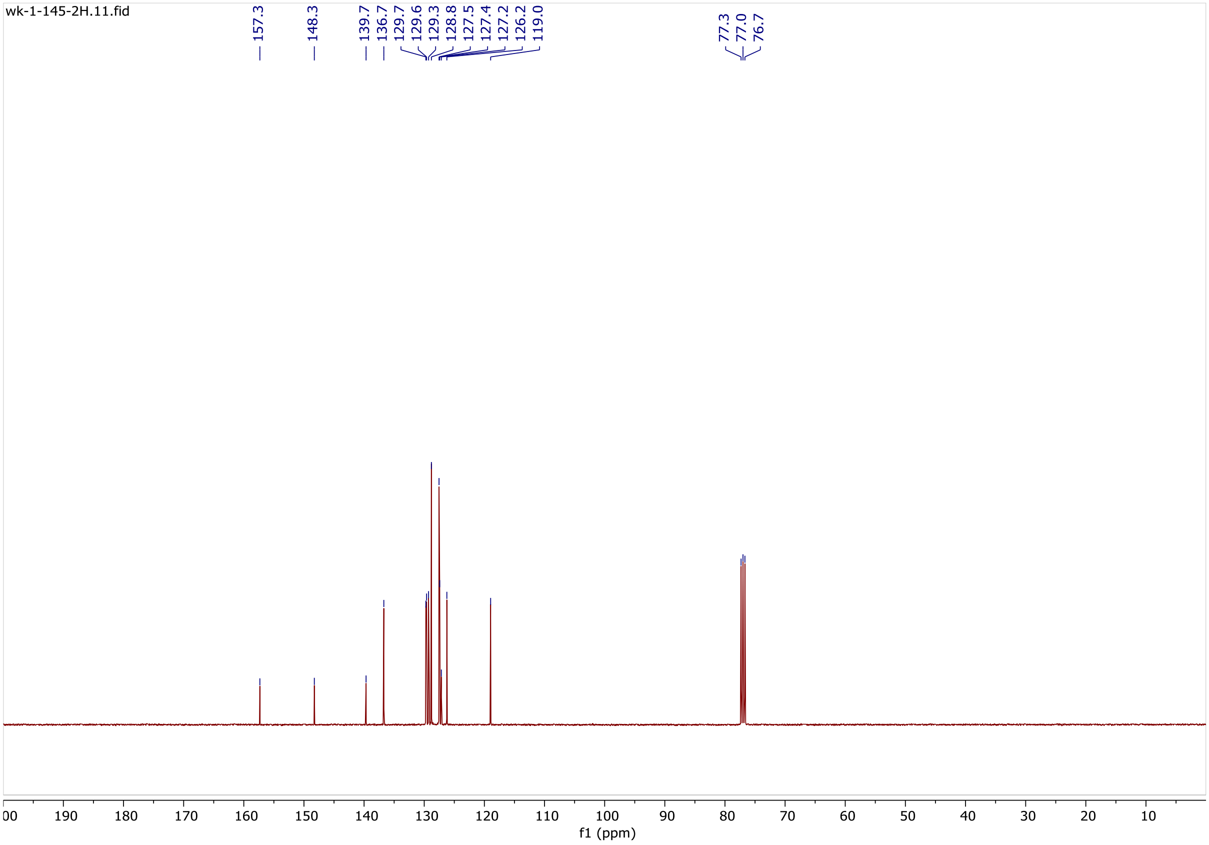

^{1}H NMR of **2h** (400 MHz, CDCl₃)

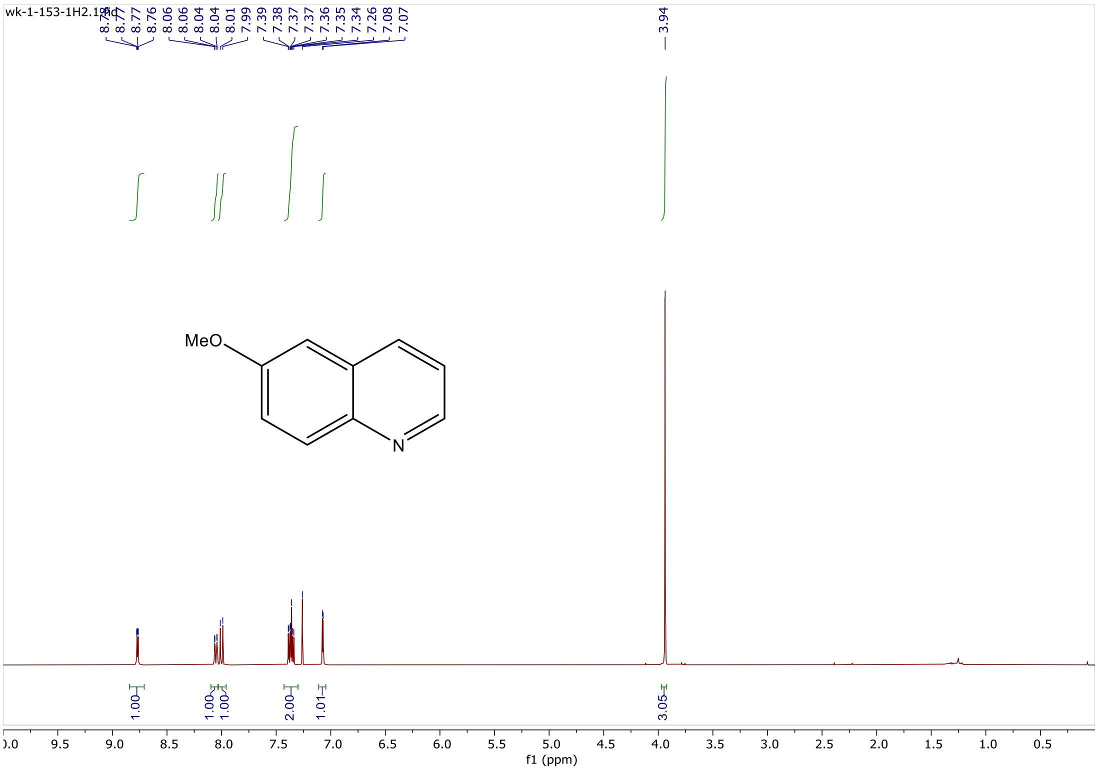

^{13}C NMR of **2g** (101 MHz, CDCl₃)

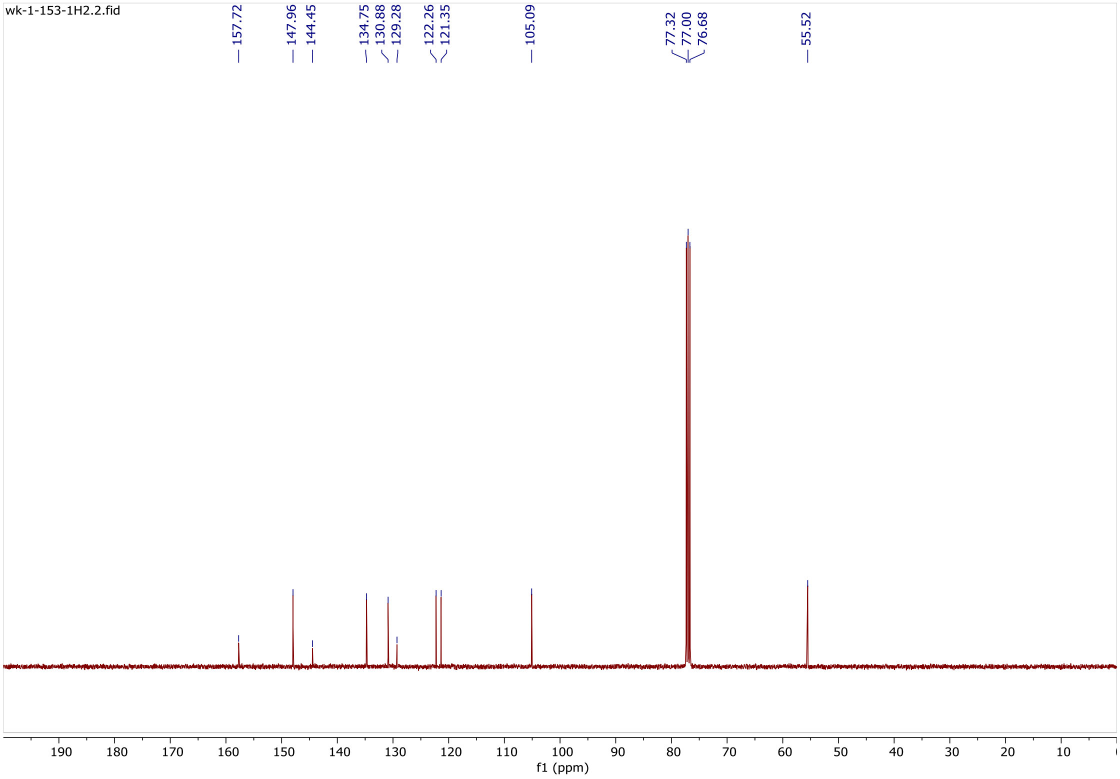

^{1}H NMR of **2i** (400 MHz, $CDCl_3$)

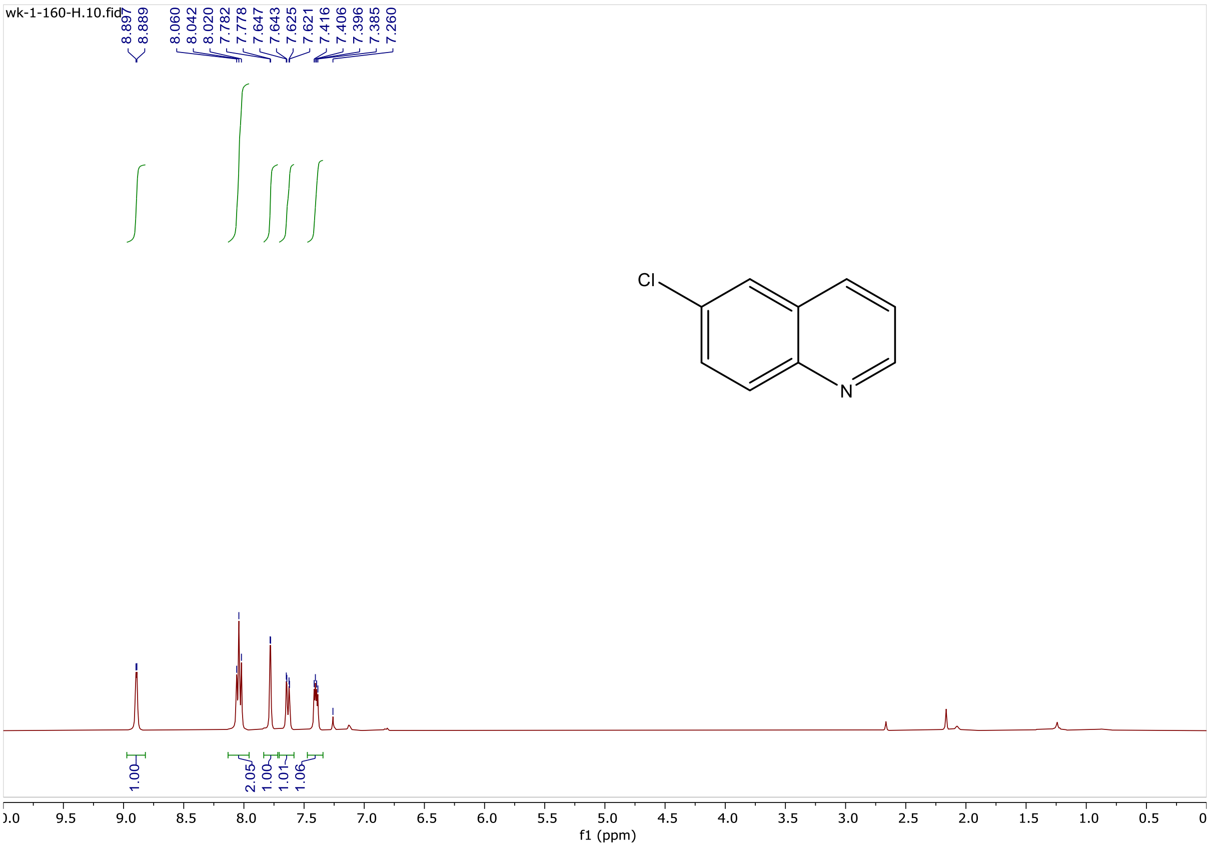

^{13}C NMR of **2i** (101 MHz, $CDCl_3$)

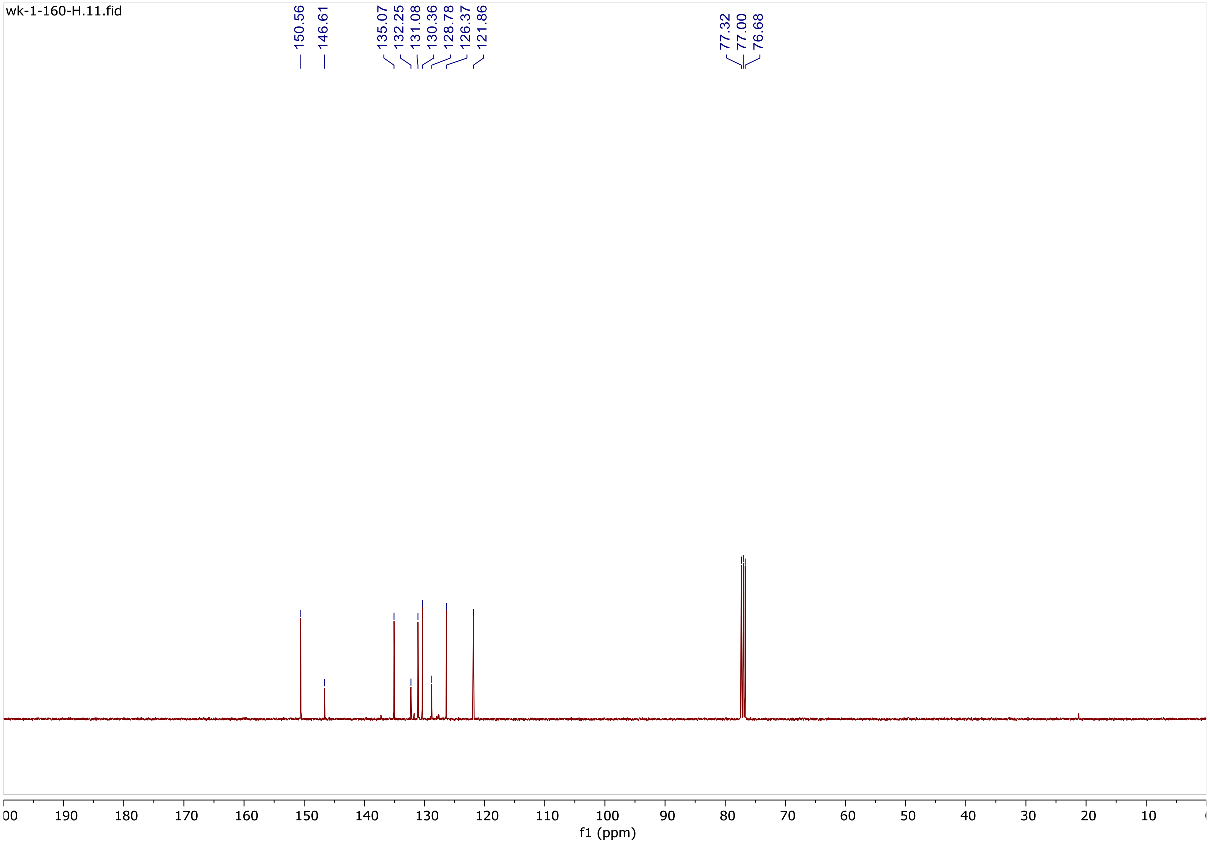

^{1}H NMR of **2j** (400 MHz, $CDCl_3$)

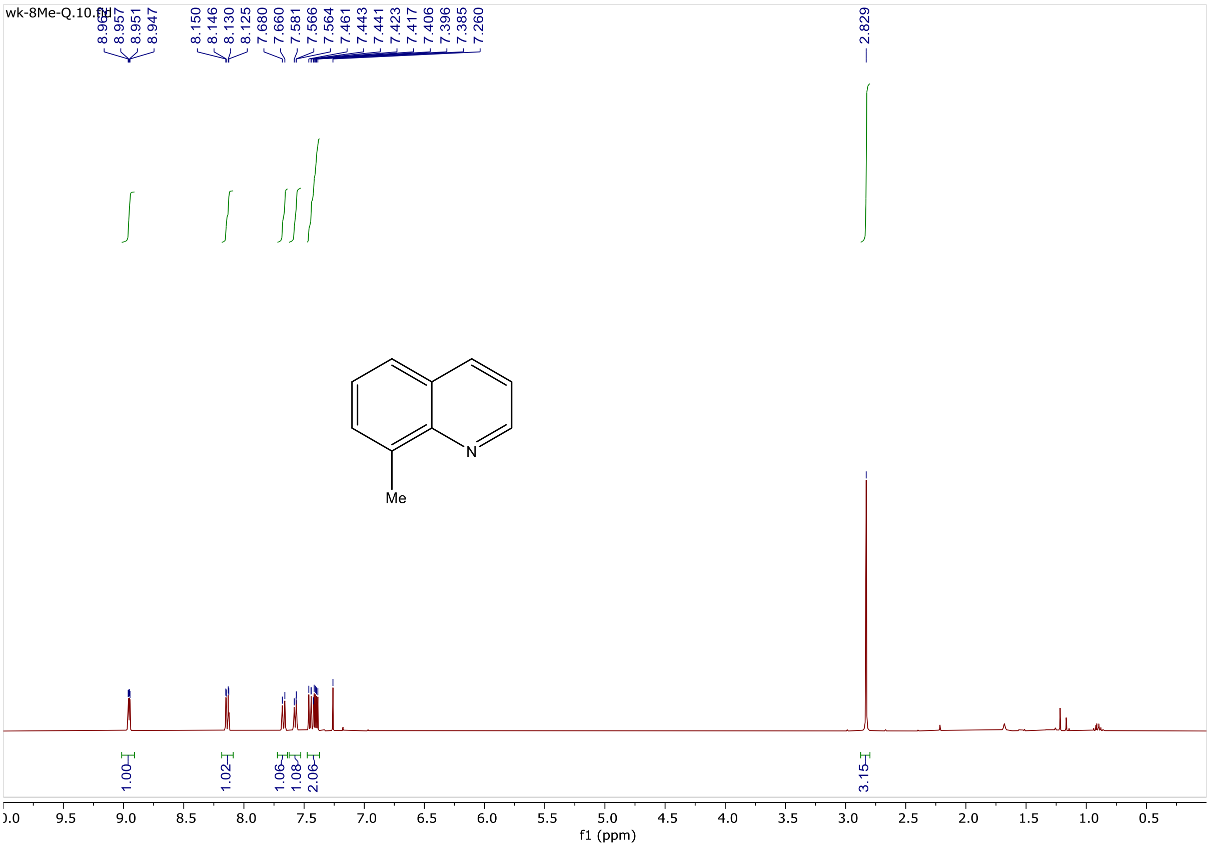

^{13}C NMR of **2j** (101 MHz, $CDCl_3$)

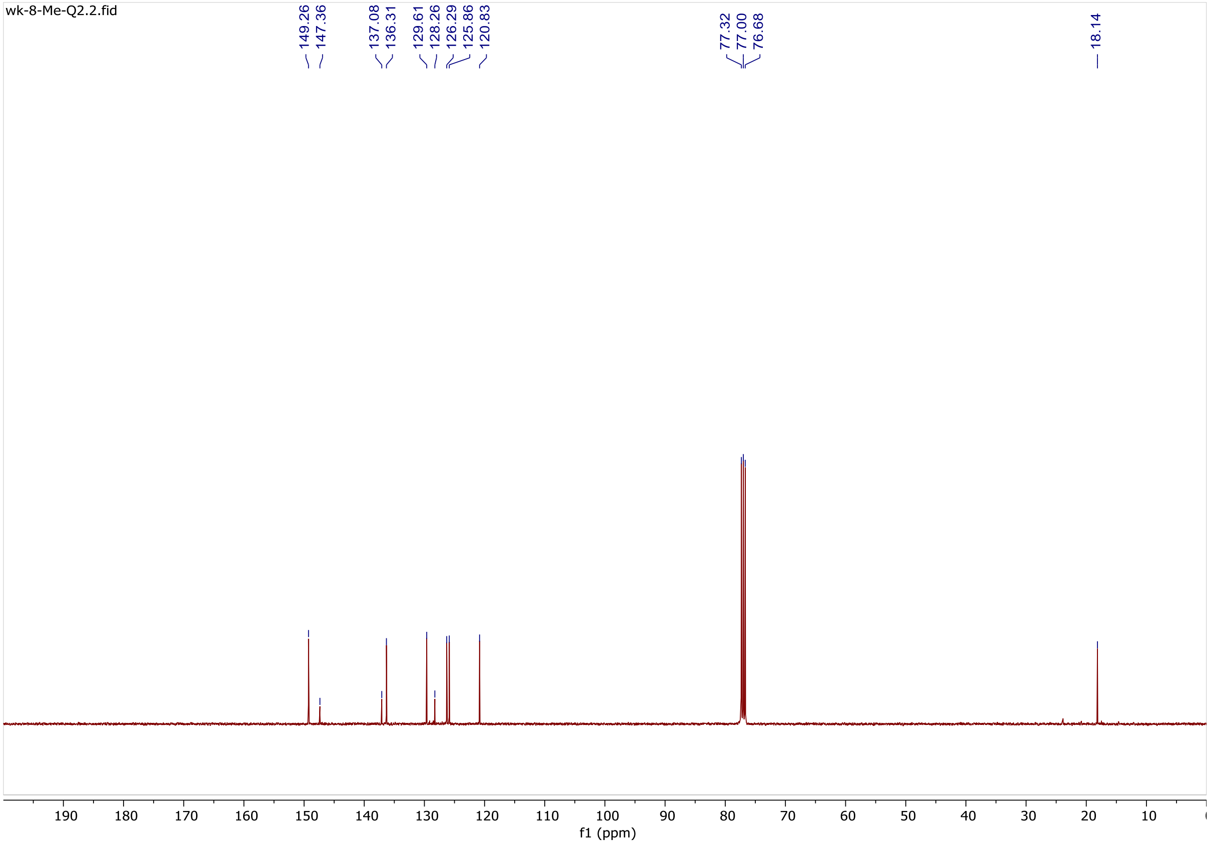

^{1}H NMR of **2k** (400 MHz, $CDCl_3$)

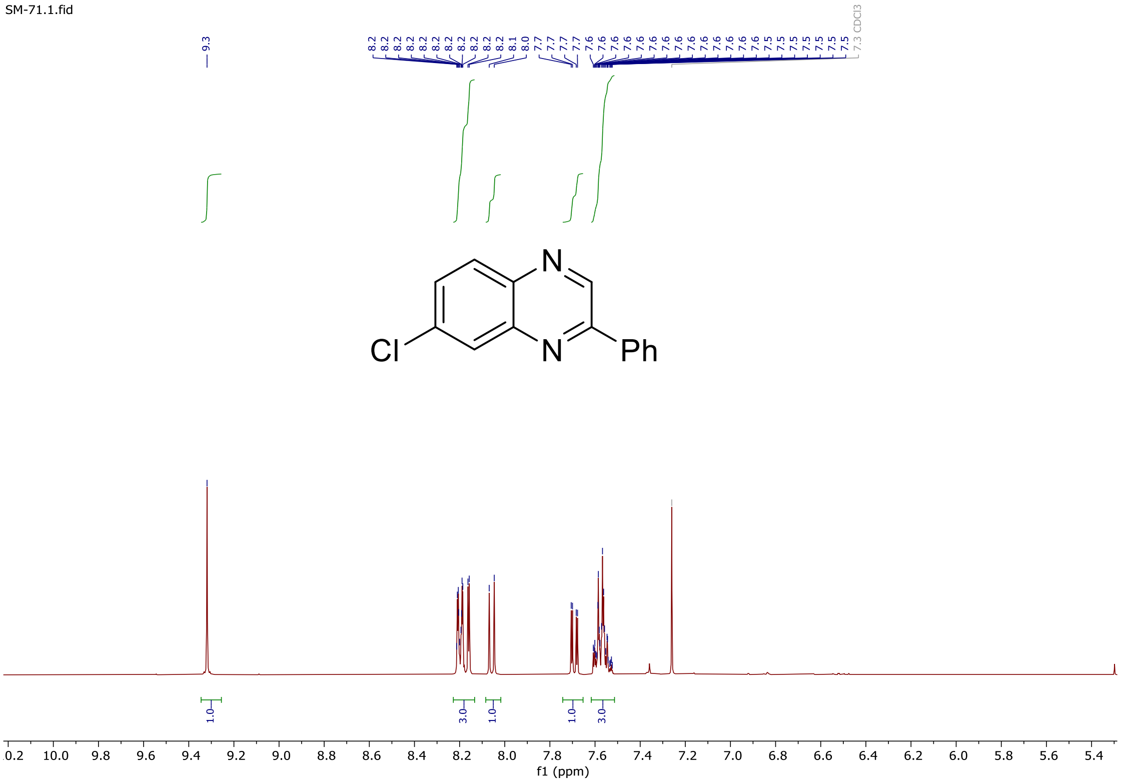

^{13}C NMR of **2k** (101 MHz, $CDCl_3$)

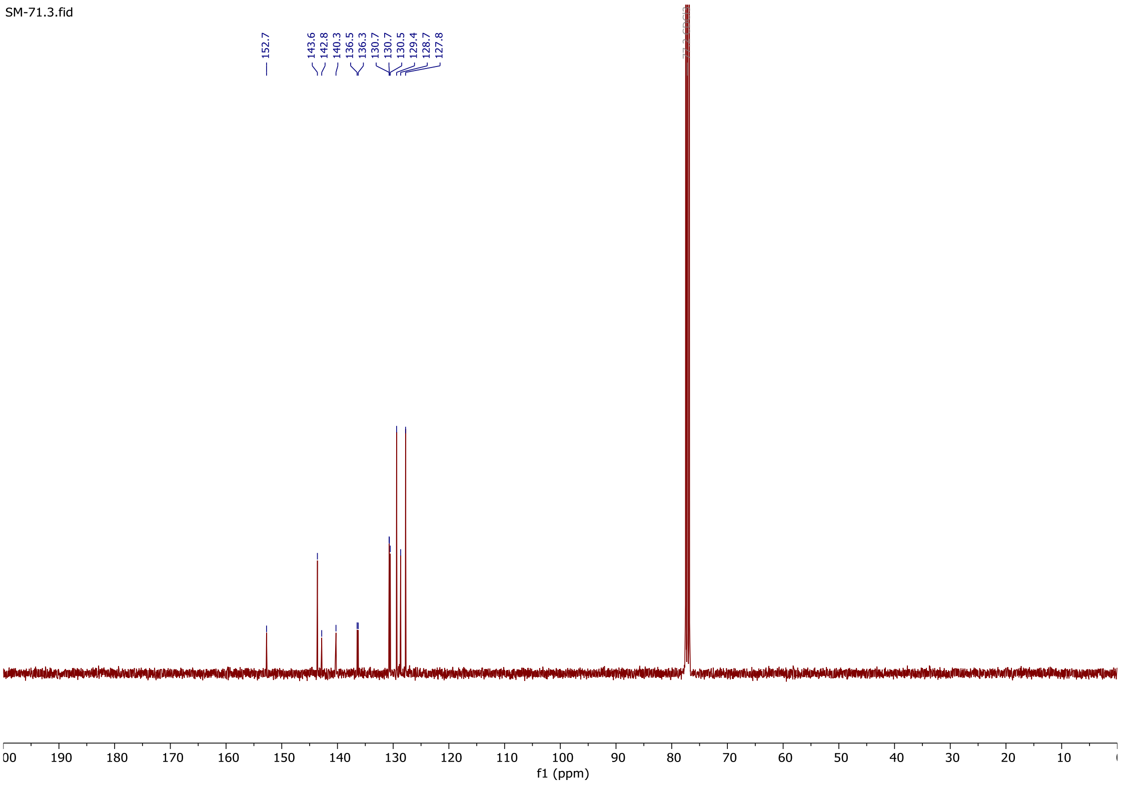

^{1}H NMR of **2l** (400 MHz, $CDCl_3$)

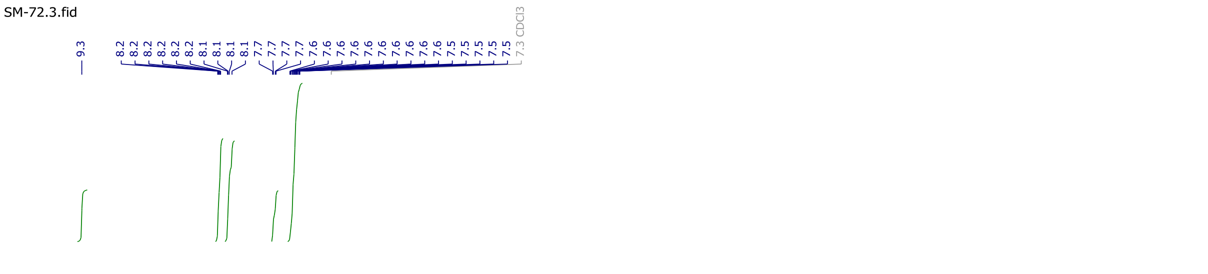

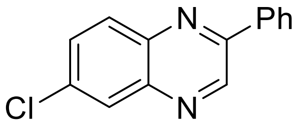

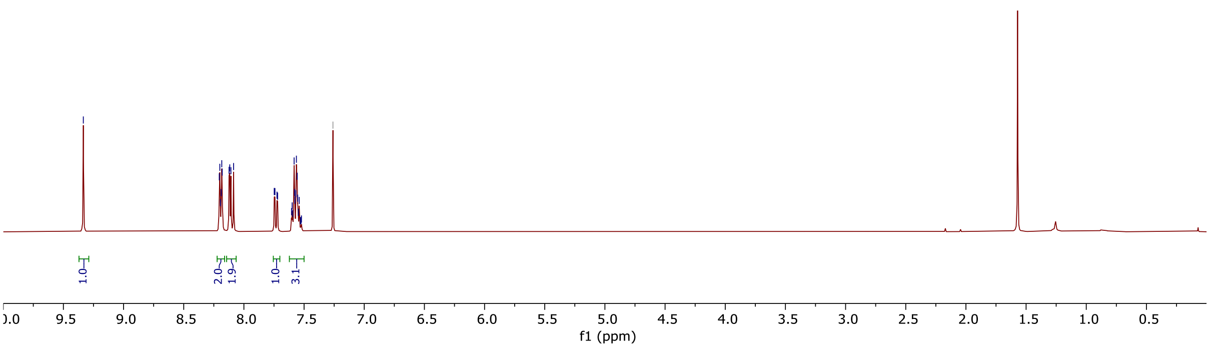

^{13}C NMR of **2l** (101 MHz, $CDCl_3$)

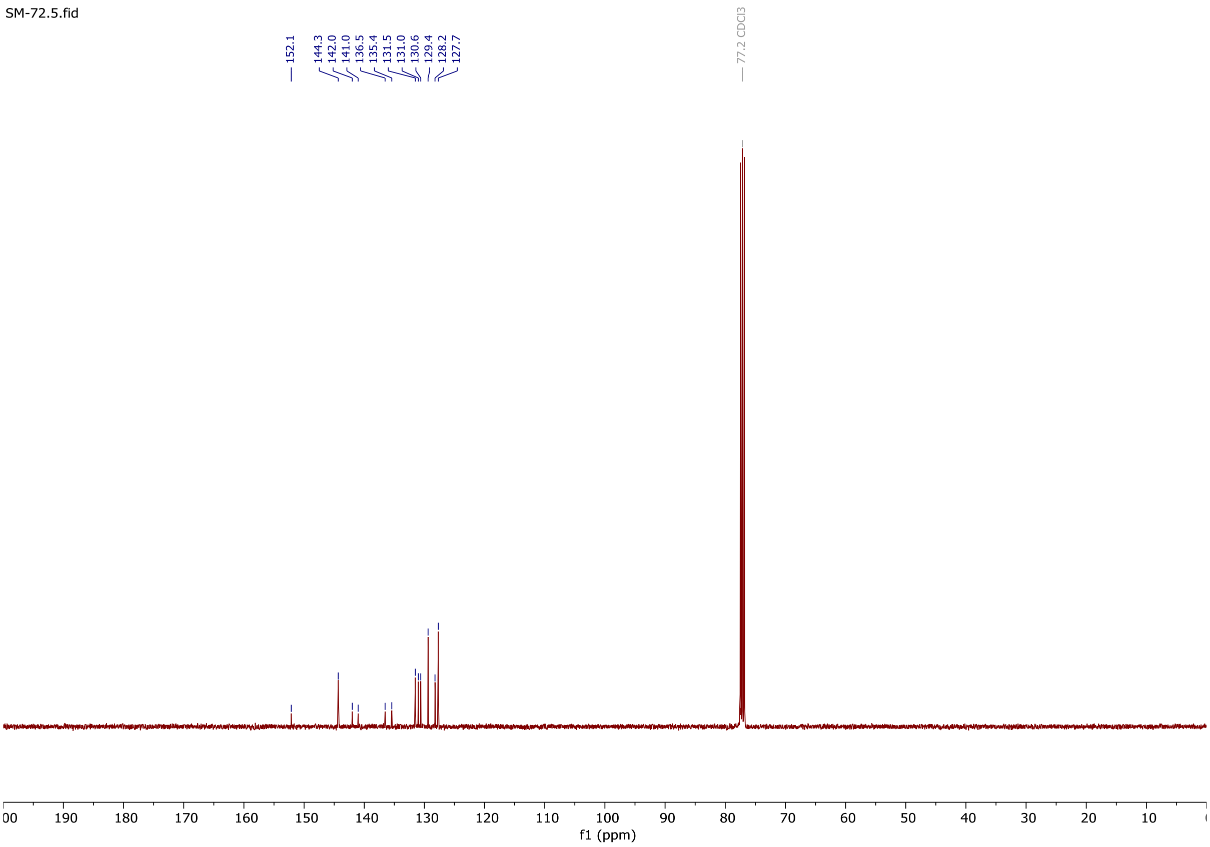

^{1}H NMR of **2m** (400 MHz, $CDCl_3$)

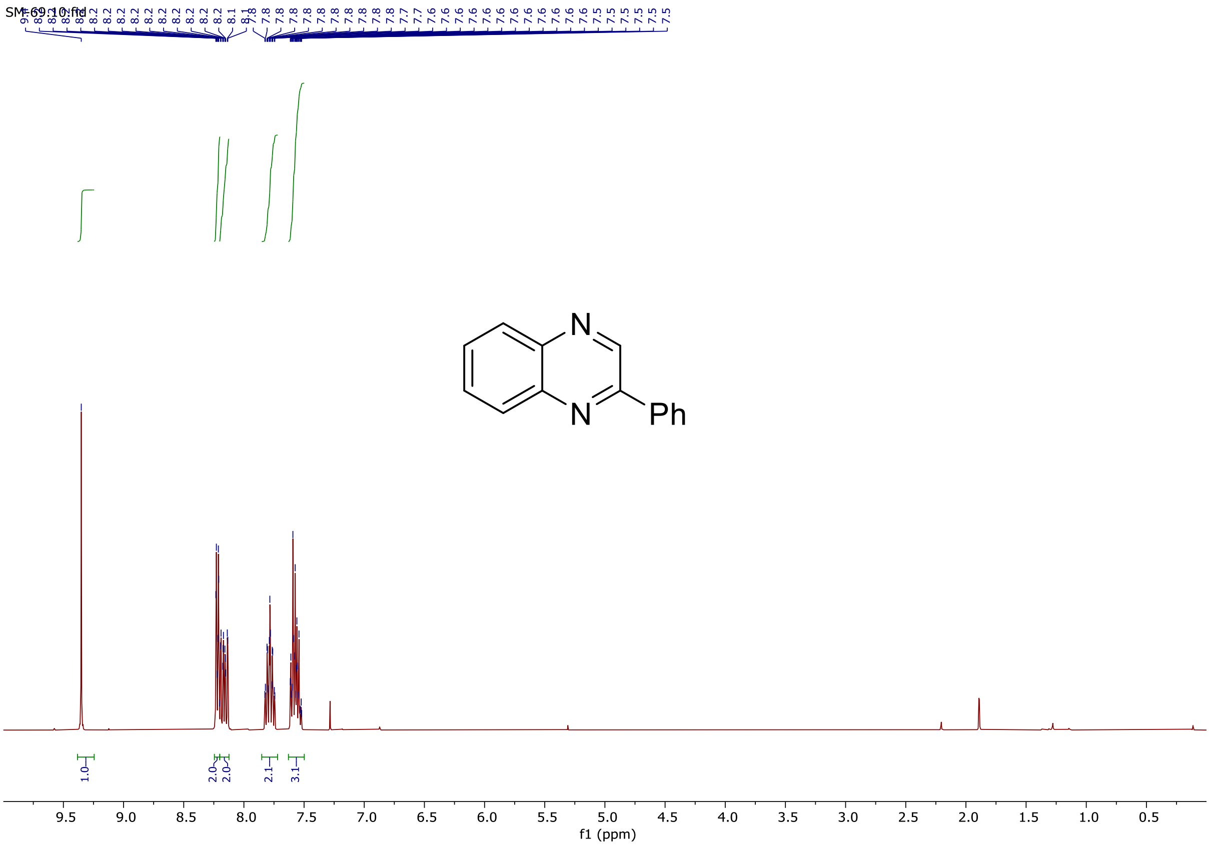

^{13}C NMR of **2m** (101 MHz, $CDCl_3$)

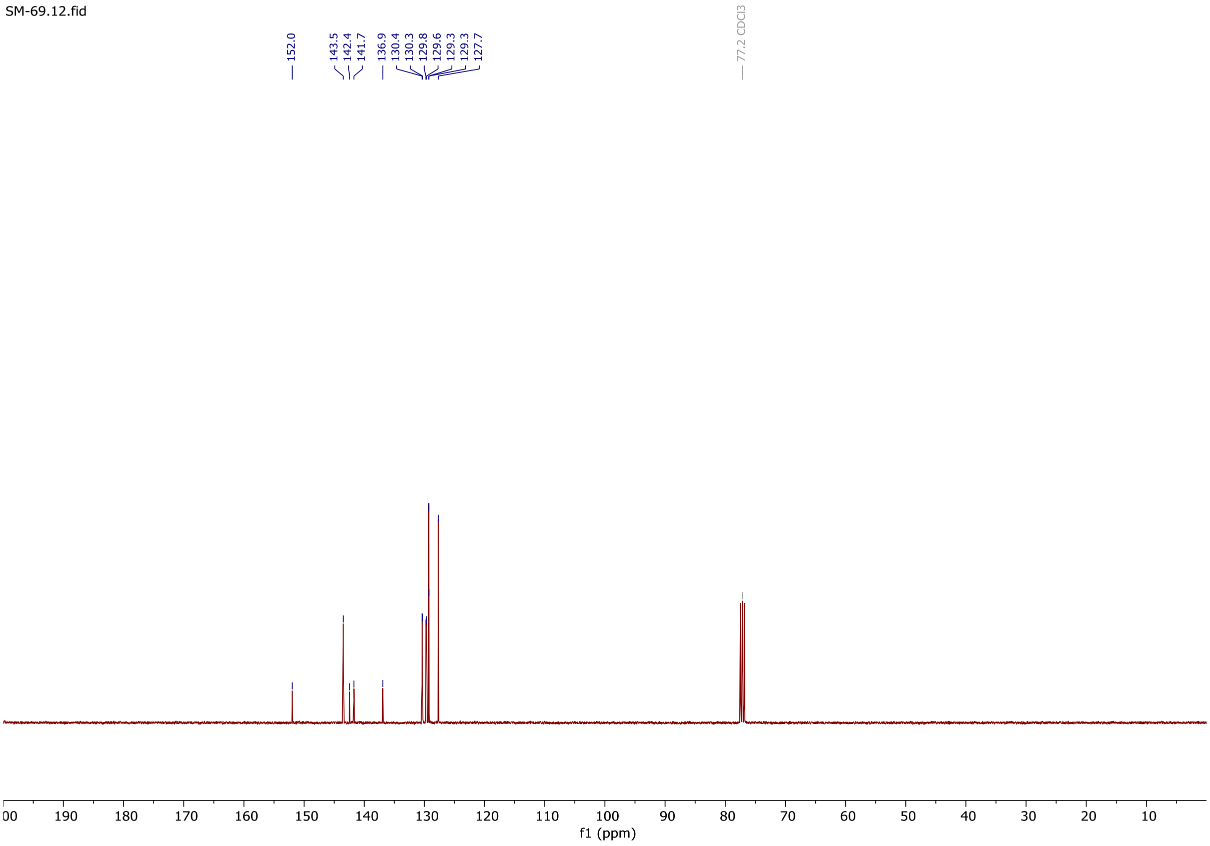

^{1}H NMR of **2n** (400 MHz, $CDCl_3$)

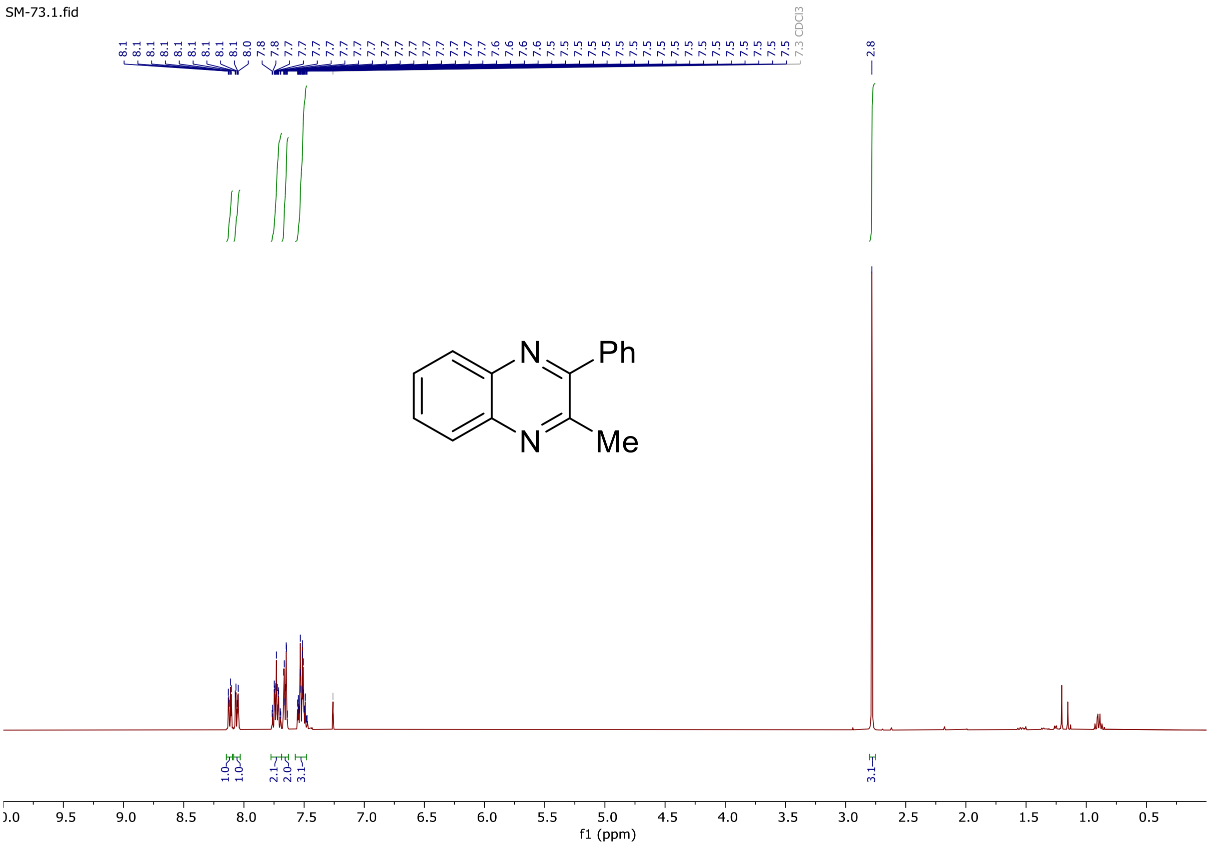

^{13}C NMR of **2n** (101 MHz, $CDCl_3$)

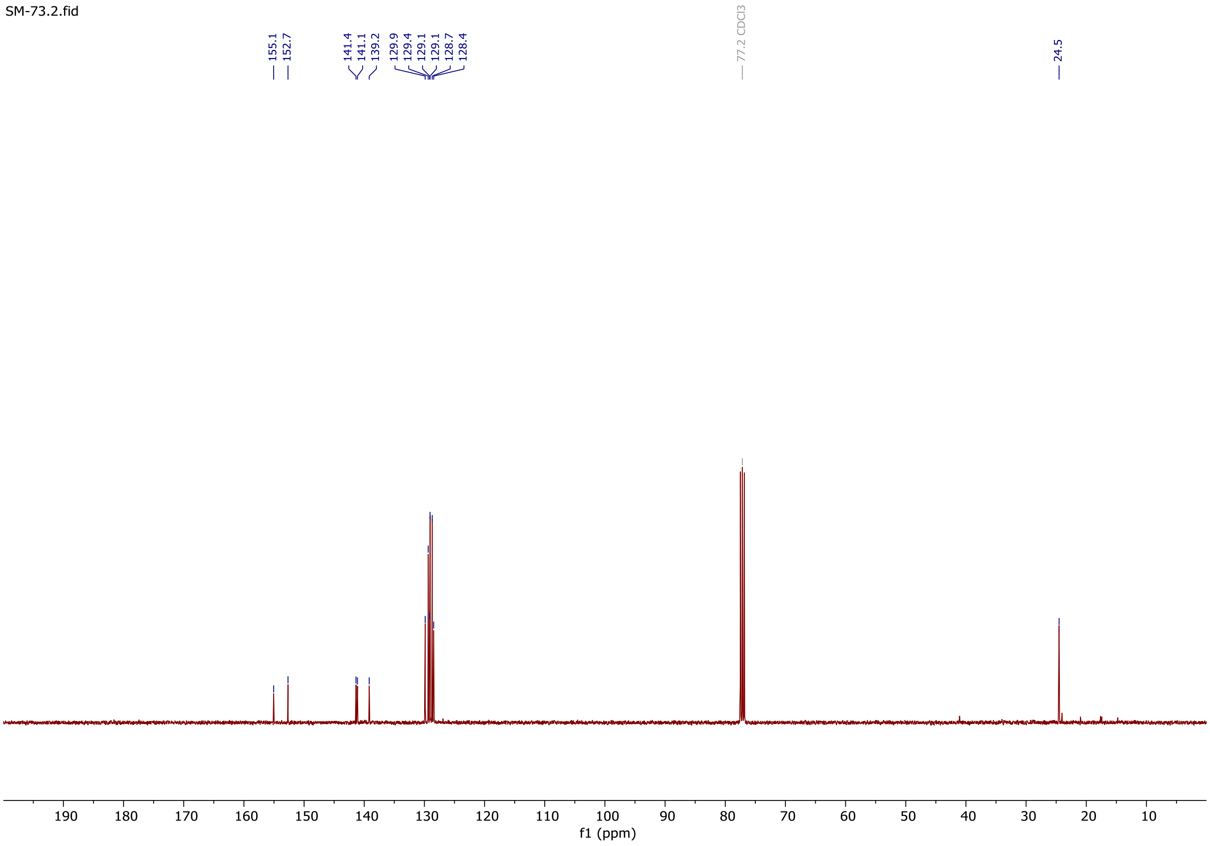

^{1}H NMR of **4a** (400 MHz, $CDCl_3$)

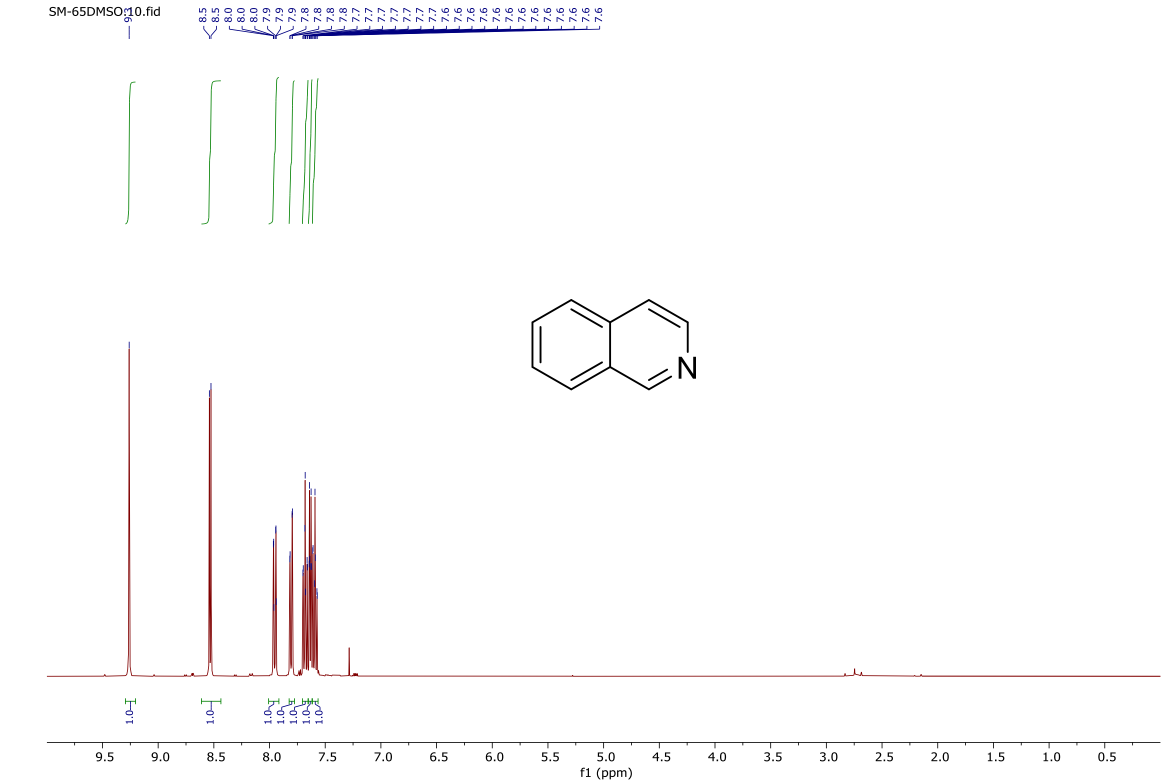

^{13}C NMR of **4a** (101 MHz, $CDCl_3$)

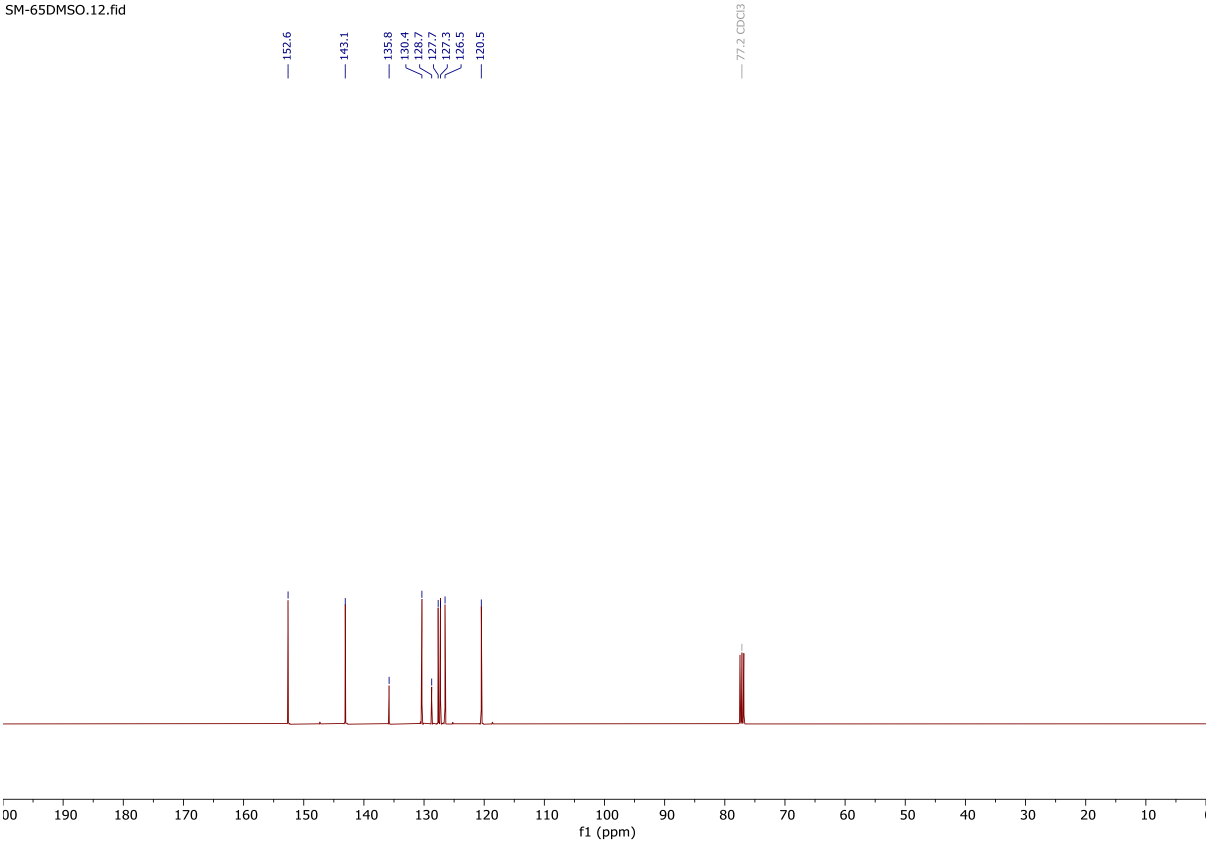

^{1}H NMR of **4b** (400 MHz, $CDCl_3$)

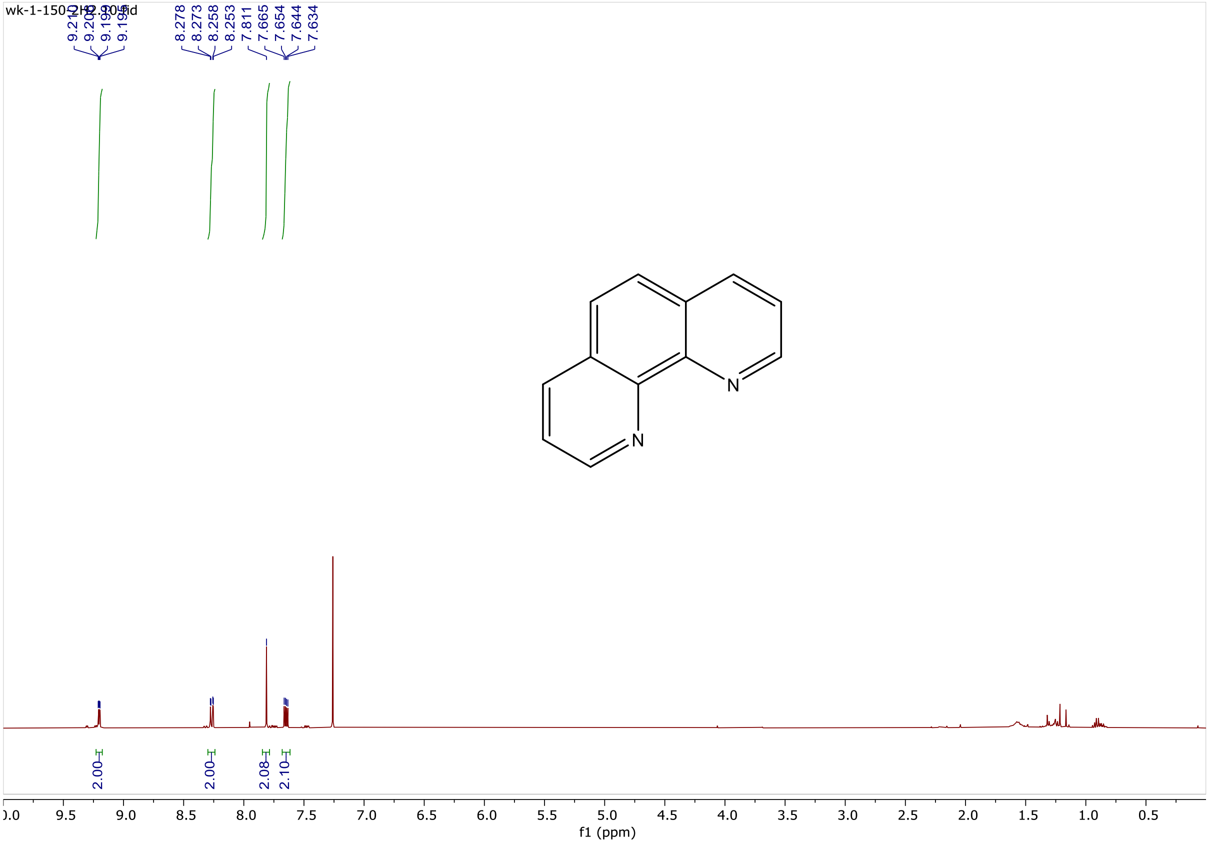

^{13}C NMR of **4b** (101 MHz, $CDCl_3$)

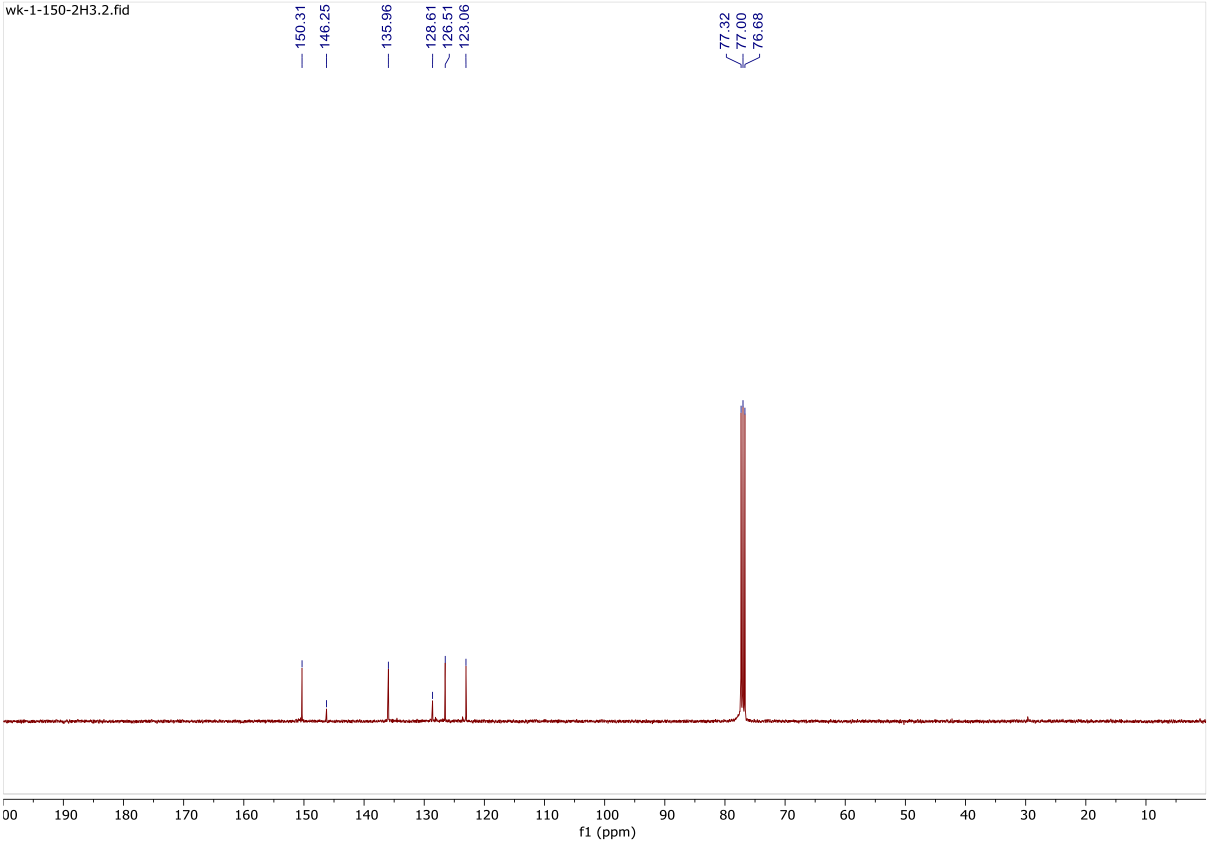

^1H NMR of **4c** (400 MHz, $CDCl_3$)

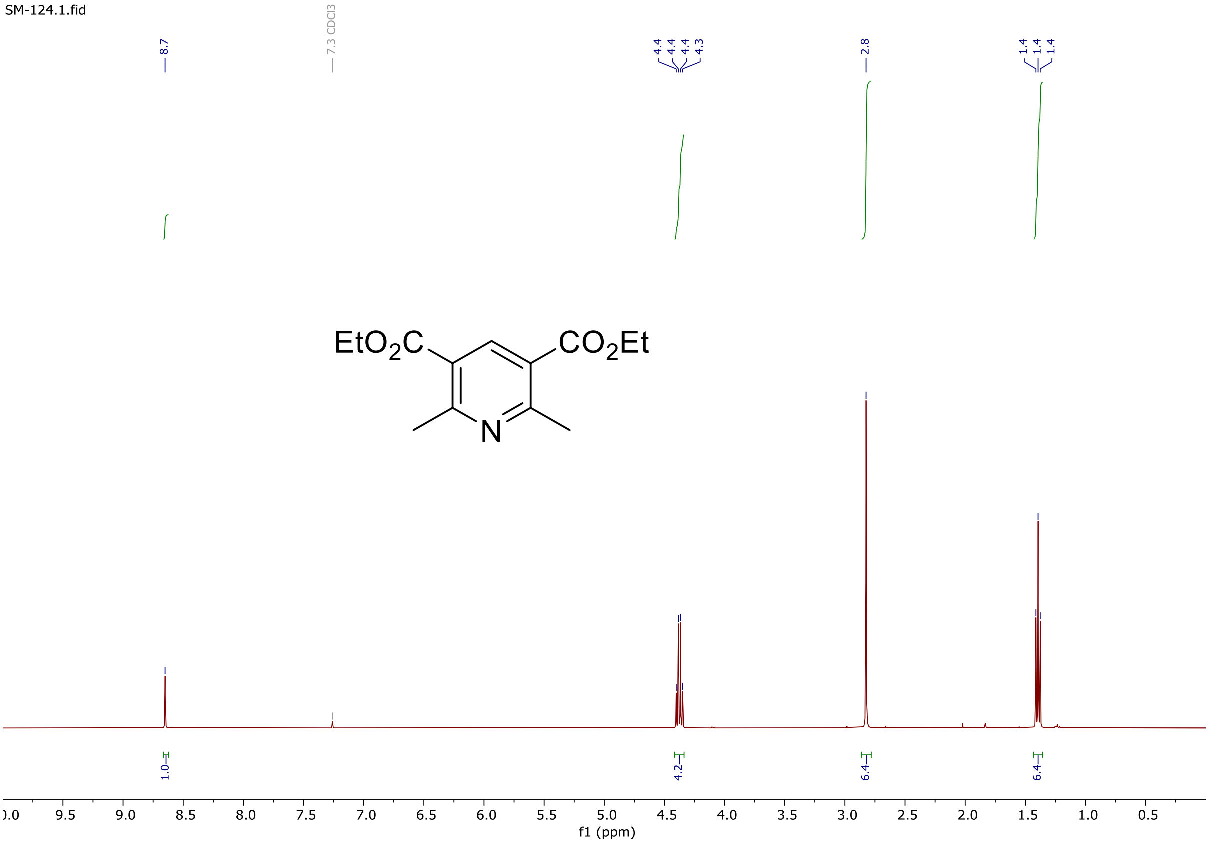

^{13}C NMR of **4c** (101 MHz, $CDCl_3$)

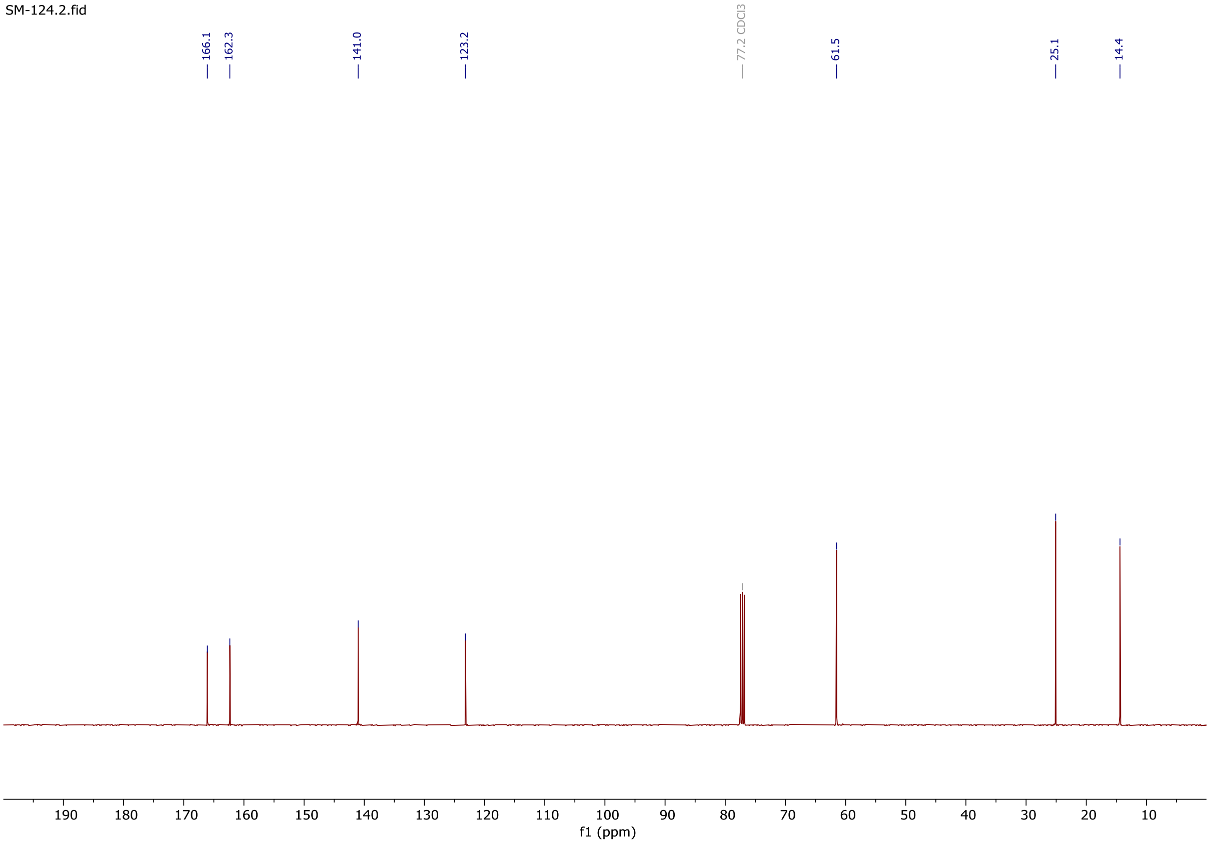

^{1}H NMR of **4d** (400 MHz, $CDCl_3$)

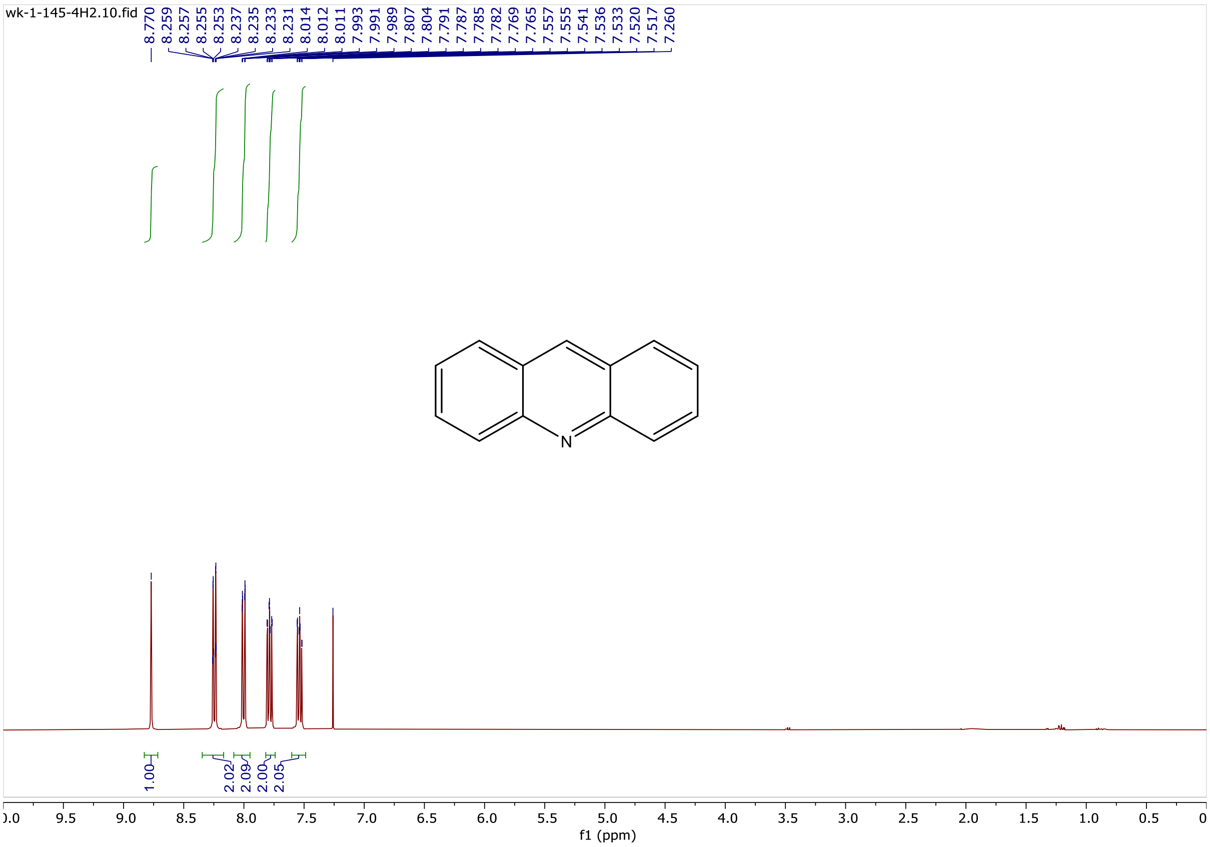

^{13}C NMR of **4d** (101 MHz, $CDCl_3$)

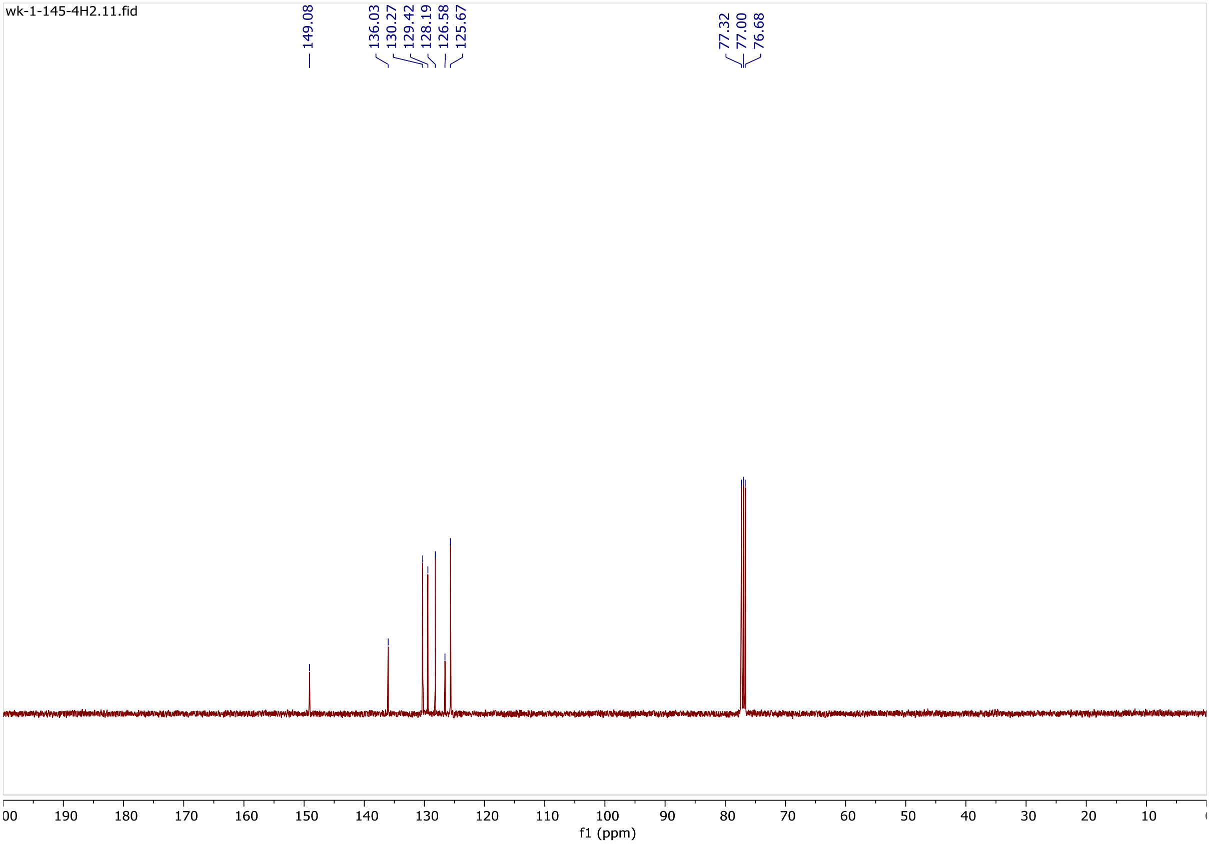

^{1}H NMR of **4e** (400 MHz, $CDCl_3$)

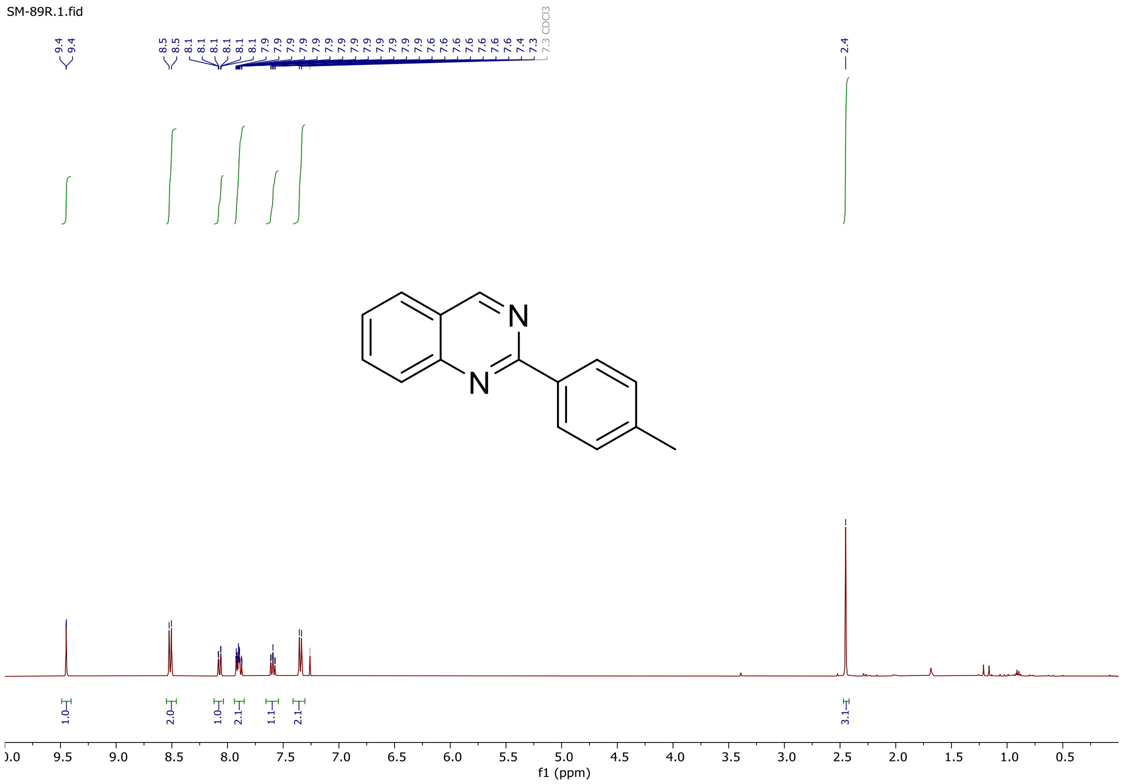

^{13}C NMR of **4e** (101 MHz, $CDCl_3$)

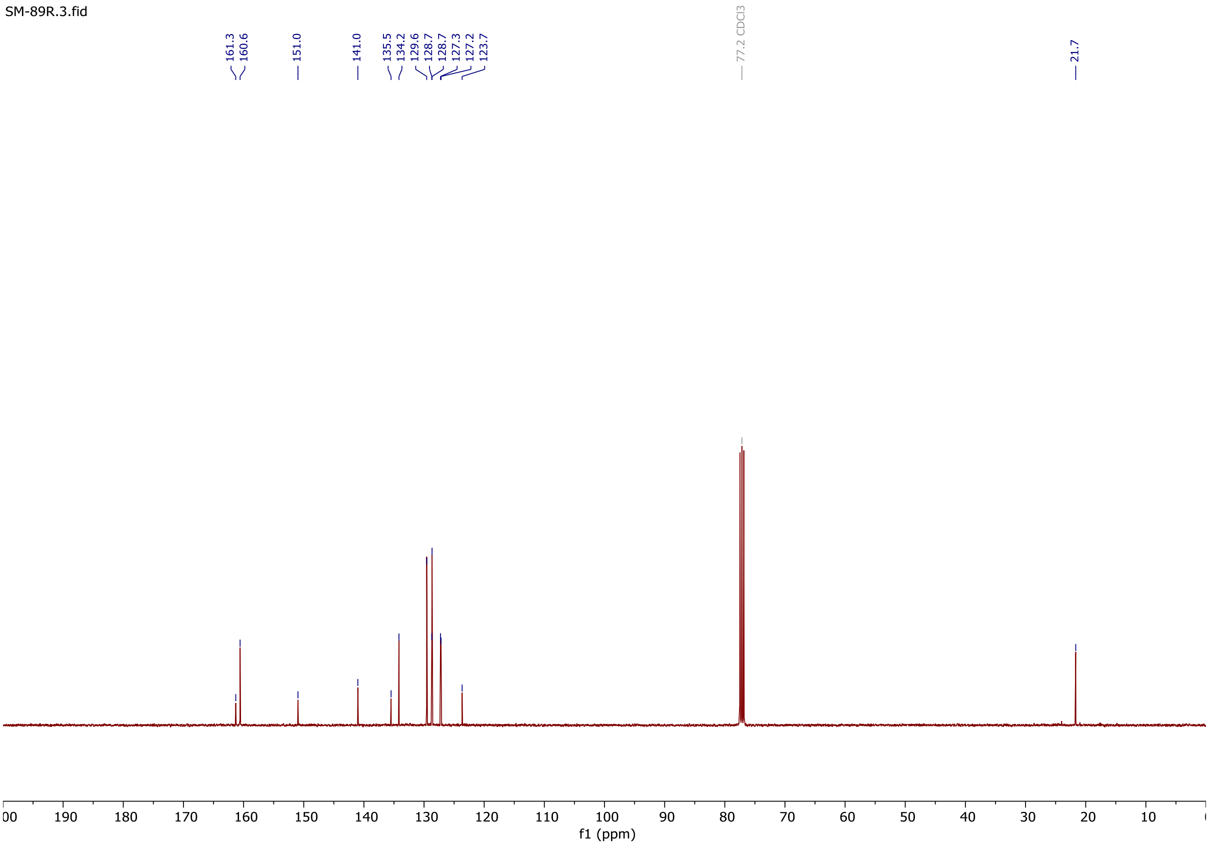

^{13}C NMR of **6a** (400 MHz, $CDCl_3$)

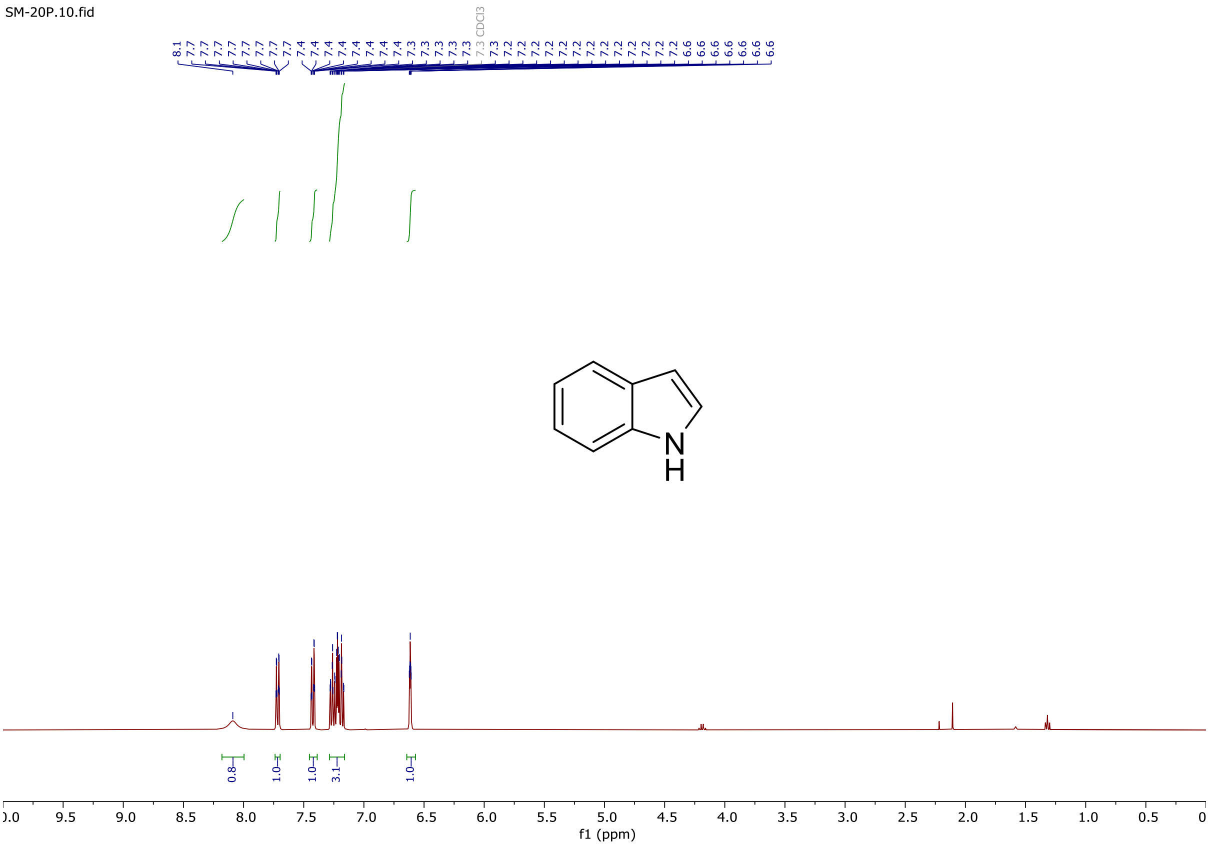

^{13}C NMR of **6a** (101 MHz, $CDCl_3$)

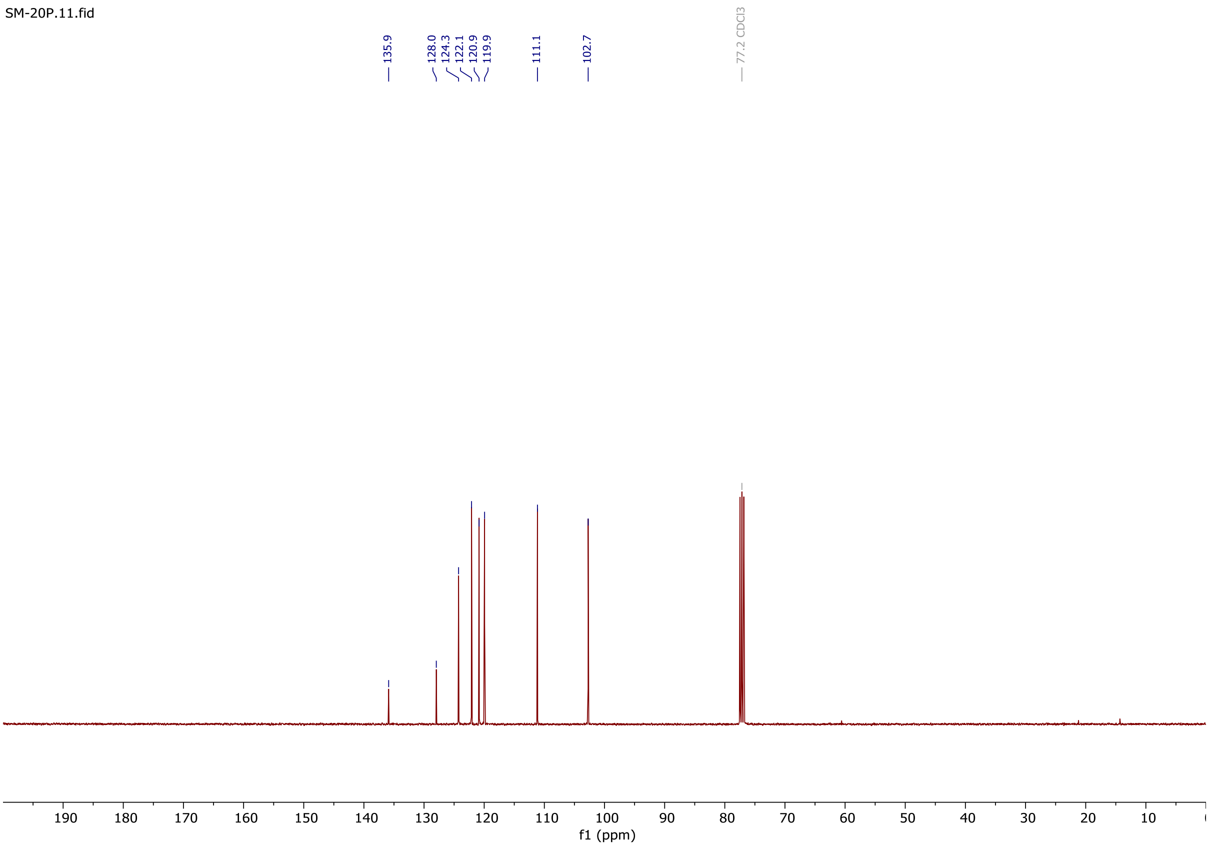

^{1}H NMR of **6b** (400 MHz, $CDCl_3$)

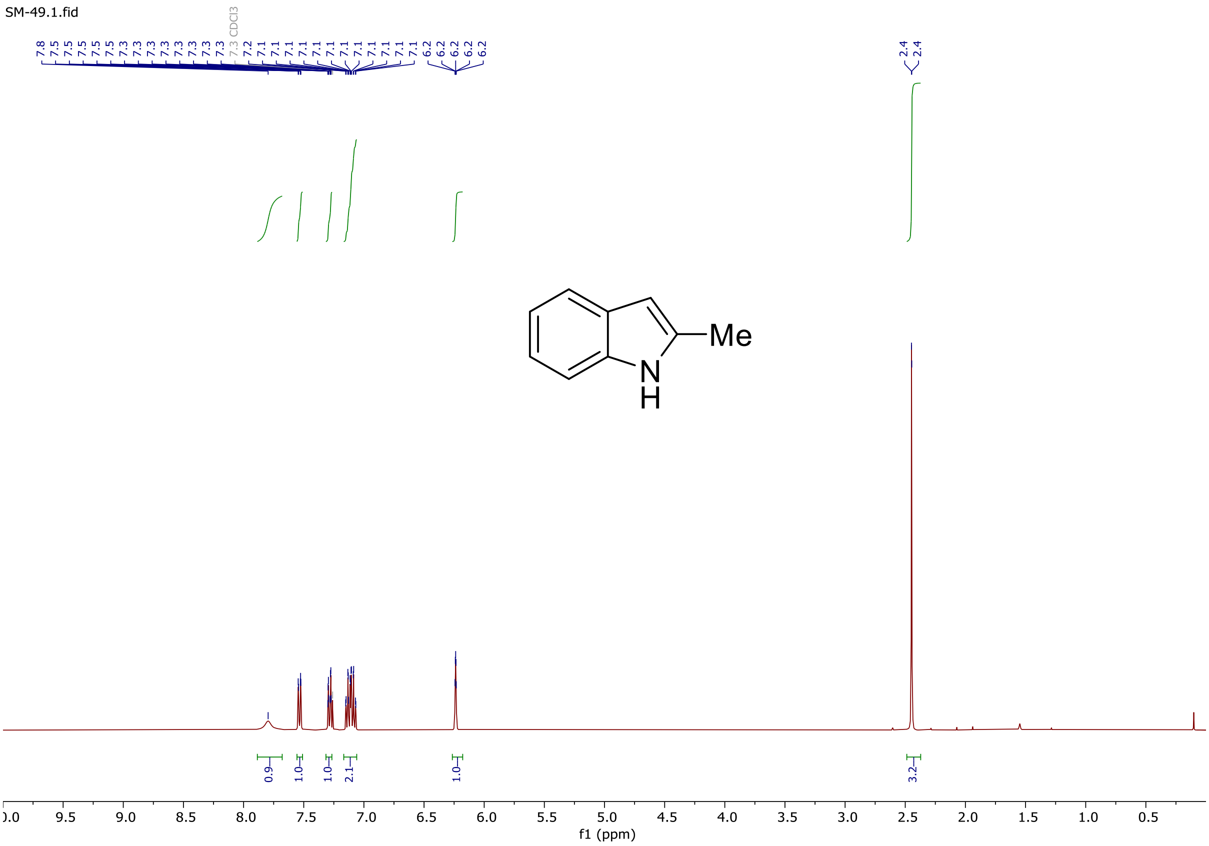

^{13}C NMR of **6b** (101 MHz, $CDCl_3$)

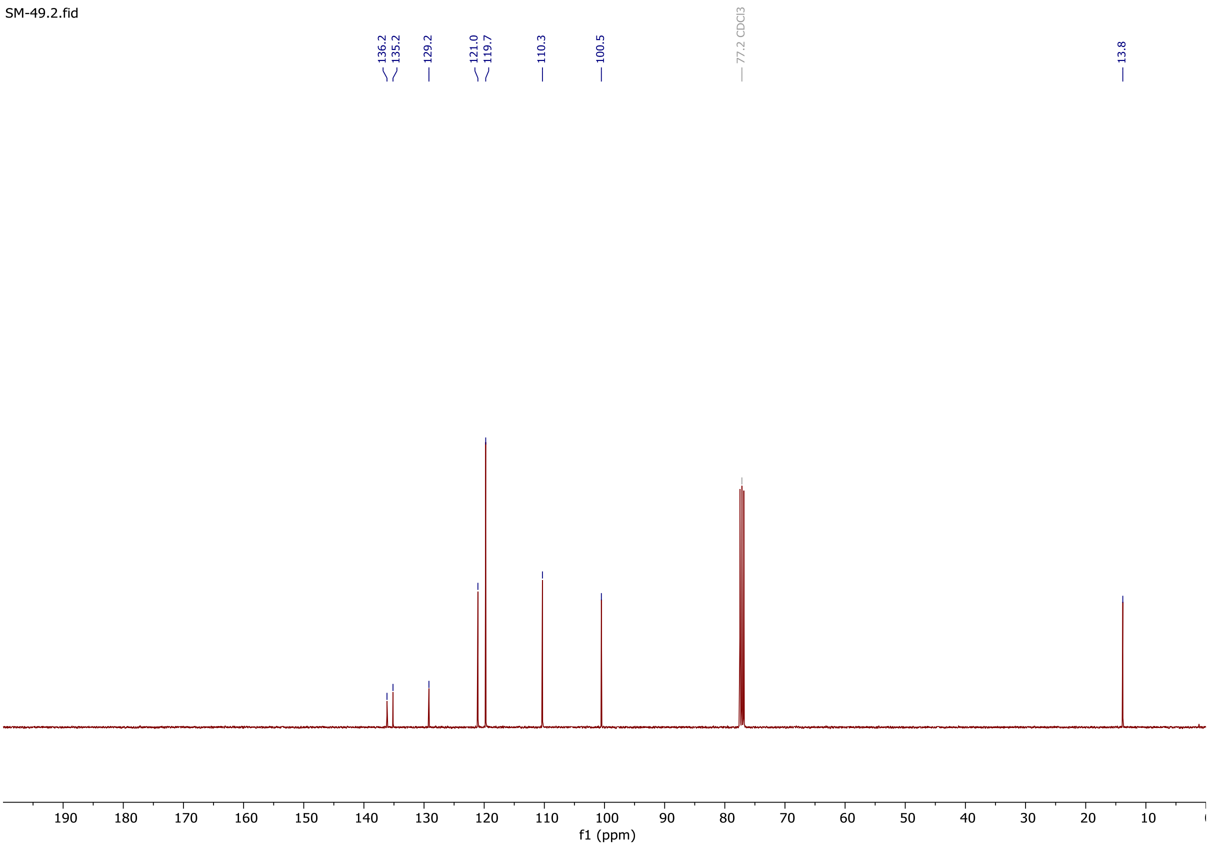

^{1}H NMR of **6c** (400 MHz, $CDCl_3$)

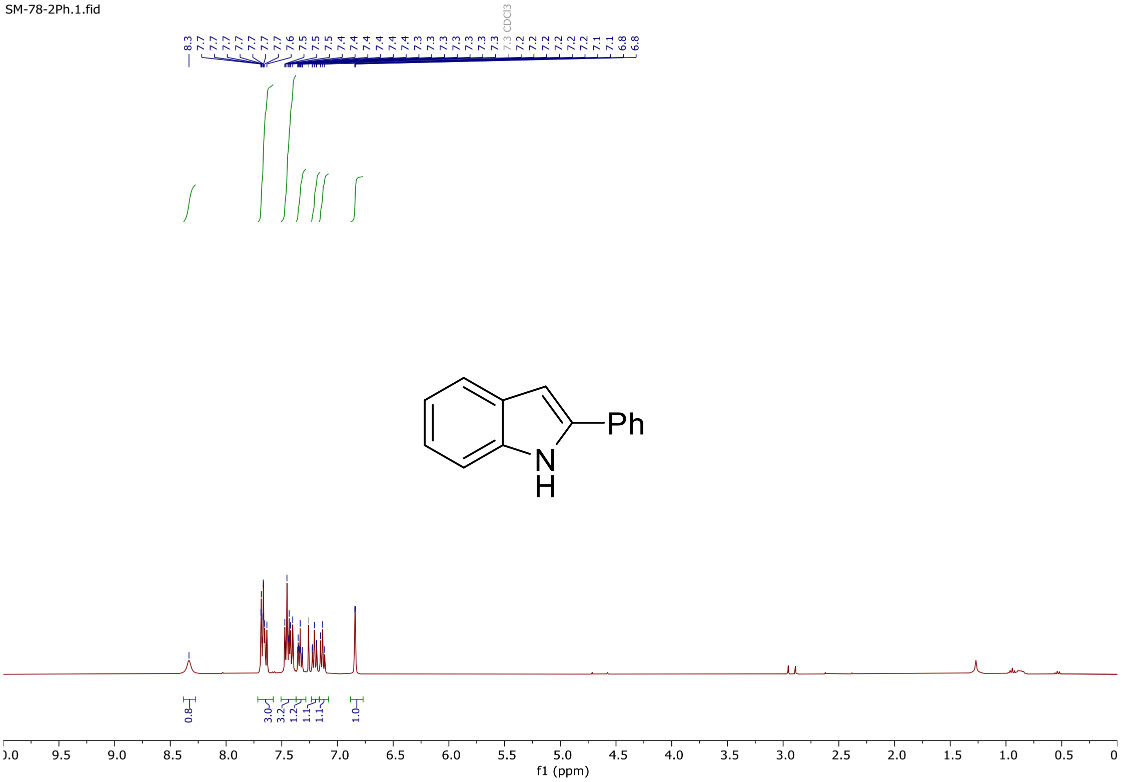

^{13}C NMR of **6c** (101 MHz, $CDCl_3$)

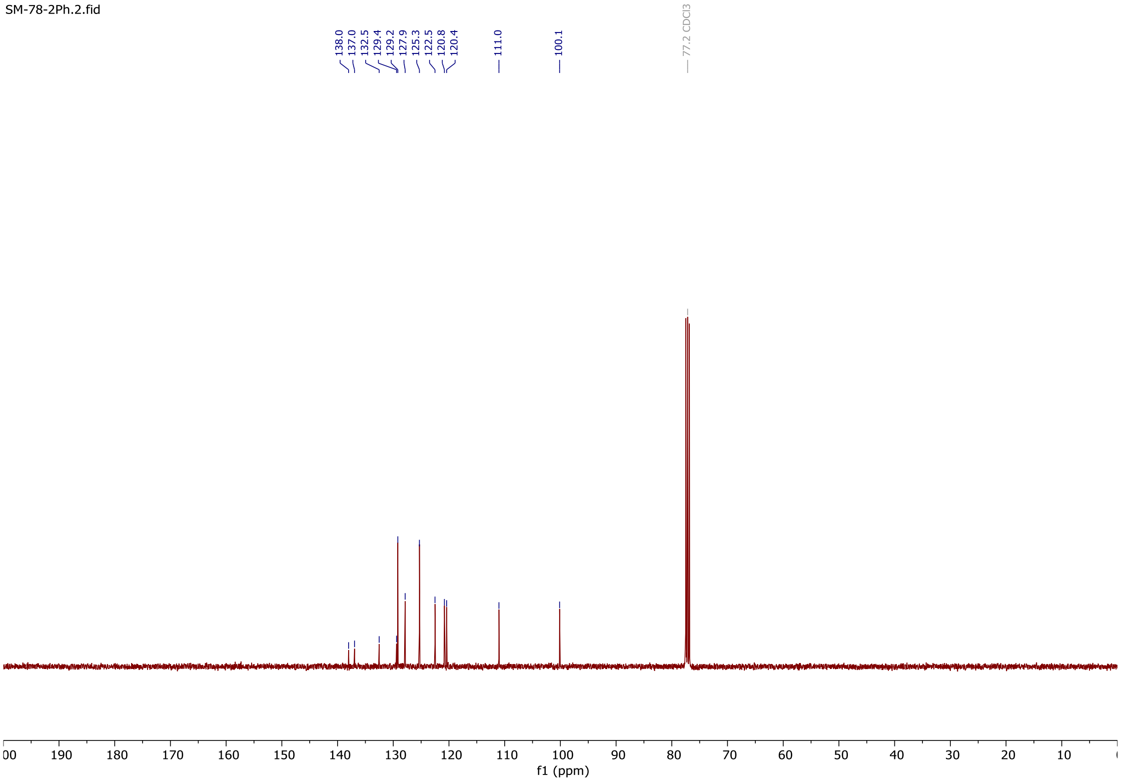

^{1}H NMR of **6d** (400 MHz, $CDCl_3$)

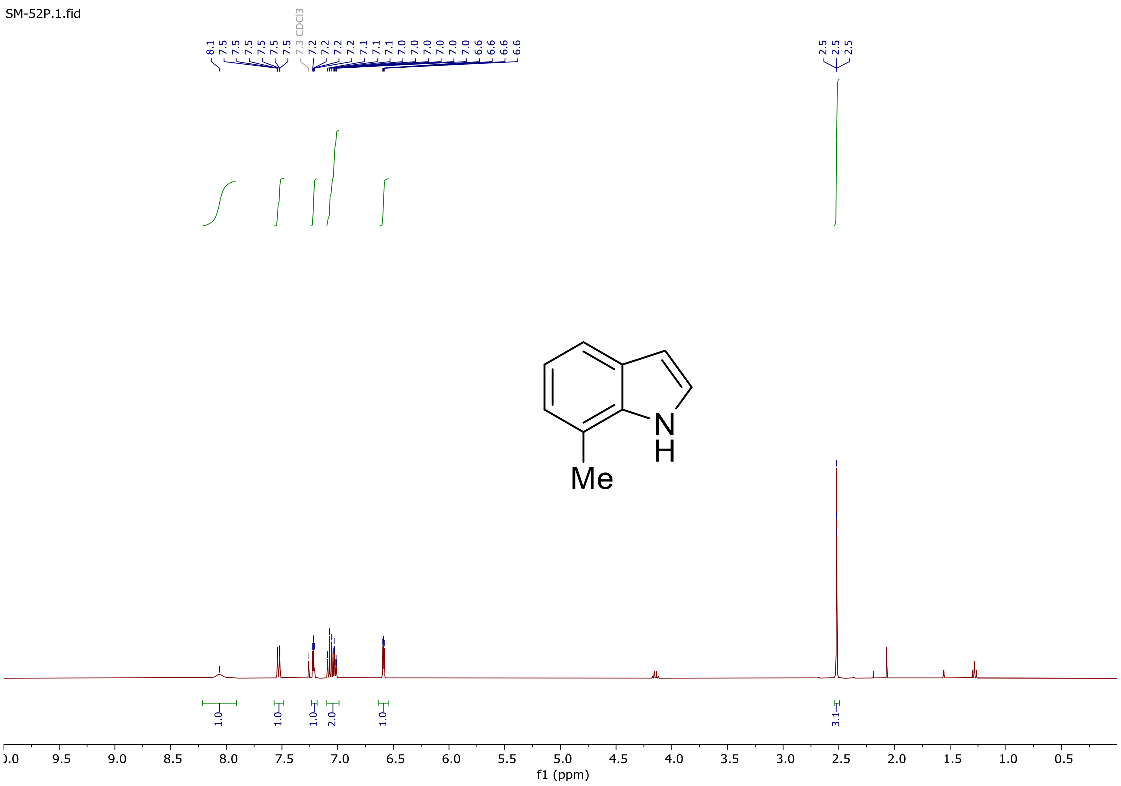

^{13}C NMR of **6d** (101 MHz, $CDCl_3$)

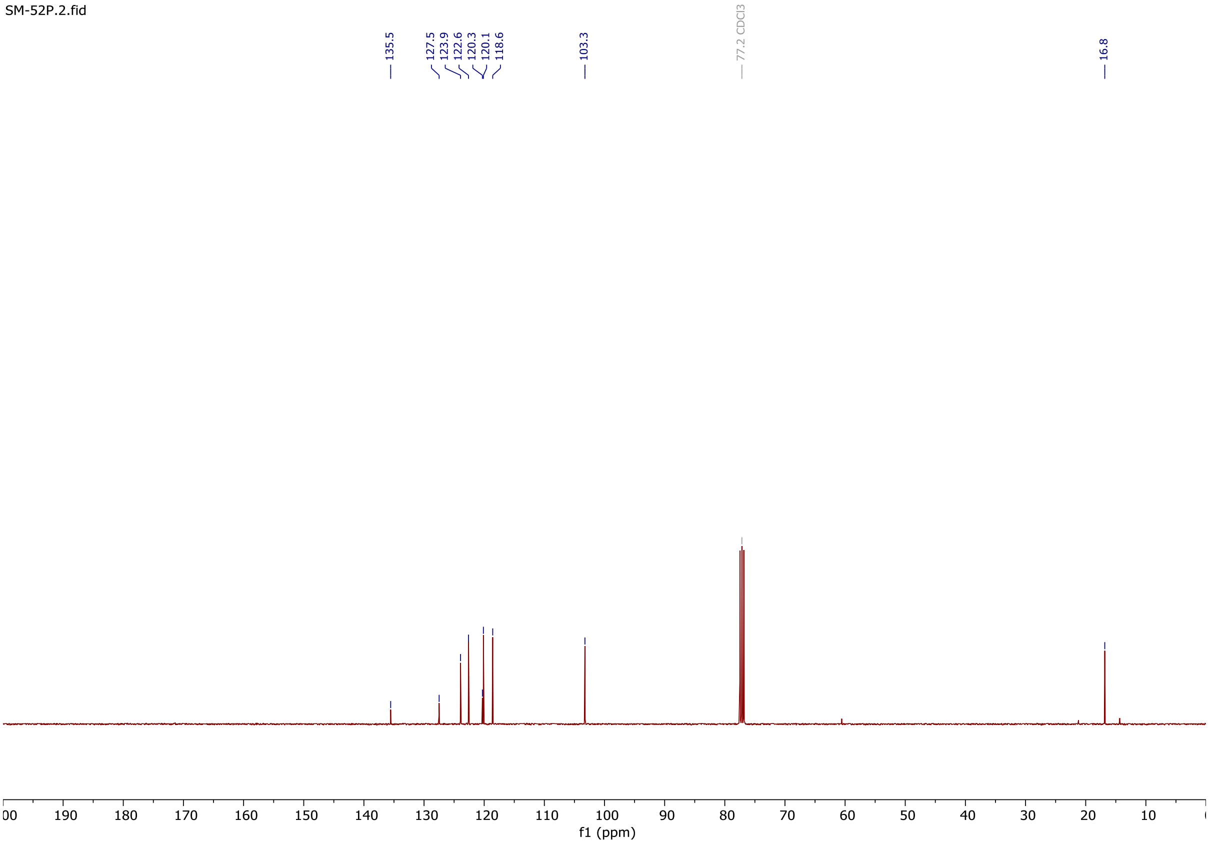

^1H NMR of **6e** (400 MHz, $CDCl_3$)

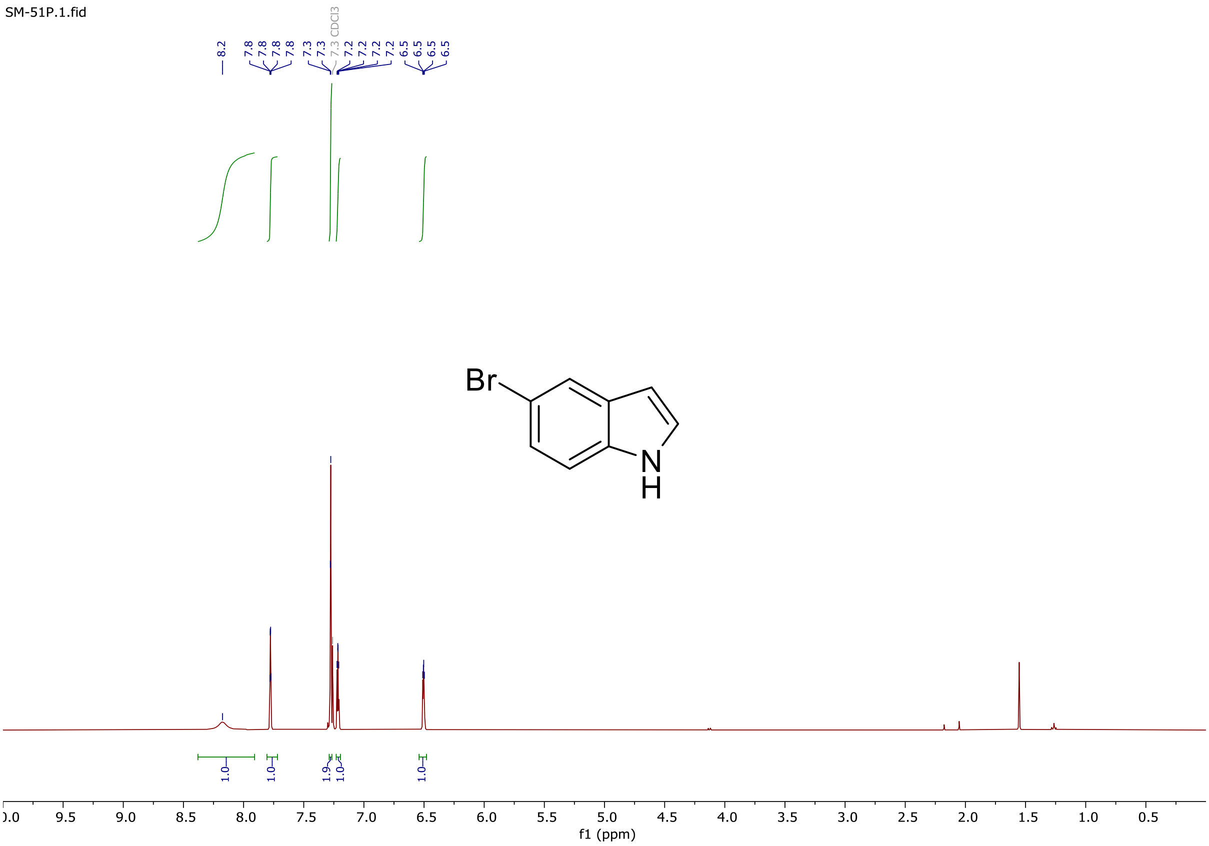

^{13}C NMR of **6e** (101 MHz, $CDCl_3$)

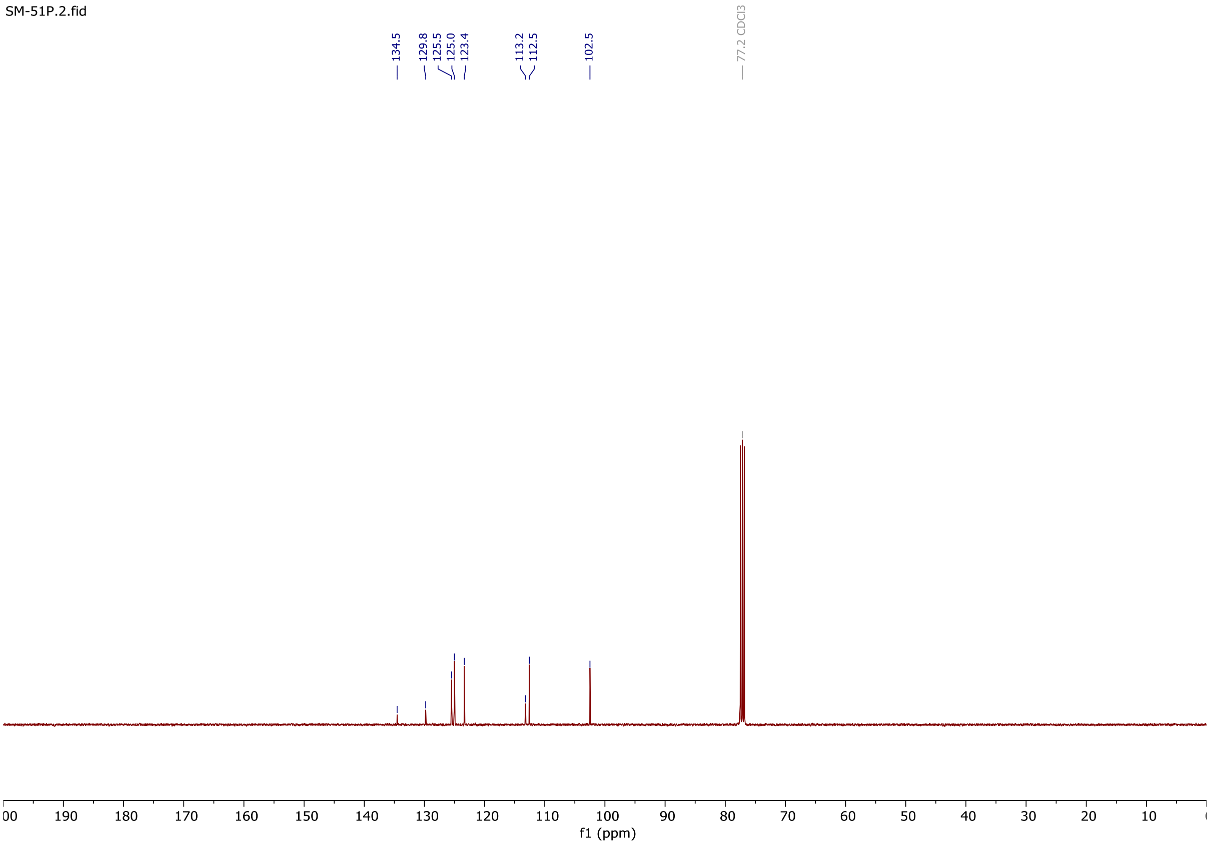

^{1}H NMR of **6f** (400 MHz, $CDCl_3$)

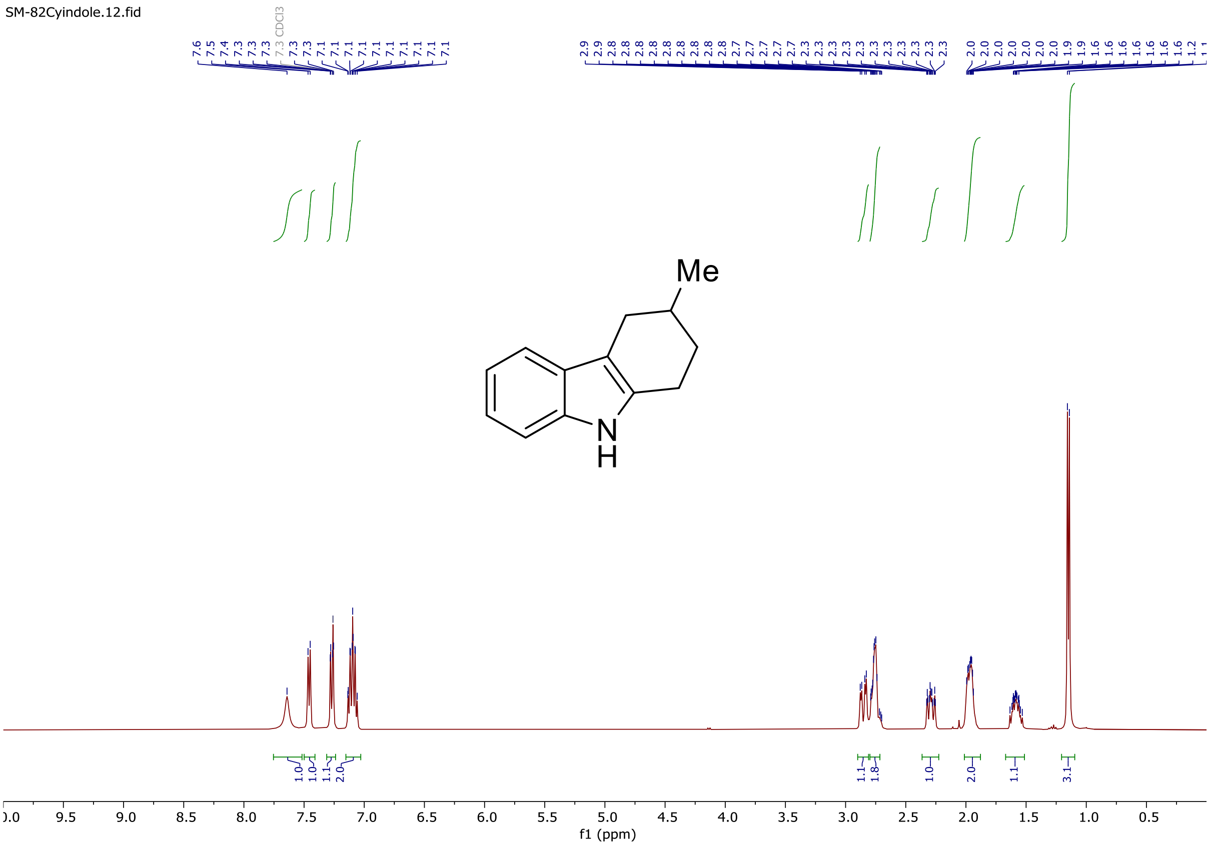

^{13}C NMR of **6f** (101 MHz, $CDCl_3$)

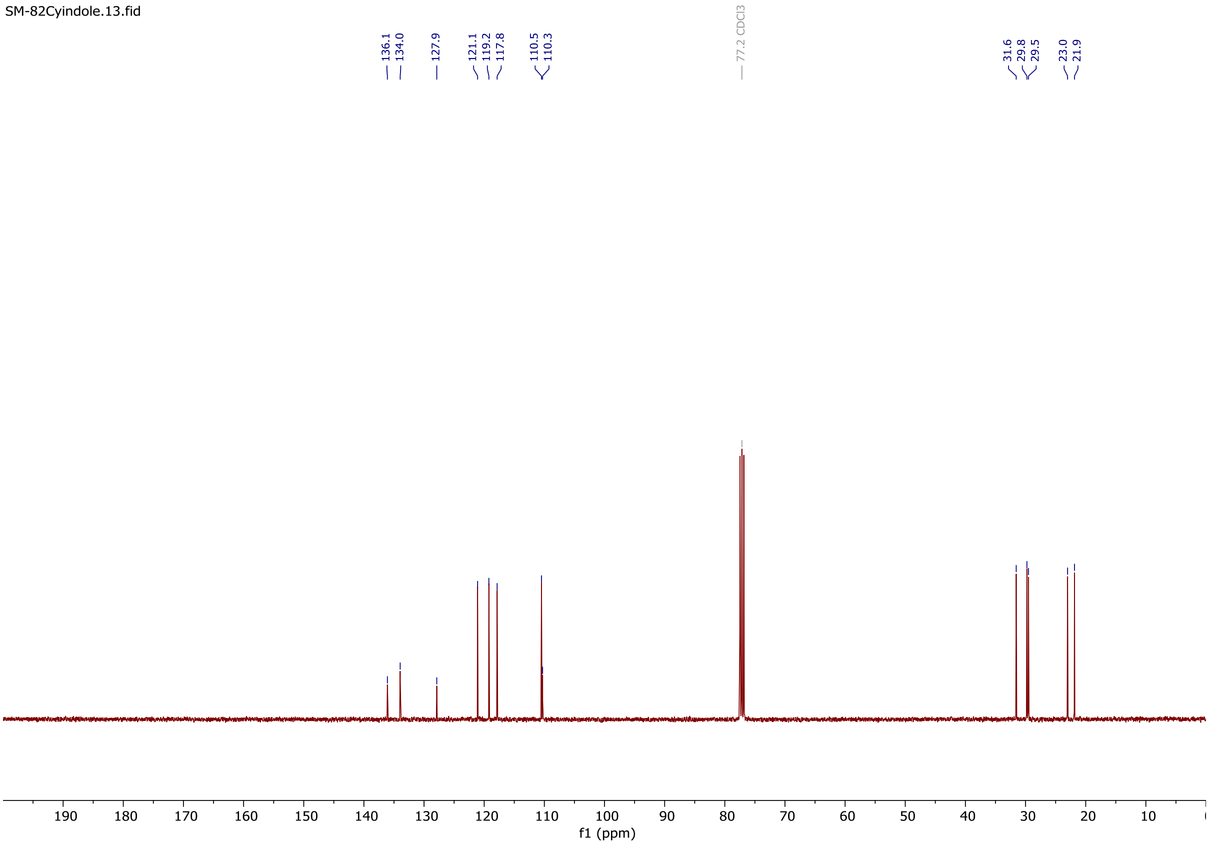

^{1}H NMR of **8a** (400 MHz, $CDCl_3$)

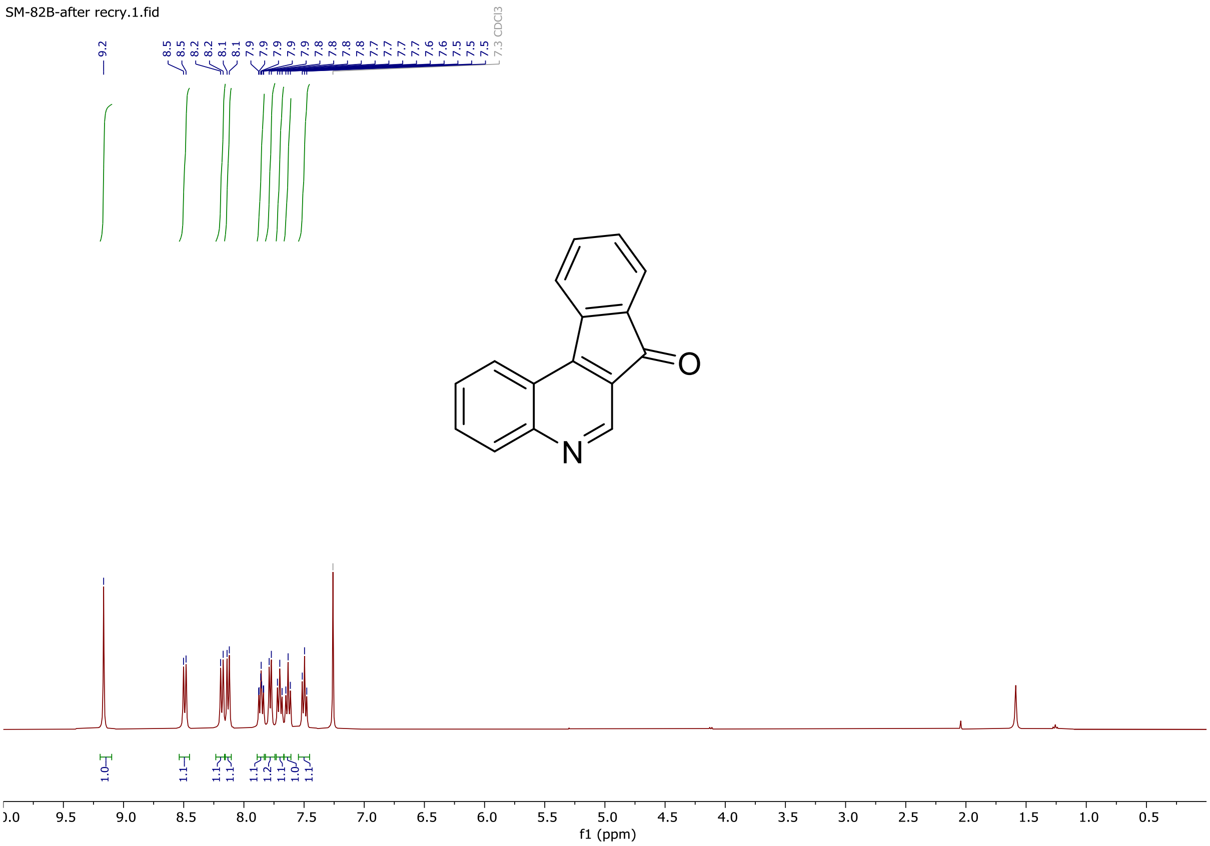

^{13}C NMR of **2z** (101 MHz, $CDCl_3$)

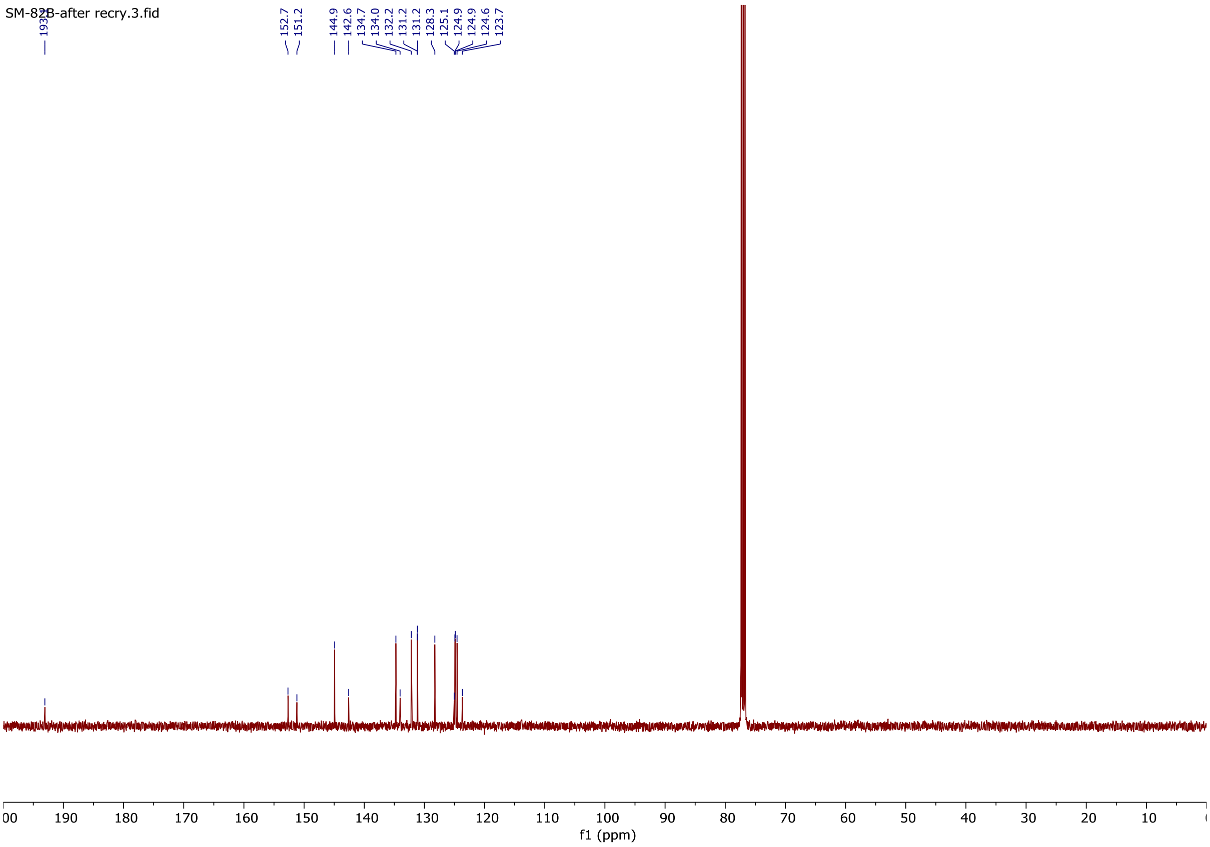

^{1}H NMR of **11a** (400 MHz, $CDCl_3$)

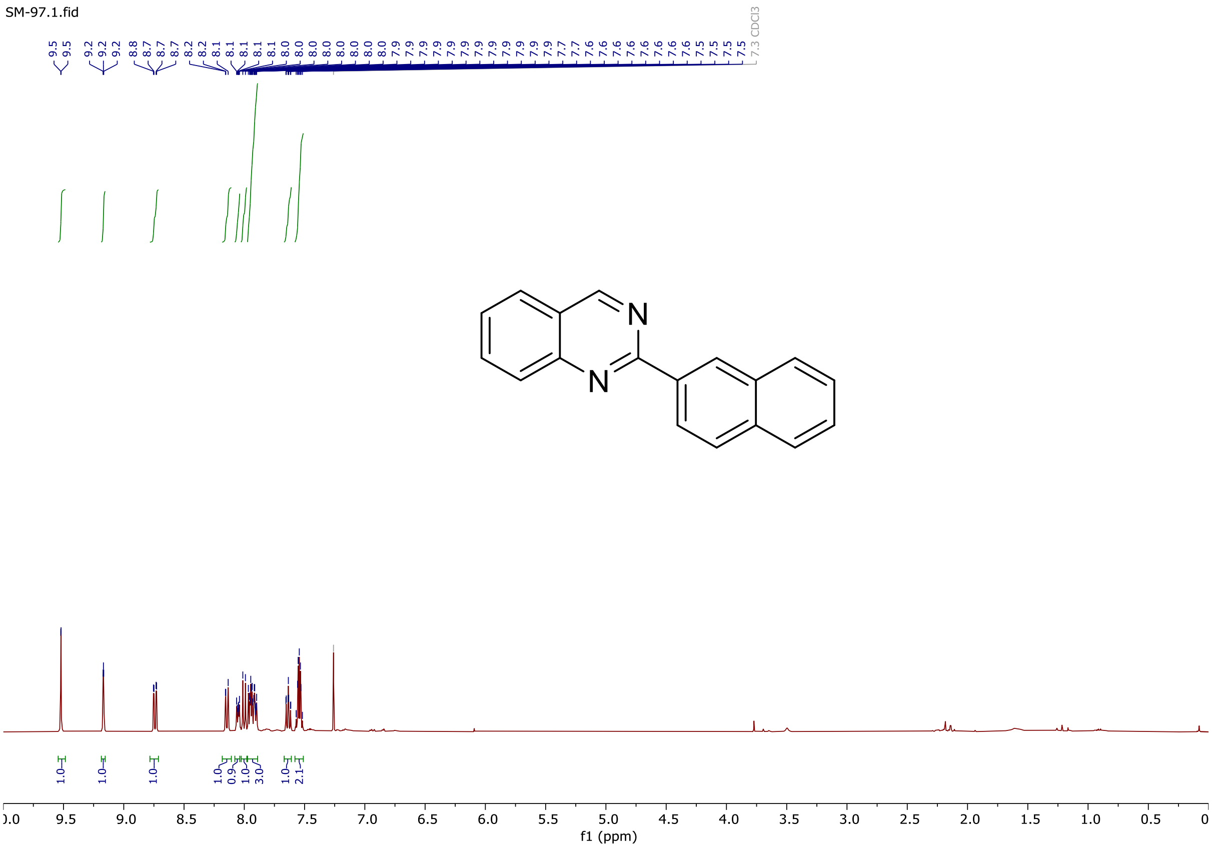

^{13}C NMR of **11a** (101 MHz, $CDCl_3$)

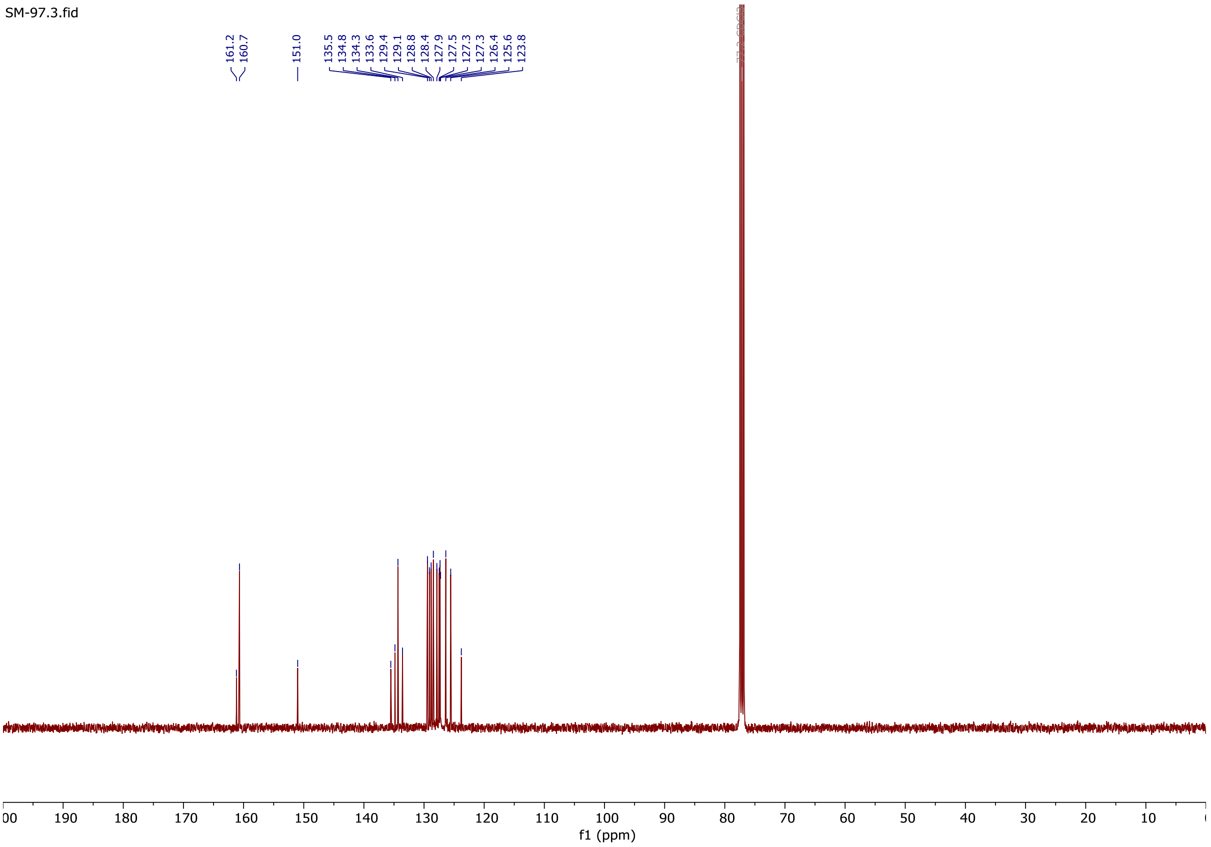

^{1}H NMR of **11b** (400 MHz, $CDCl_3$)

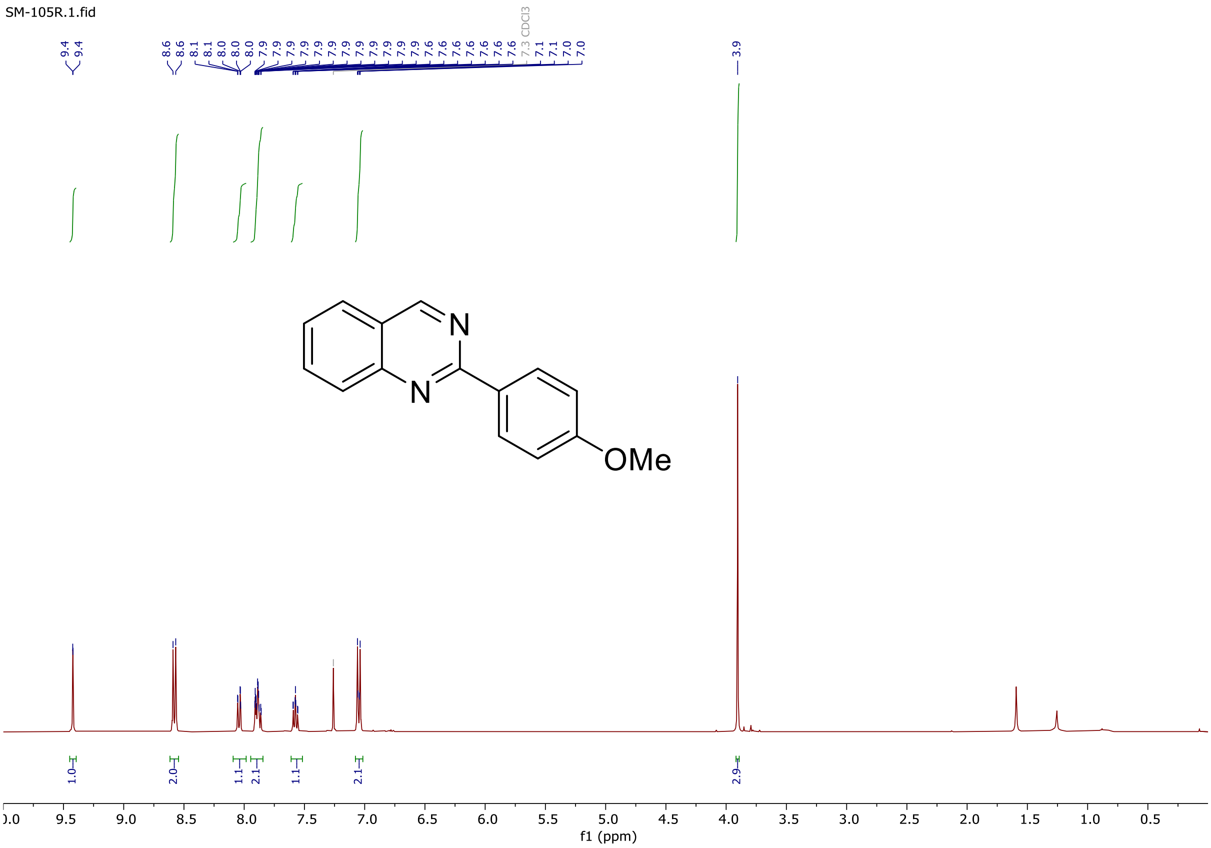

^{13}C NMR of **11b** (101 MHz, $CDCl_3$)

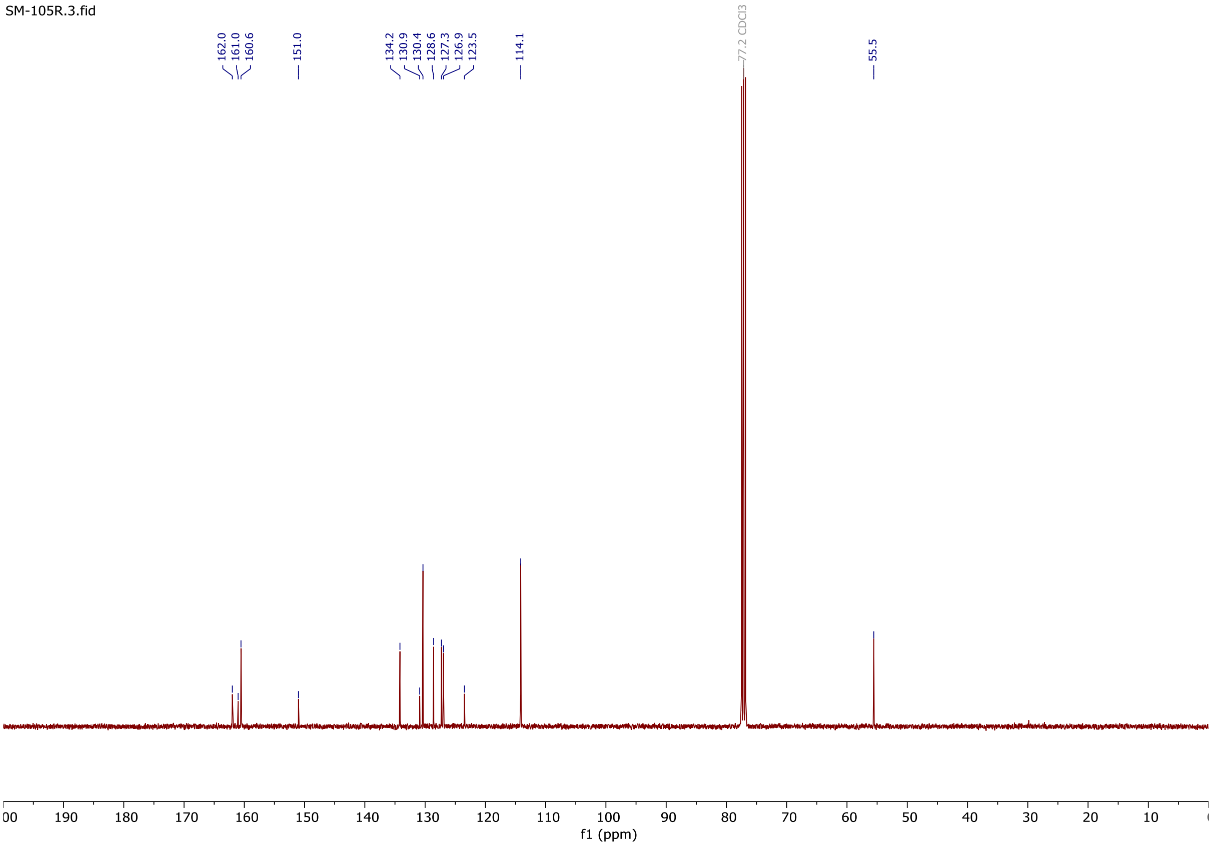

^{1}H NMR of **11c** (400 MHz, $CDCl_3$)

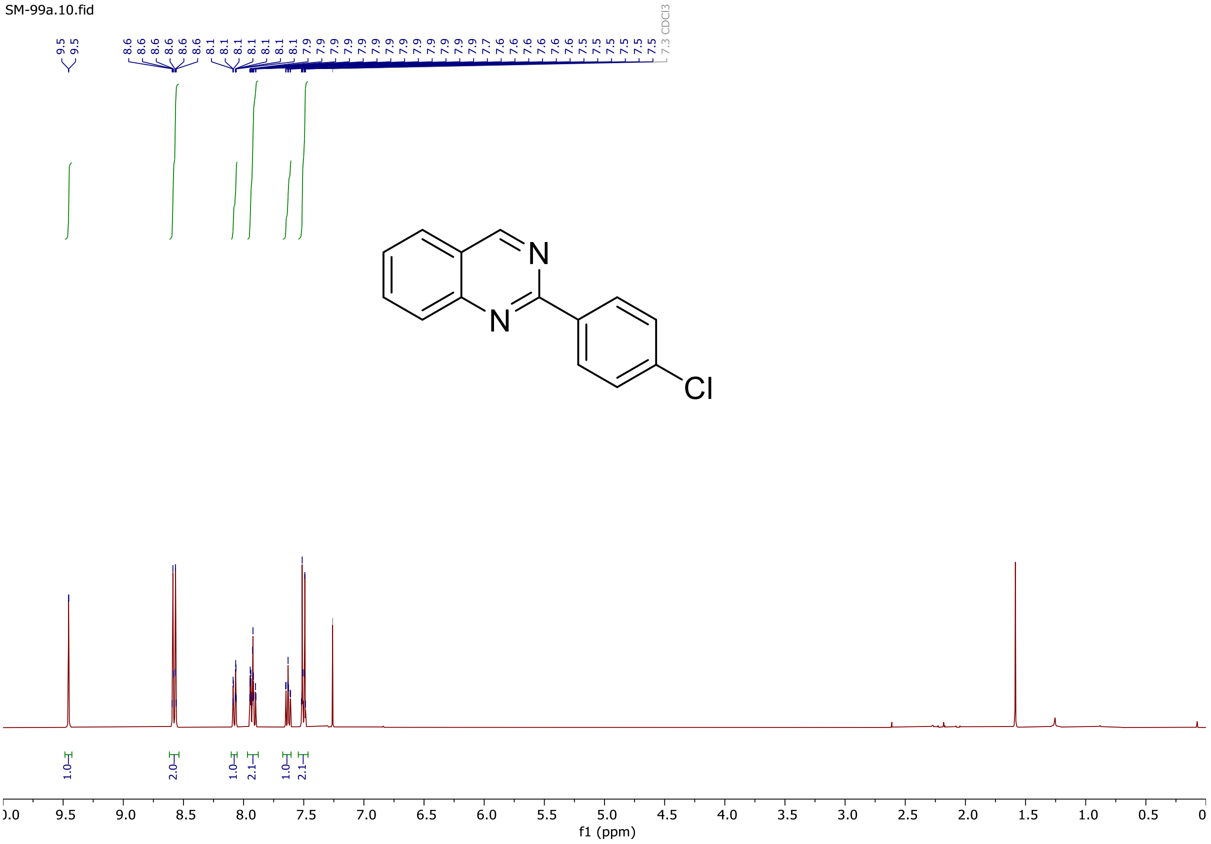

^{13}C NMR of **11c** (101 MHz, $CDCl_3$)

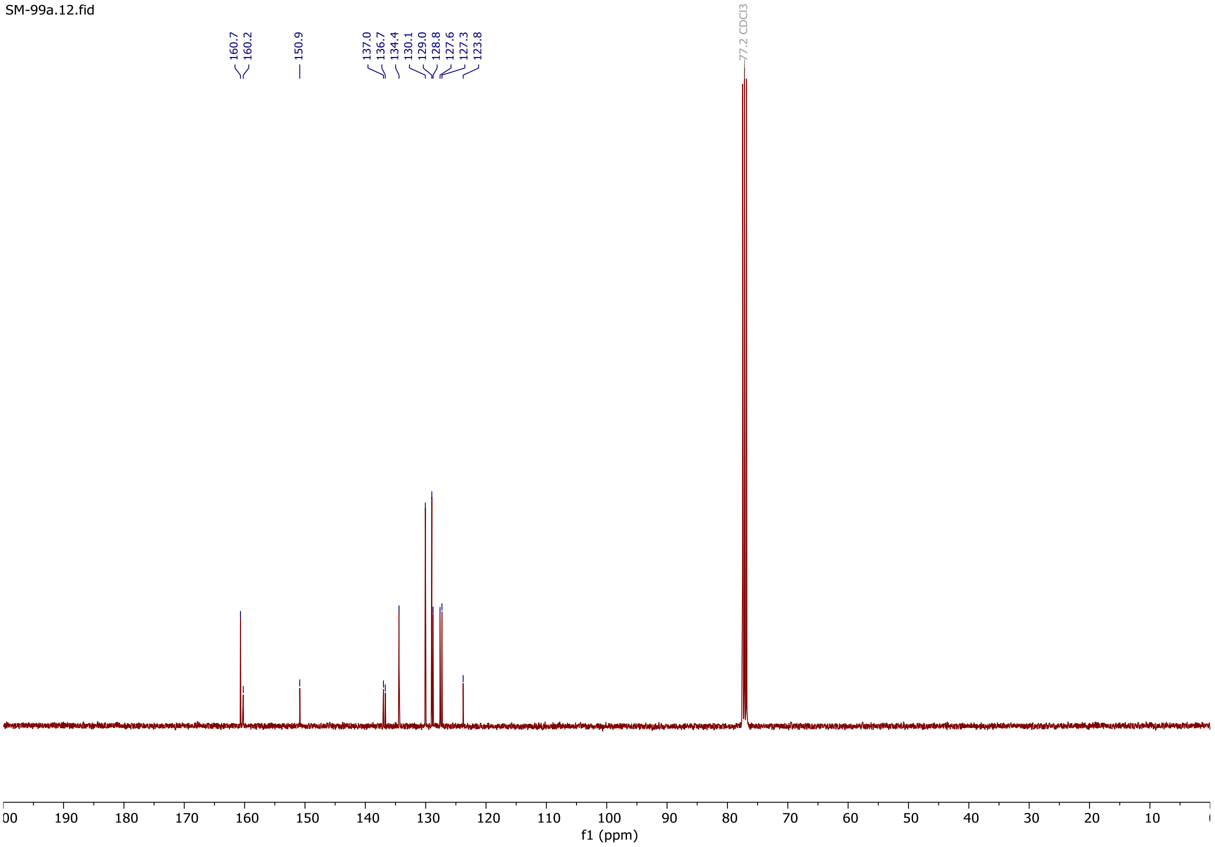

^{1}H NMR of **14a** (400 MHz, DMSO-D_6)

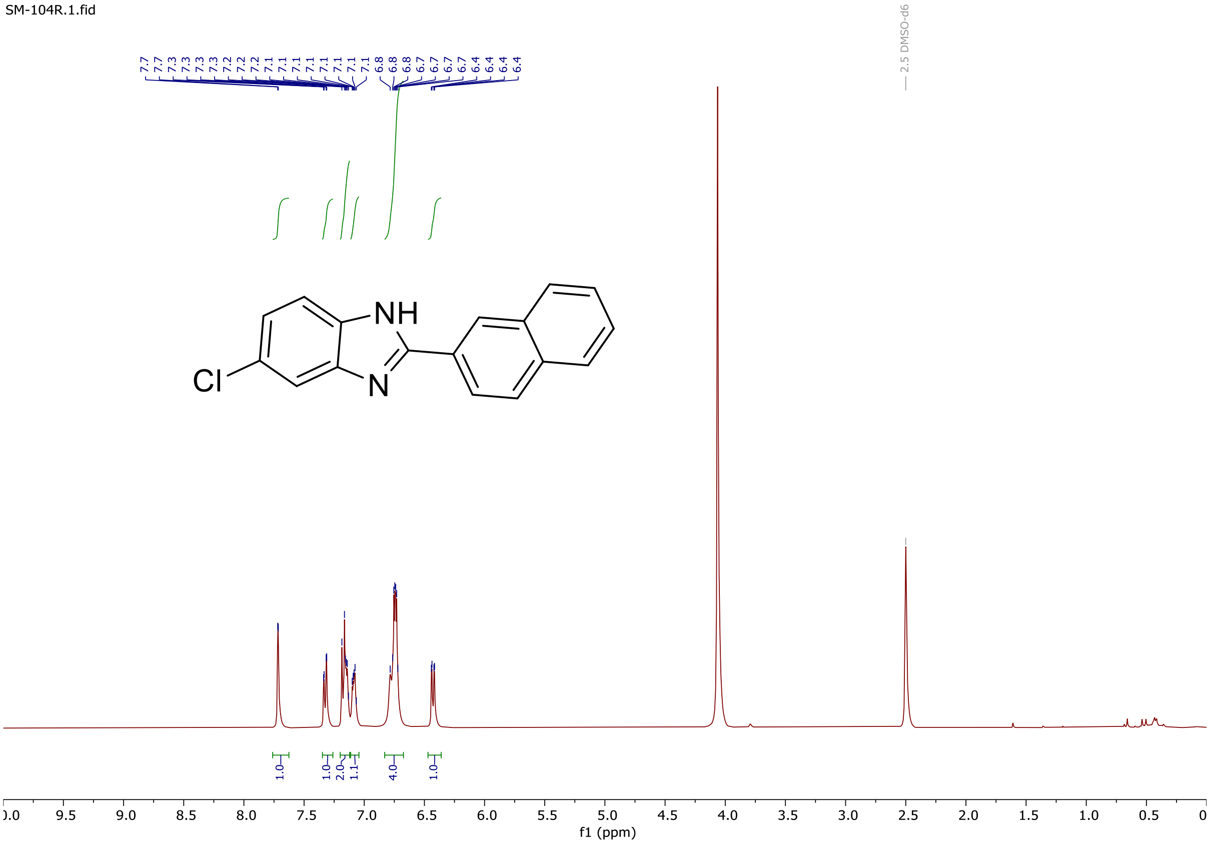

^{13}C NMR of **14a** (101 MHz, DMSO-D$_6$)

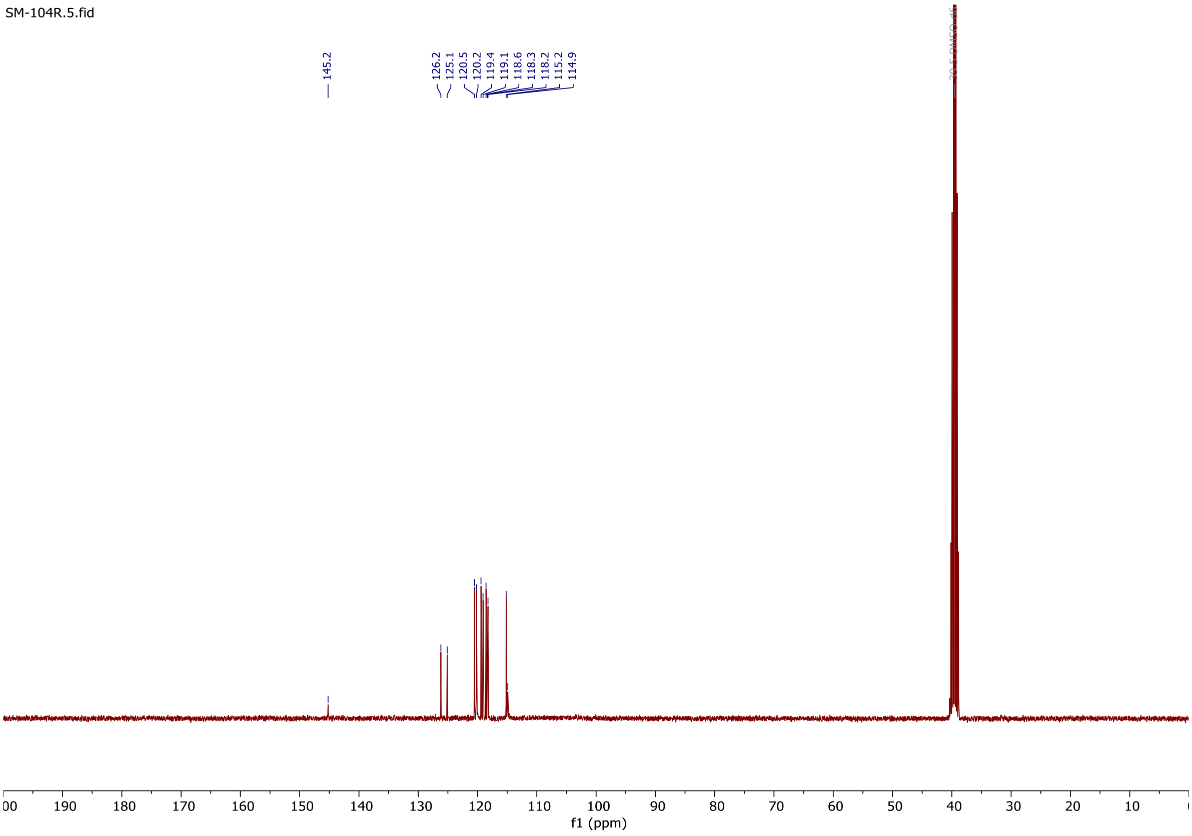

^{1}H NMR of **14b** (400 MHz, CDCl$_3$)

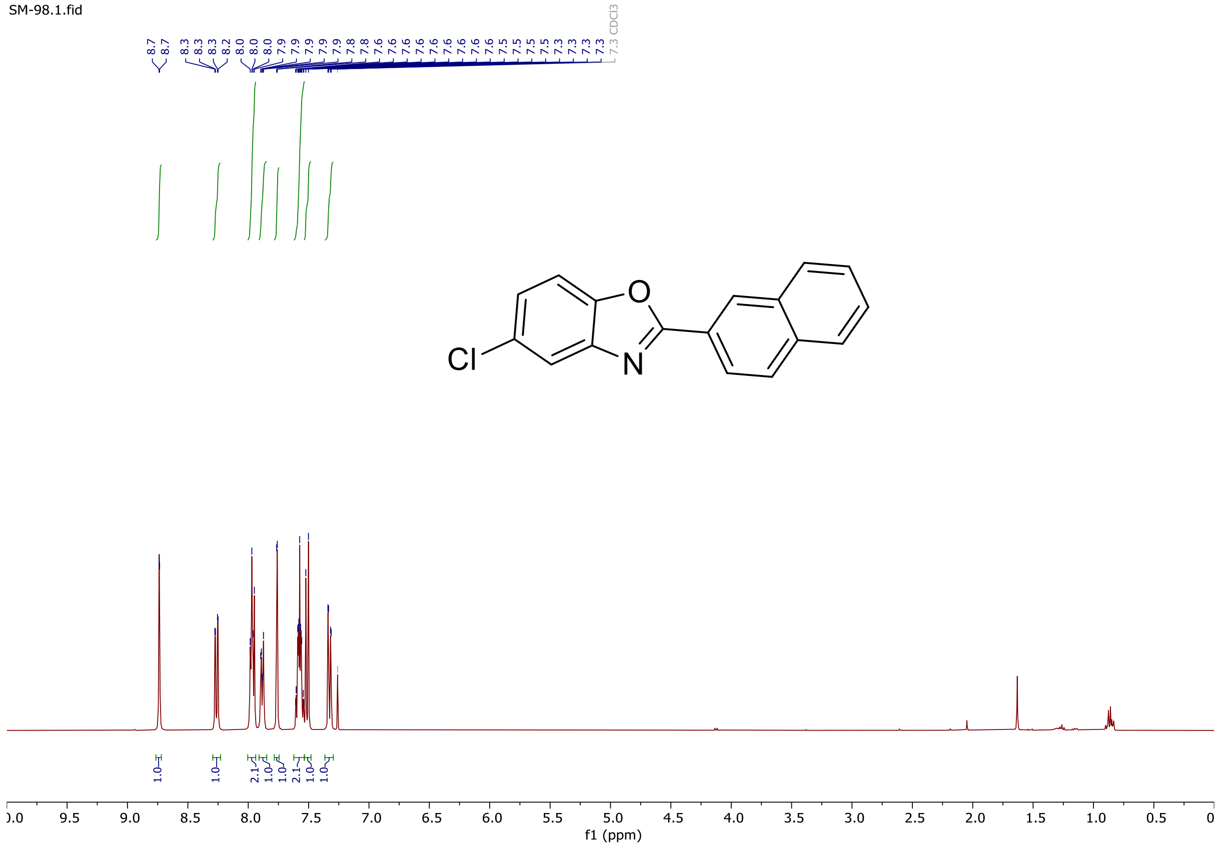

^{13}C NMR of **14b** (101 MHz, $CDCl_3$)

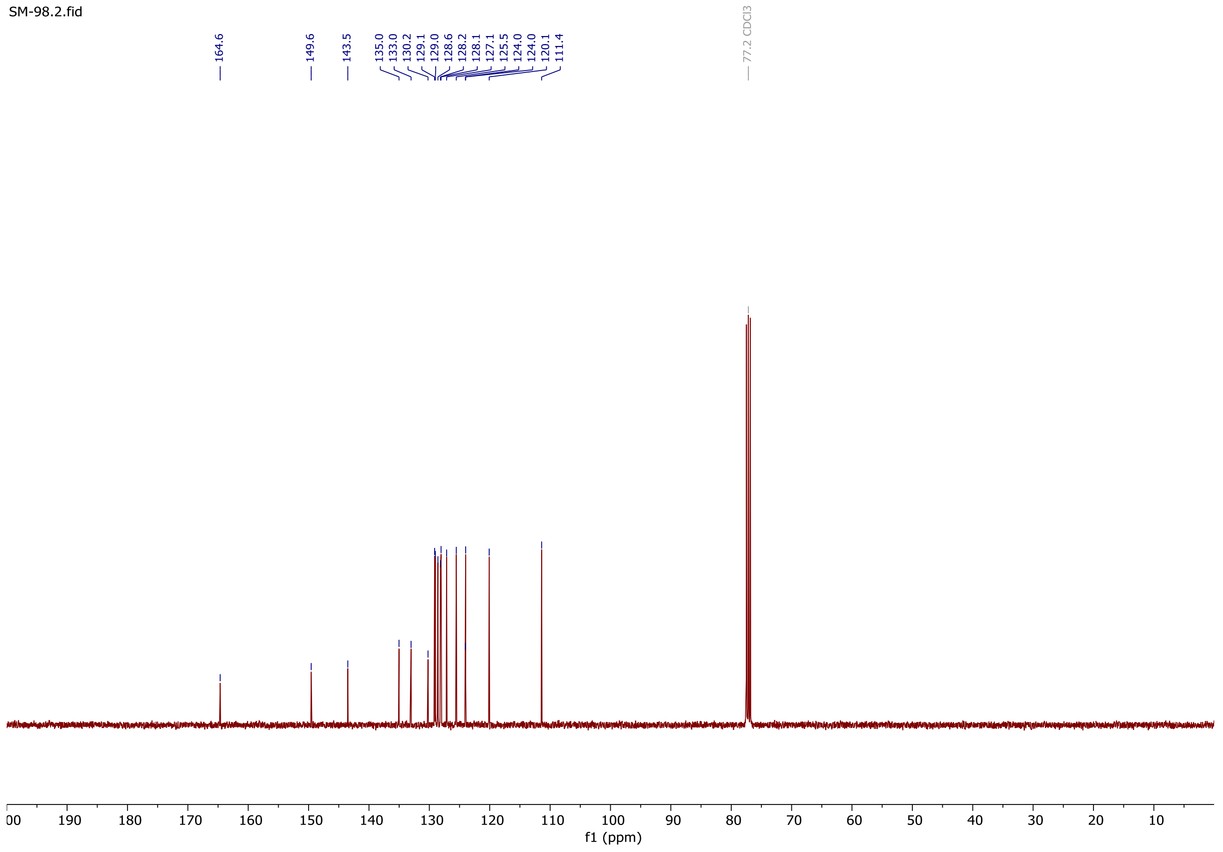

^{1}H NMR of **14c** (400 MHz, $CDCl_3$ and 3 drop of CD_3OD)

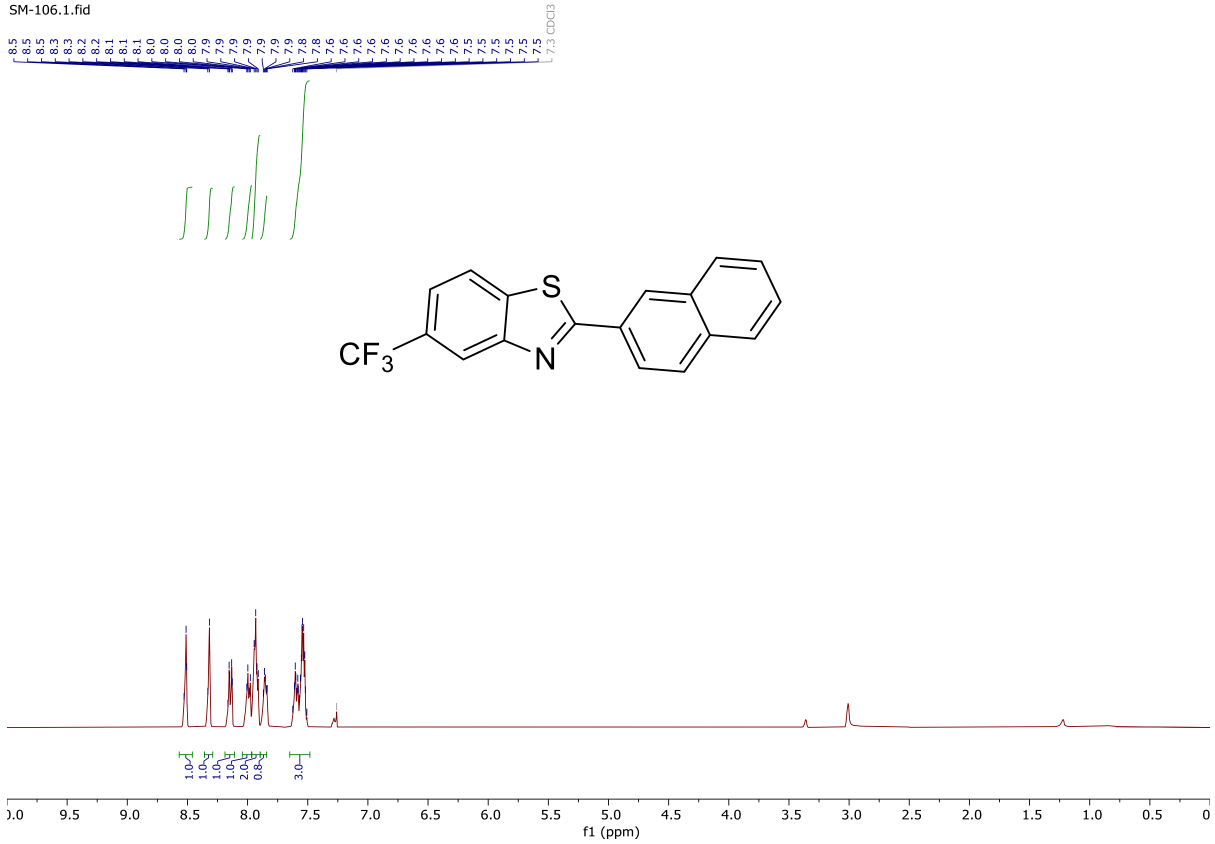

^{13}C NMR of **14c** (101 MHz, $CDCl_3$ and 3 drop of CD_3OD)

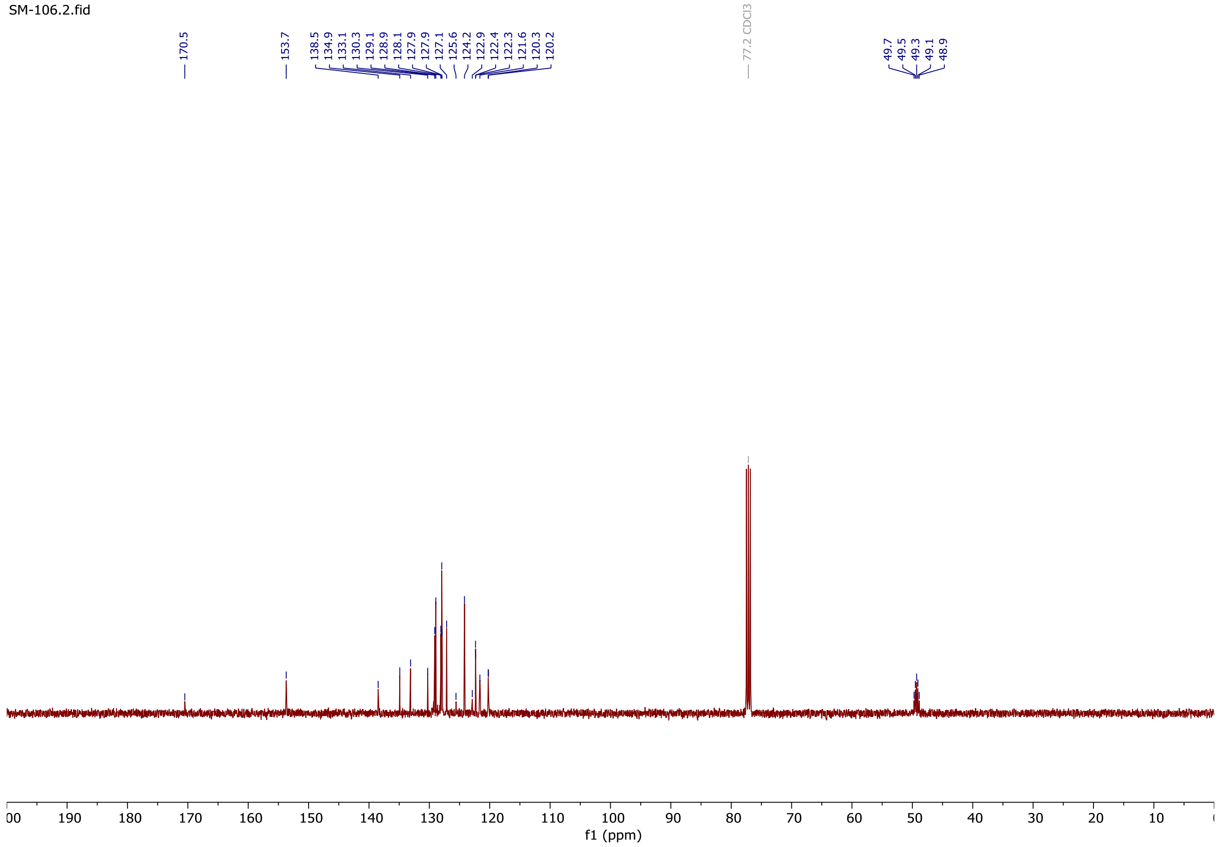

References:

[1] T. W. Funk, A. R. Mahoney, R. A. Sponenburg, K. P. Zimmerman, D. K. Kim, E. E. Harrison, *Organometallics* **2018**, *37*, 1133-1140.
[2] E. V. Johnston, E. A. Karlsson, L.-H. Tran, B. Åkermark, J.-E. Bäckvall, *Eur. J. Org. Chem.* **2009**, *2009*, 3973-3976.
[3] A. E. Wendlandt, S. S. Stahl, *J. Am. Chem. Soc.* **2014**, *136*, 11910-11913.
[4] X. Cui, Y. Li, S. Bachmann, M. Scalone, A.-E. Surkus, K. Junge, C. Topf, M. Beller, *J. Am. Chem. Soc.* **2015**, *137*, 10652-10658.
[5] Y. Xu, X. Huang, G. Lv, R. Lai, S. Lv, J. Li, L. Hai, Y. Wu, *Eur. J. Org. Chem.* **2020**, *2020*, 4635-4638.
[6] J. Wu, C. Darcel, *J. Org. Chem.* **2021**, *86*, 1023-1036.
[7] M.-Y. Chang, H.-Y. Chen, Y.-L. Tsai, *Org. Lett.* **2019**, *21*, 1832-1836.
[8] A. V. Iosub, S. S. Stahl, *Org. Lett.* **2015**, *17*, 4404-4407.
[9] Z. Ma, T. Song, Y. Yuan, Y. Yang, *Chem. Sci.* **2019**, *10*, 10283-10289.
[10] G. Jaiswal, V. G. Landge, D. Jagadeesan, E. Balaraman, *Nat. Commun.* **2017**, *8*, 2147.
[11] R. Jia, B. Li, X. Zhang, X. Fan, *Org. Lett.* **2020**, *22*, 6810-6815.
[12] J.-J. Zhong, W.-P. To, Y. Liu, W. Lu, C.-M. Che, *Chem. Sci.* **2019**, *10*, 4883-4889.
[13] D. Zhang, T. Iwai, M. Sawamura, *Org. Lett.* **2020**, *22*, 5240-5245.
[14] V. Humne, Y. Dangat, K. Vanka, P. Lokhande, *Org. Biomol. Chem.* **2014**, *12*, 4832-4836.
[15] K. Błaszczak-Swiatkiewicz, E. Mikiciuk-Olasik, *Acta Pol Pharm* **2013**, *70*, 451-458.
[16] R. R. Putta, S. Chun, S. H. Choi, S. B. Lee, D.-C. Oh, S. Hong, *J. Org. Chem.* **2020**, *85*, 15396-15405.
